# Sanqi Oral Solution Mitigates Proteinuria in Rat Passive Heymann Nephritis and Blocks Podocyte Apoptosis *via* Nrf2/HO-1 Pathway

**DOI:** 10.3389/fphar.2021.727874

**Published:** 2021-11-19

**Authors:** Xiaowan Wang, Jinchu Liu, Ruimin Tian, Bidan Zheng, Chuang Li, Lihua Huang, Zhisheng Lu, Jing Zhang, Wei Mao, Bo Liu, Kun Bao, Peng Xu

**Affiliations:** ^1^ State Key Laboratory of Dampness Syndrome of Chinese Medicine, The Second Affiliated Hospital of Guangzhou University of Chinese Medicine, Guangzhou, China; ^2^ Department of Nephrology, Guangdong Provincial Hospital of Chinese Medicine, Guangzhou, China; ^3^ Guangdong Provincial Key Laboratory of Chinese Medicine for Prevention and Treatment of Refractory Chronic Diseases, Guangzhou, China; ^4^ Guangdong Provincial Academy of Chinese Medical Sciences, Guangzhou, China; ^5^ Guangzhou Key Laboratory of Chirality Research on Active Components of Traditional Chinese Medicine, Guangzhou, China

**Keywords:** Sanqi oral solution, passive Heymann nephritis, podocyte apoptosis, proteinuria, Nrf2/HO-1 signaling pathway

## Abstract

Idiopathic membranous nephropathy (IMN) is the most common pathological type in adult nephrotic syndrome where podocyte apoptosis was found to mediate the development of proteinuria. Sanqi oral solution (SQ), an effective Chinese herbal preparation clinically used in treatment of IMN for decades, plays an important role in reducing proteinuria, but the underlying mechanisms have not been fully elucidated yet. The current study tested the hypothesis that SQ directly lessens proteinuria in IMN by reducing podocyte apoptosis. To investigate the effects of SQ, we established the experimental passive Heymann nephritis (PHN) rat model induced by anti-Fx1A antiserum *in vivo* and doxorubicin hydrochloride (ADR)-injured apoptotic podocyte model *in vitro*. SQ intervention dramatically reduced the level of proteinuria, together with the rat anti-rabbit IgG antibodies, complement C3, and C5b-9 deposition in glomerulus of PHN rats, accompanied by an elevation of serum albumin. Protein expression of synaptopodin, marker of podocyte injury, restored after SQ administration, whereas the electron microscopic analysis indicated that fusion of foot processes, and the pachynsis of glomerular basement membrane was markedly diminished. Further studies showed that SQ treatment could significantly inhibit podocyte apoptosis in PHN rats and ADR-injured podocytes, and protein levels of Cleaved Caspase-3 or the ratio of Bax/Bcl-2 were significantly decreased with SQ treatment *in vivo* or *in vitro*. Moreover, we found that the nuclear factor erythroid 2–related factor-2/heme oxygenase 1 (Nrf2/HO-1) pathway mediated the anti-apoptosis effective of SQ in podocyte. Thus, SQ mitigates podocyte apoptosis and proteinuria in PHN rats *via* the Nrf2/HO-1 pathway.

## Introduction

Idiopathic membranous nephropathy (IMN) is a unique glomerulonephritis, and remains the leading identifiable pathological type of nephrotic syndrome in adults (van de Logt et al., 2019). In recent years, only the morbidity of IMN has increased yearly compared with other glomerular diseases, and approximately 30–40% of IMN patients may develop end-stage kidney disease (Xu et al., 2016). The natural IMN progression, marked by “classically the rule of thirds” (one third spontaneous remission, one third persistent proteinuria, and one third progressive renal failure), is heterogeneous (Sinico et al., 2016). Considering the distinction of prognosis, it is proposed that different therapeutic regimens should be established according to the three risk groups of IMN (“low risk” group, “medium risk” group, and “high risk” group) classified by the Toronto Risk Score, and the treatment regimens are observation, immunosuppressive treatment (IST) after observation, and immediate IST (Angioi et al., 2018). Therefore, a long observative phase (at least 6 months) that needs to assess the risk score will extend delay for the start of IST (van den Brand et al., 2012). Inappropriate medical decision for evaluation of risk groups may result in delay of treatment or increase unnecessary side effects of IST (Xie et al., 2015). Nevertheless, the predicament provides a therapeutic window for the treatment of IMN with traditional Chinese medicine.

Traditional Chinese medicine can effectively promote the remission of IMN (Chen et al., 2013; Liu B. et al., 2019; Feng et al., 2020). Sanqi oral solution (SQ), mainly composed of Radix Astragali [*Astragalus mongholicus Bunge [Fabaceae]*] and Radix Notoginseng [*Panax notoginseng (Burkill) F.H. Chen [Araliaceae]*], is an effective hospital preparation of Guangdong Provincial Hospital of Chinese Medicine developed in 1995 for the treatment of chronic glomerulonephritis. Besides, the therapeutic effect of SQ on IMN patient is positive in clinics. It is confirmed in our previous studies that the sanative target of SQ in ameliorating proteinuria of IMN is podocyte (Tian et al., 2019). The present study aims to further explore the inherent mechanism of SQ in protecting podocyte and alleviating albuminuria in IMN.

IMN is characterized morphologically by the accumulation of immune complexes on the subepithelial aspect of the glomerular capillary loops with glomerular basement membrane (GBM) thickening, and functionally by a marked increase of protein excretion (Liu W. et al., 2019). As the barrier of the glomerular filtration, podocyte plays irreplaceable roles in preventing the formation of albuminuria together with silt diaphragms (Yuan et al., 2020). Podocytes are major target cells of assembled C5b-9 triggered by immune complexes in the development of IMN (Ronco and Debiec, 2020). As terminal differentiated cells, podocytes are prone to death when injured by stimulators including C5b-9 (Nangaku et al., 2005). As an important form of cell death, apoptosis has been known to be deeply involved in podocyte death under IMN condition (Sun et al., 2021). Reducing apoptosis could effectively restrain podocyte loss and albuminuria production in proteinuric kidney disease including IMN (Burlaka et al., 2016). Recent studies indicated that heme oxygenase 1 (HO-1) is involved in the regulation of podocyte apoptosis. As an isoform of heme oxygenase (HO), HO-1 serves as the microsomal rate limiting enzyme of heme catabolism and modulator of biological processes (Facchinetti, 2020). Upregulated HO-1 was found in kidney under different kinds of pathological conditions, such as IMN and diabetic nephropathy (Lee et al., 2009; Sutariya et al., 2017). Inhibition of HO-1 can accumulate apoptotic podocytes *in vivo* and *in vitro*, while early induction of HO-1 could reduce proteinuria and protect kidney against injury (Dong et al., 2015). Besides, HO-1 is also known as downstream factor regulated by nuclear factor erythroid 2–related factor 2 (Nrf2) (Loboda et al., 2016). The activation and inhibition of the Nrf2/HO-1 signaling pathway play an important role in preventing apoptosis and promoting cell survival (Ji et al., 2021). Research has demonstrated that the induction of the Nrf2/HO-1 signaling pathway is linked to diminishment of podocyte loss, immune complex deposition, and kidney injury in the passive Heymann nephritis (PHN) model (Sutariya et al., 2017).

Accordingly, in the present study, we probed into the potential effects of SQ on podocyte apoptosis in IMN rats and doxorubicin hydrochloride (ADR)–injured apoptotic podocytes, and made further investigation into the role of the Nrf2/HO-1 signaling pathway within it. The current results will offer a new connotation for clinical application of SQ in IMN.

## Materials and Methods

### Chemicals and Reagents

SQ (Batch No. 210102; Cantonese medicine ratification No. Z20071155), compound cyclophosphamide tablets (Batch No. 180604, Country medicine ratification No. H22026738), and prednisone acetate tablets (Batch No. 1710150; Country medicine ratification No. H12020123) were provided by Guangdong Provincial Hospital of Chinese Medicine. Anti-Fx1A antiserum (#PTX-002S) was obtained from PROBETEX (San Antonio, United States). ADR (#HY-15142) and trigonelline (Trig, #HY-N0414) were obtained from MCE (New Jersey, United States). Primary antibodies against Caspase-3 (#14220), Bax (#2772), Nrf2 (#12721), GAPDH (#5174), and Histone H3 (#4499) and secondary antibodies against rabbit IgG (7074) and mouse IgG (7076) were purchased from Cell Signaling Technology Inc. (Beverly, MA, United States). Goat pAb to rat IgG (Alexa Fluor^®^ 488) (ab150165), primary antibodies against C3 (ab11887), Bcl-2 (ab196495), and HO-1 (ab13248) and secondary antibody against Goat pAb to Mouse IgG (Alexa Fluor^®^ 488) (ab150113) were purchased from ABCAM (Cambridge, MS, United States). Primary antibodies against C5b-9 (sc-66190) and synaptopodin (sc-515842) were purchased from Santa Cruz Biotechnology (Dallas, Texas, United States). One-step TUNEL apoptosis assay kit (C1089) was obtained Beyotime Biotechnology Institution (Jiangsu, Nanjing). 4′,6-Diamidino-2-phenylindole (DAPI) (BS097) was purchased from Biosharp Life Sciences (Hefei, China). Other reagents were of analytical grade from commercial suppliers.

### Preparation and Chemical Profiles of SQ

SQ is authorized for clinical treatment by the Drug Administration of Guangdong Province and chemically characterized and manufactured by Guangdong Provincial Hospital of Chinese Medicine (Guangzhou, China). The extraction method of Chinese Pharmacopoeia was applied to get SQ from Radix Astragali and Radix Notoginseng with a concentration of 0.333 and 0.056 g/ml, respectively, and the botanical name of Radix Astragali and Radix Notoginseng could be confirmed on https://mpns.science.kew.org/mpns-portal/. The botanical samples of SQ have been stored in the Pharmaceutical Preparation Department of Guangdong Provincial Hospital of Chinese Medicine, and are available whenever needed. Sanqi oral solution lyophilized power (SQL) was prepared according to following steps: SQ was frozen in culture dish (diameter: 100 mm) at −80°C for 48 h. Then SQ was dried in vacuum condition at −60 to 70°C to obtain SQL, and SQL was stored at −80°C before use. The preparation and chemical components analysis of SQ were performed referring to our published study (Tian et al., 2020). The content and chemical profiles of SQ are shown in Supplementary Table 1 and Supplementary Figure 1.

### Animals

Adult male Sprague-Dawley (SD) rats weighing between 180 and 220 g with a special pathogen-free (SPF) level were obtained by the Medical Experimental Animal Center of Guangdong Province (production certification No. SCXK 2019-0035, Guangdong). All the experimental rats were housed in Experimental Animal Center of Guangdong Provincial Hospital of Chinese Medicine (use certification No. SYXK 2018-0094, Guangdong) with 20 ± 2°C controlled temperature and 50 ± 10% humidity, and a 12-h light/dark cycle (lights on: 07:00–19:00), ventilation, more than 10 air exchanges per hour (all-fresh-air system), and allowed free access to standard laboratory water and diet *ad libitum*. All animal experiments were carried out as per the Regulations of Experimental Animal Administration issued by the State Committee of Science and Technology of the People’s Republic of China, with the approval of Animal Care and Use Committee in Guangdong Provincial Hospital of Chinese Medicine (Guangzhou, China).

### Induction of PHN and Experimental Schedule

**FIGURE 1:**
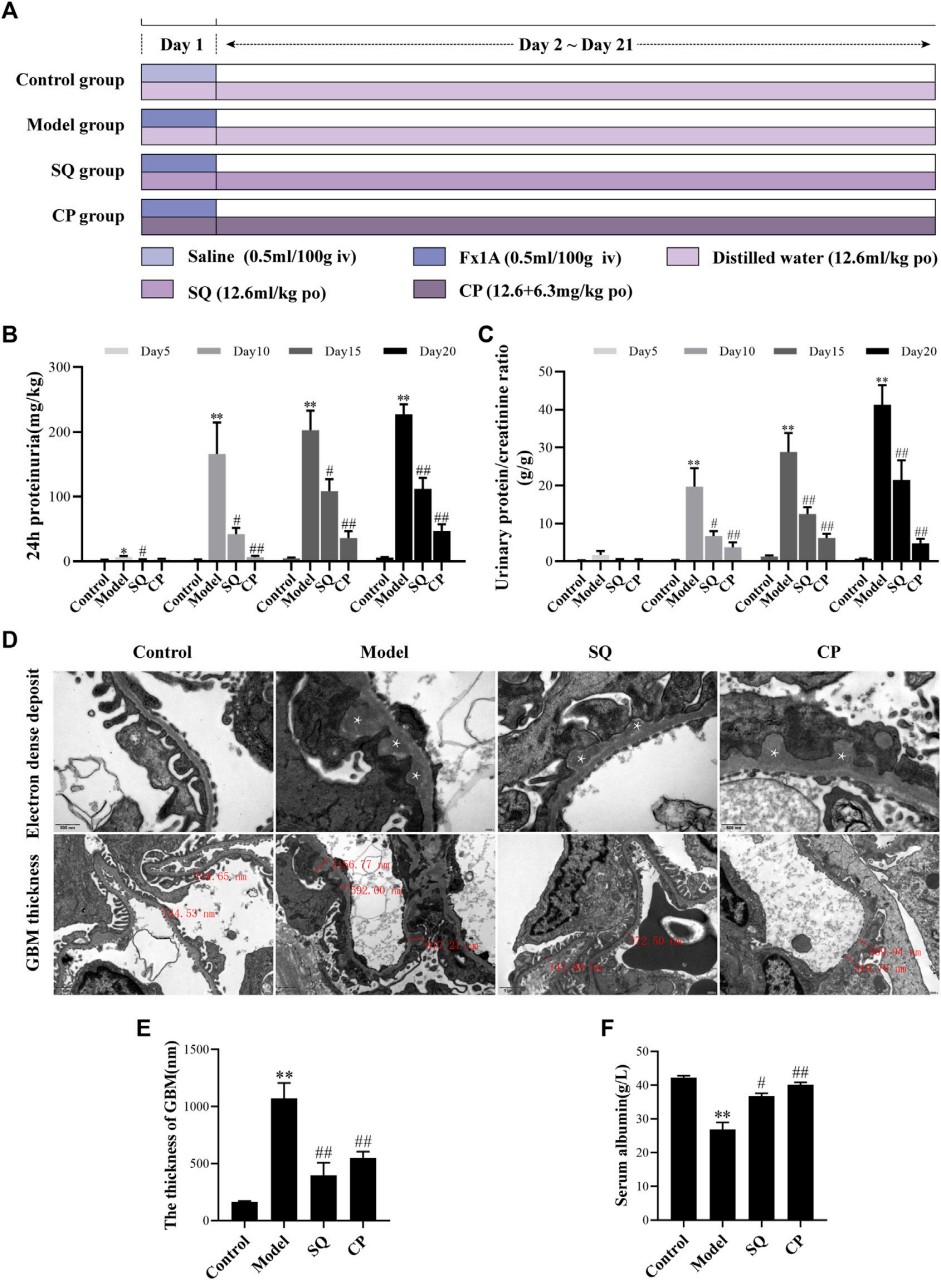
SQ diminished proteinuria and pathomorphologic injury in PHN rats. The experiment was implemented referring to the animal experimental progress schedule **(A)**. After SQ and CP intervention, the 24-h proteinuria **(B)** and the urinary protein/creatinine ratio **(C)** levels of PHN rats were significantly reduced and serum albumin **(F)** levels were markedly restored (*n* = 6). TEM examination of ultrathin kidney sections **(D)** showed changes of subepithelial glomerular immune deposits (magnification × 30,000, asterisks) and thickening of the GBM (magnification × 12,000) of rats in each group. Semi-quantification of GBM thickness **(E)** was analyzed (*n* = 6). Data are represented as mean ± SD from independent groups. ^*^ *p* < 0.05 vs. Control group. ^**^ *p* < 0.01 vs. Control group. ^#^ *p* < 0.01 vs. Model group. ^##^ *p* < 0.01 vs. Model group.

The PHN rat model was induced as previously described with a few modification (Di Tu et al., 2020). After acclimatization of 3 days, healthy adult SD rats were randomly divided into four groups (*n* = 6 per group): 1) Control group (Control); 2) Model group (Model); 3) SQ group (SQ); and 4) CP (Compound cyclophosphamide + Prednisone acetate) group (CP). Combination of compound cyclophosphamide tablets and prednisone acetate tablets was chosen as the positive control drug, and the intragastric dosage of SQ, complex cyclophosphamide together with prednisone acetate were calculated according to the clinical dosage using human–rat conversion coefficient. The SD rats of the PHN group, SQ group, and CP group were given a tail vein injection with a single dose of anti-Fx1A antiserum (0.5 ml/100 g), while the SD rats of the SQ group and the CP group were administrated with either SQ (12.6 mg/kg) or compound cyclophosphamide tablets (12.6 mg/kg) and prednisone acetate tablets (6.3 mg/kg) daily by oral gavage. When modeling and administration, the Control group and the PHN group were given the same amount of saline or distilled water. (Figure 1A) On day 5, 10, 15, and 20, the 24-h urine samples were recorded and collected for the urine protein analysis. After 21 days, all SD rats were sacrificed and required samples were collected.

### Biochemistry of Blood and Urine

Rats in all groups were put into separate metabolic cages to collect urine for 24 h. During the collection of urine, rats were allowed free to drink water but were limited to diet. The volume of 24-h urine was recorded after collection, and the collected urine samples were centrifuged at 3,000 rpm for 15 min at room temperature, the supernatants were stored at −80°C until analysis. After the experiment, plasma samples in rats were collected by abdominal aorta and put into coagulation-promoting vacuum tubes. The collected plasma samples were allowed to stand for 1 h at 4°C and centrifuged at 12,000 rpm for 15 min at 4°C to collect serum, and then the serum was stored at −80°C for a biochemical analysis. The urine protein and serum albumin (ALB) analyzed by the clinical laboratory of Guangdong Provincial Hospital of Chinese Medicine (Guangzhou, China).

### Transmission Electron Microscopy

Examination of transmission electron microscopy (TEM) was conducted as previously described with a few modifications (Wang et al., 2020). Briefly, the sections of kidney tissues (1 mm^3^) were fixed in 5% glutaraldehyde for 2 h, followed by washing in 0.1 M phosphate buffer. After immersing in 1% osmic acid for 1.5–2 h, the kidney tissues were dehydrated through graded alcohols and immersed in embedding medium overnight, and then, the immersed sections were embedded in Epon 812 and dried in an oven. The dried kidney sections were cut into 50–70 nm slices and stained with uranyl acetate and lead citrate. A transmission electron microscope (JEM1400 PLUS, Japan) was used to examine the slices at 100 kV, and a CCD camera (EMSIS VELETA G3, Germany) was used to take micrograph.

For evaluating the foot process width per GBM length, images covering a glomerular cross section were captured by TEM. The peripheral length of GBM was measured, and the quantity of foot process overlying this part of GBM was counted using Adobe Photoshop (San Jose, California, United States). The arithmetic mean of the foot process width was calculated by the following formula (Chen et al., 2010) (1):

$$The\;foot\;process\;width\;=\;\left(\pi /4\right)\;\times \frac{\sum GBM\;length}{\sum quantity\;of\;foot\;process},$$ (1)

where ∑GBM length and ∑quantity of foot process represented the total GBM length measured in one glomerulus and the total quantity of foot process counted, respectively. The correction coefficient π/4 is set to correct the random orientation in which the foot processes are sectioned.

Basement membrane thickness in each ultrathin slice was measured by RADIUS software (Boston, MA, United States). Briefly, an open capillary loop was selected, and the value perpendicular to the GBM overlying this capillary loop length was metered.

### Immunofluorescence Staining

Tissues and immunofluorescence staining were performed according to the method previously described with a few adjustments (Li et al., 2021). Briefly, renal tissues were embedded in Tissue-Tek OCT compound, snap frozen in liquid nitrogen, and stored at −80°C till examination. To inspect the deposition of IgG, C3, C5b-9, and synaptopodin in glomeruli, 5 µm cryosections were immersed in acetone and washed in cold PBS. After blocking with 5% bovine serum albumin in PBS for 30 min, the cryosections were stained with IgG, C3, C5b-9, and synaptopodin overnight at 4°C, followed by goat anti-mouse IgG H&L (Alexa Fluor^®^ 488). The cryosections were observed under confocal fluorescence microscope (Zeiss LSM710, Germany). Fluorescence intensity was measured in five randomly selected fields of six cryosections by ImageJ (Bio-Rad Laboratories, Hercules, CA, United States).

### TUNEL Staining

The genomic DNA in the nucleus cracks after apoptosis. With terminal deoxynucleotidyl transferase (TdT) as catalyst, exposed 3′- OH binds to fluorescent probe Cy3 labeled dUTP, which can be tested by fluorescence confocal microscopy. Cell apoptosis was detected by the TUNEL assay kit, referring to the manufacture’s protocol. Briefly, renal slices (3 μm) of different groups were dewaxed, followed by incubation with protease K (without DNase, 20 μg/ml) for 30 min at 37°C and washing with cold PBS, and then the kidney sections were incubated with 50 μL TUNEL reaction mixture for 1 h at 37°C in dark. Ultimately, stained slices were washed with PBS and examined by confocal fluorescence microscope (Zeiss LSM710, Germany). Five fields of six sections from each group were randomly selected for analysis.

### Podocyte Culture and Treatment

The conditionally immortalized temperature sensitive mouse podocyte cell line used in this study was established by Professor Peter Mundel (Medical College of Harvard University, Boston, MA, United States). Briefly, podocytes were cultured in RPMI 1640 medium with 10% fetal bovine serum at 33°C in the presence of 10 U/ml recombinant mouse interferon-γ (Sigma, St. Louis, MO, United States). For inducing differentiation, podocytes were thermoshifted to 37°C and cultured in interferon-free medium for 10–14 days. SQL and Trig were dissolved in PBS and storage solutions were stored at −20°C. Podocytes were cultured overnight before experiments and treated with 400 ng/ml ADR with or without 25 μM Nrf2 inhibitor Trig or 600 μg/ml SQL intervention for 24 h.

### Western Blot

Western blot was conducted as previously described (Wang XW. et al., 2019). Total proteins from renal tissues or podocyte cells were extracted using the T-PER™ Tissue Protein Extraction Reagent (Thermo Fisher Scientific, Rockford, IL, United States) with proteinase and phosphatase inhibitor tablets (Roche, Mannheim, Germany) or the RIPA Lysis Buffer (Beyotime, Shanghai, China). The nuclear proteins in kidneys and podocyte cells were prepared with the Nuclear Protein Extraction Kit (Beyotime, Shanghai, China), according to the protocols. Protein concentration was measured by the Pierce^®^ BCA Protein Assay Kit (Thermo Fisher Scientific, Rockford, IL, United States). Lysates were boiled with a 5 × loading buffer (CWBIO, Beijing, China), separated by 10–12.5% SDS–polyacrylamide gel electrophoresis, and transferred onto a PVDF membrane (Millipore, United States). After blocking with 5% non-fat milk in Tris-HCL buffer containing in Tween 20 (TBST), the membranes were incubated at 4°C with corresponding primary antibodies (Nrf2, HO-1, Bcl-2, Bax, Cleaved Caspase-3, Caspase-3, and GAPDH) overnight. Then the membranes were washed with TBST and incubated with horseradish peroxidase (HRP)–labeled anti-rabbit or anti-mouse IgG at room temperature. Blots were developed detected by Immobilon Western Chemiluminescent HRP Substrate (Millipore, Billerica, United States), referring to the manufacturer’s protocol, visualized by enhanced chemiluminescence detection system (Bio-Rad, Laboratories, Hercules, CA, United States), and quantified using Image Lab software 5.2.1 (Bio-Rad, Laboratories, Hercules, CA, United States).

### Statistical Analysis

All quantitative data were expressed as mean ± standard deviation (SD). One-way analysis of variance (ANOVA) followed by Dunnett’s T3 test was used to compare group mean using SPSS 25.0 (SPSS, Inc., Chicago, IL, United States). *p* < 0.05 was considered statistically significant, and *p* < 0.01 was considered statistically highly significant.

## Results

### SQ Reduced Proteinuria in PHN Rats

Proteinuria, a general characteristic of IMN, was observed in rats of each group. Present data revealed that PHN rats developed severe proteinuria, and 24-h proteinuria was significantly elevated from Day 5. However, the secretion of proteinuria in PHN rats was found to be decreased remarkedly at all points of observation, including Day 5, Day 10, Day 15, and Day 20 (Figure 1B). Similarly, the urinary protein/creatinine ratio was increased in PHN rats from Day 5, and intervention of SQ and CP could reduce the ratio significantly from Day 10 (Figure 1C). Besides, the level of serum albumin was found to be reduced significantly in PHN rats (*p* < 0.01), whereas the serum albumin level was increased markedly with the treatment of SQ and CP (*p* < 0.05 and *p* < 0.01) (Figure 1F).

### SQ Ameliorated Glomerular Pathomorphology in PHN Rats

The changes of glomerular pathomorphology are shown in Figures 1D,E. Transmission electron microscopic examination showed subepithelial glomerular immune deposits and thickening of the GBM (*p* < 0.01) in glomeruli of rats with PHN, while treatment with SQ and CP, less thickened GBM were visible, compared to PHN rats without the treatment of SQ or CP (*p* < 0.01). The data above indicated that SQ showed therapeutic effect in anti-Fx1A antiserum induced PHN in rats.

### SQ Decreased IgG, C3, and C5b-9 Deposition in PHN Rats

**FIGURE 2:**
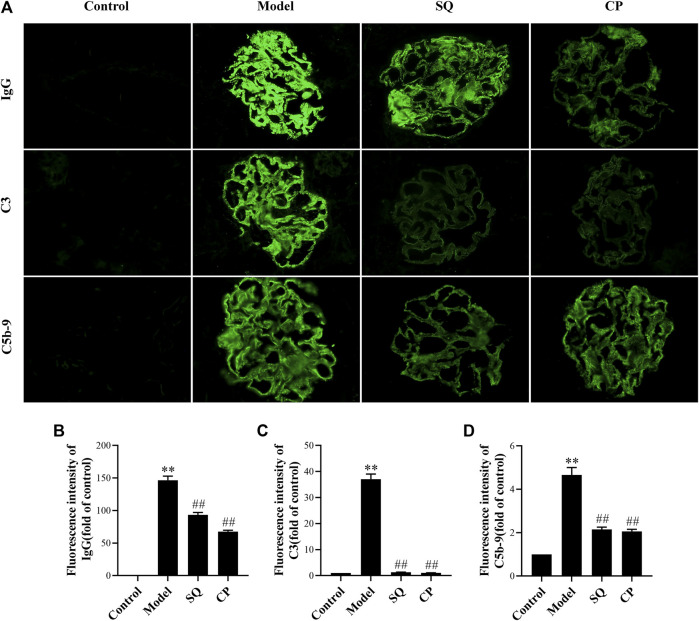
SQ reduced IgG, C3, and C5b-9 deposition in glomeruli of PHN rats (magnification × 400). Kidney cryosections in each group were detected for IgG, C3, and C5b-9 by immunofluorescence staining **(A)**. Quantification of the fluorescence intensity of IgG **(B)**, C3 **(C)**, and C5b-9 **(D)** was assessed (*n* = 6). Data are represented as mean ± SD from independent groups. ^**^ *p* < 0.01 vs. Control group. ^##^ *p* < 0.01 vs. Model group.

According to the pathogenesis of the rat PHN model, the antibodies in rabbit anti-Fx1A antiserum occur in the kidney and recognize the rat autologous antigen megalin, which exists on podocyte (Zanetti and Druet, 1980; Raychowdhury et al., 1996), and then formed glomerular *in situ* immune deposits on the subepithelial layer of the glomerular capillary loops. Deposition of immune complexes stimulated the complement system and induced the assembling of C5b-9 membrane attack complex (MAC) in glomeruli. As in our study, PHN rats displayed pronounced granular autologous IgG deposition in glomeruli, dispersing along the capillary wall (*p* < 0.01) (Figures 2A,B), and compared to PHN rats, IgG deposition was diminished remarkably by SQ and CP intervention (*p* < 0.01). It is known that the complement system plays a major role in the course of IMN. In human IMN, the renal deposition of C3 and C5b-9 depositions is typical, and they are also found in PHN rats (Raychowdhury et al., 1996). Immunofluorescent staining of C3 and C5b-9 were also observed in glomeruli (Figures 2A,C,D). Compared with the control group, C3 and C5b-9 were particularly expressed along the capillary walls in glomerular of PHN rats (*p* < 0.01). In contrast, SQ and CP treatment significantly decreased the deposition of C3 and C5b-9 (*p* < 0.01), suggesting that SQ could alleviate the immune injury in PHN rats.

### SQ Restrained Glomerular Podocyte Injuries in PHN Rats

**FIGURE 3:**
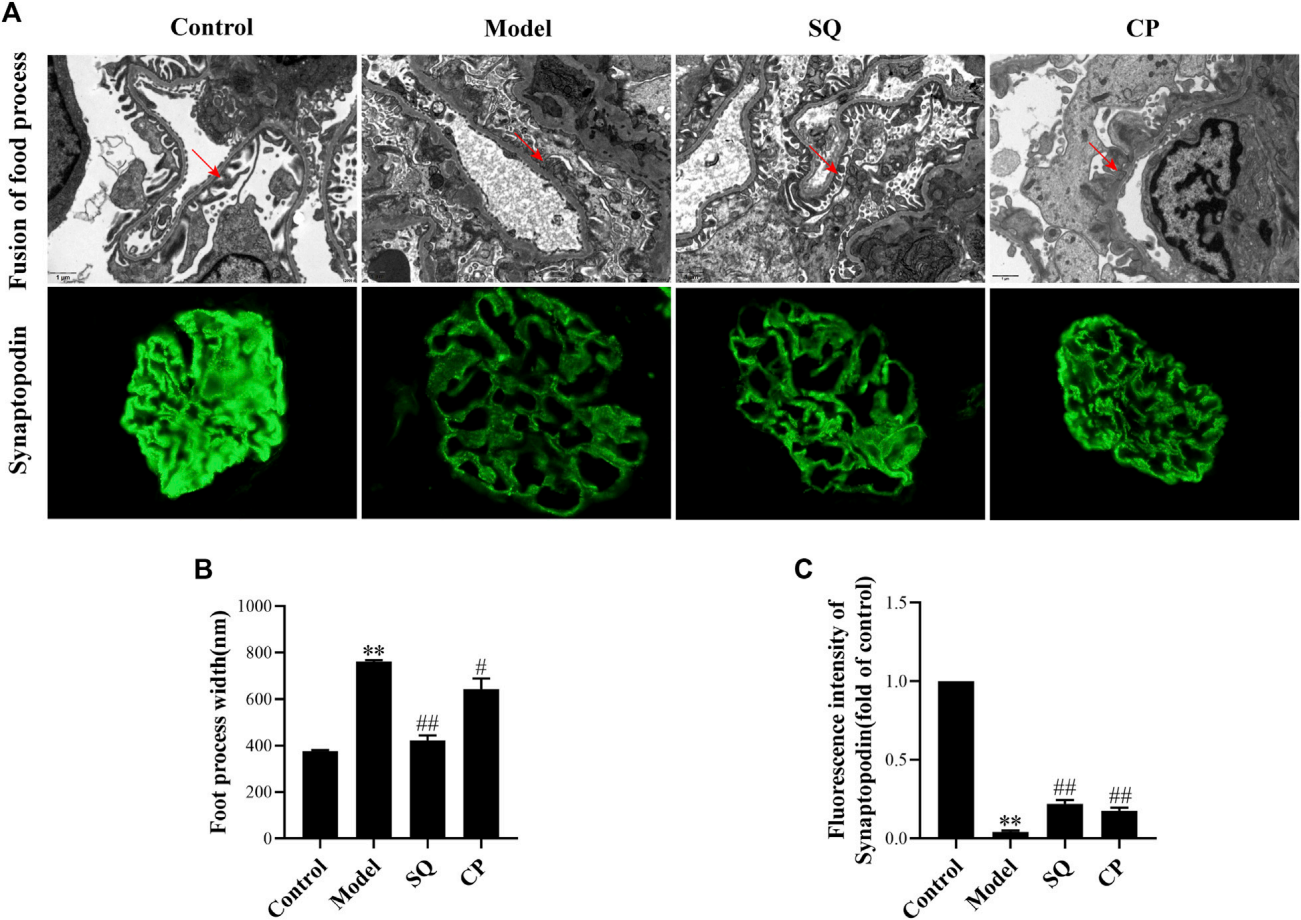
SQ alleviated glomerular podocyte injury in PHN rats. Effects of SQ and CP on foot process width (magnification × 12,000, red arrows) and synaptopodin expression (magnification × 400) were measured by TEM and immunofluorescence staining **(A)**. With the treatment of SQ and CP, restored glomerular podocytic foot processes **(B)** and synaptopodin expression **(C)** were seen in PHN rats (*n* = 6). Data are represented as mean ± SD from independent groups. ^**^ *p* < 0.01 vs. Control group. ^##^ *p* < 0.01 vs. Model group.

Podocyte, also called the visceral glomerular epithelial cell, is the key part of glomerular filtration barrier. Podocyte injury can induce proteinuria directly. Diffuse fusion of podocyte foot process was observed in PHN rats under TEM (Figure 3A). A semi-quantificative analysis of foot process width showed that glomerular podocyte food processes were ameliorated partially in PHN rats with the treatment of SQ or CP (Figure 3B). In addition, synaptopodin is an actin-associated protein that plays important role in the morphology and movement of podocytes based on actin, and is considered as a marker of differentiation and maturation. As shown in Figures 3A,C, the reduced expression of synaptopodin was observed in PHN rats (*p* < 0.01). However, intervention with SQ or CP significantly restored the downregulated glomerular synaptopodin (*p* < 0.01). The results demonstrated that SQ protects against podocyte injury in PHN rats.

### Effects of SQ on Renal Podocyte Apoptosis *in Vivo* and *in Vitro*

**FIGURE 4:**
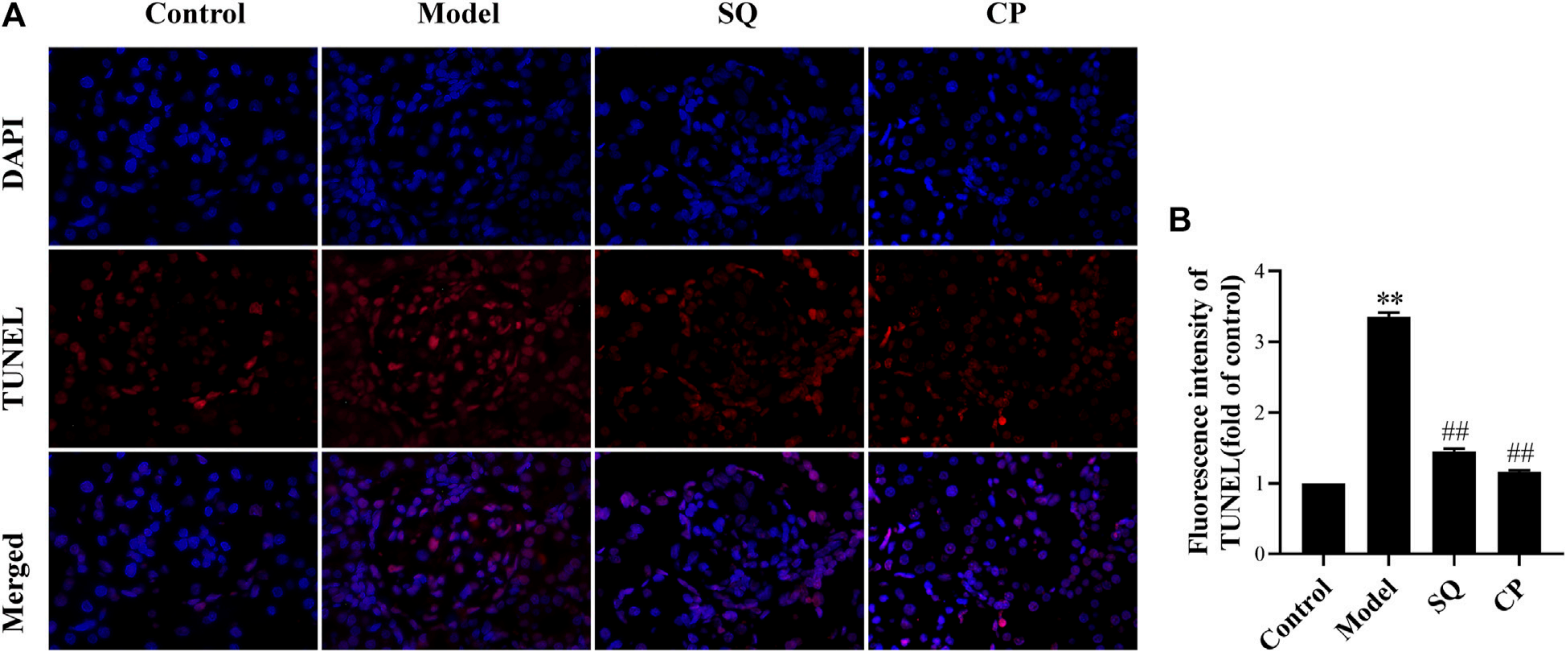
SQ restrained podocyte apoptosis in PHN rats (magnification × 200). Kidney paraffin slices were stained by TUNEL assay with labeled dUTP and DAPI dye **(A)**. Podocyte apoptosis **(B)** in PHN rats effectively deteriorated with the treatment of SQ and CP (*n* = 6). Data are represented as mean ± SD from independent groups. ^**^ *p* < 0.01 vs. Control group. ^##^ *p* < 0.01 vs. Model group.

**FIGURE 5:**
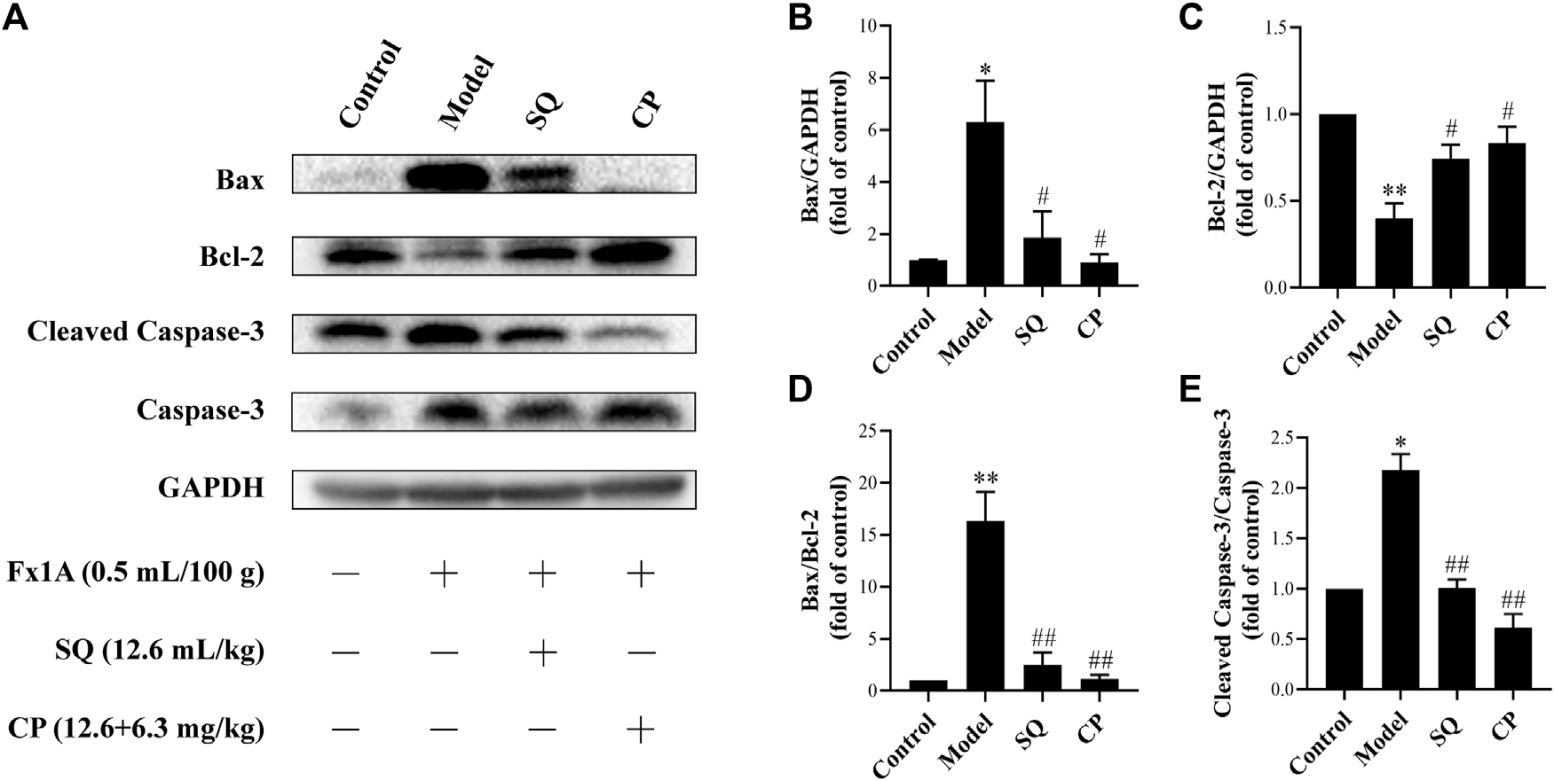
SQ diminished the activation of the apoptosis pathway in glomeruli of PHN rats. Western blot was adopted to analyze apoptosis protein markers in kidney of rats in each group **(A)**, including Bax, Bcl-2, Cleaved Caspase-3, Caspase-3. Treatment with SQ and CP could suppress the protein levels of Bax **(B)** and Cleaved Caspase-3 **(E)** and elevate the expression of Bcl-2 **(C)** in kidney of PHN rats together with the Bax/Bcl-2 ratio **(D)** (*n* = 3). Data are represented as mean ± SD from independent groups. ^*^ *p* < 0.05 vs. control group. ^**^ *p* < 0.01 vs. Control group. ^#^ *p* < 0.01 vs. Model group. ^##^ *p* < 0.01 vs. Model group.

Results of observation after TUNEL staining (Figure 4) showed that glomerular podocyte apoptosis exhibited in all groups. There was pronounced apoptosis of glomerular podocytes of PHN rats (*p* < 0.01), while, the accumulation of apoptotic podocytes of glomerulus was remarkedly inhibited with treatment of SQ and CP (*p* < 0.01). We further investigated apoptosis-related protein expressions of Bax, Bcl-2, Cleaved Caspase-3, Caspase-3 in kidney tissues of rats from all groups (Figure 5). Results of the western blot analysis showed that the apoptosis pathway was activated in PHN rats. However, SQ and CP administration significantly inhibited the activated apoptosis pathway by suppressing the elevation of protein levels of Bax together with Cleaved Caspase-3 and the reduction of Bcl-2 expression (*p* < 0.05 or *p* < 0.01).

ADR is a known injurious stimuli causing proteinuria, and the podocyte apoptosis induced by ADR is one of the main mechanisms of podocyte injury (Ni et al., 2018). In this study, our data indicated that 400 ng/ml ADR caused significant rise in Cleaved Caspase-3 protein expression (Figure 7), while SQL was given to ADR-treated podocyte and markedly reduced Cleaved Caspase-3 protein expression (*p* < 0.05).

The above results suggested that SQ could ameliorate podocyte injury in glomerulus of PHN rats by reducing apoptosis, and CP can exert better anti-apoptotic effect than SQ.

### Activated Nrf2/HO-1 Pathway by SQ Lessened Podocyte Apoptosis

**FIGURE 6:**
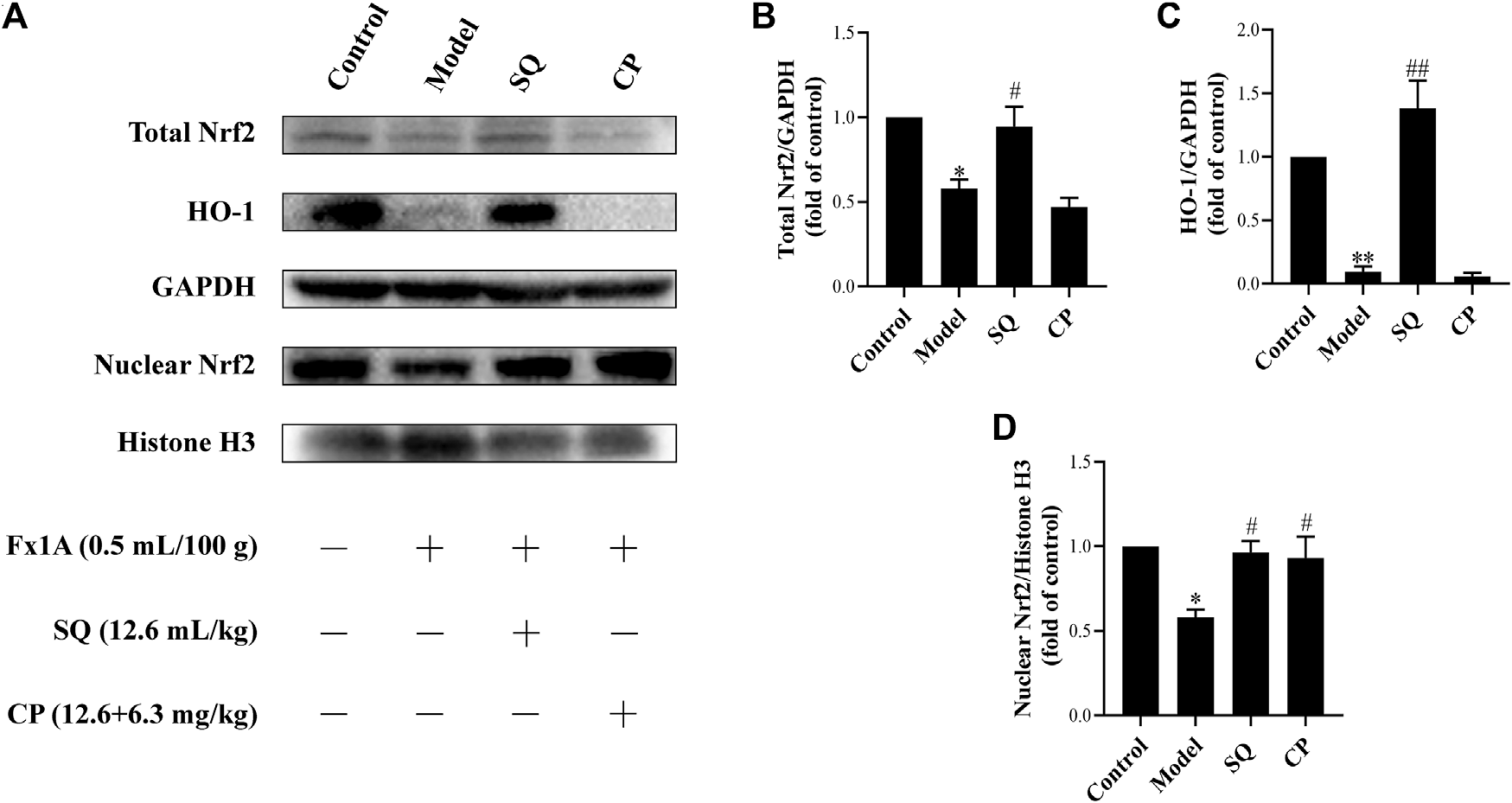
SQ reversed the repression of the Nrf2/HO-1 pathway in glomeruli of PHN rats. The protein levels of total Nrf2, nuclear Nrf2, and HO-1 in each group of the rat kidneys were detected by Western blot **(A)**. SQ intervention increased the protein expressions of total Nrf2 **(B)**, HO-1 **(C)**, and nuclear Nrf2 **(D)** in PHN rats (*n* = 3). Data are represented as mean ± SD from independent groups. ^*^ *p* < 0.05 vs. Control group. ^**^ *p* < 0.01 vs. Control group. ^#^ *p* < 0.01 vs. Model group. ^##^ *p* < 0.01 vs. Model group.

**FIGURE 7:**
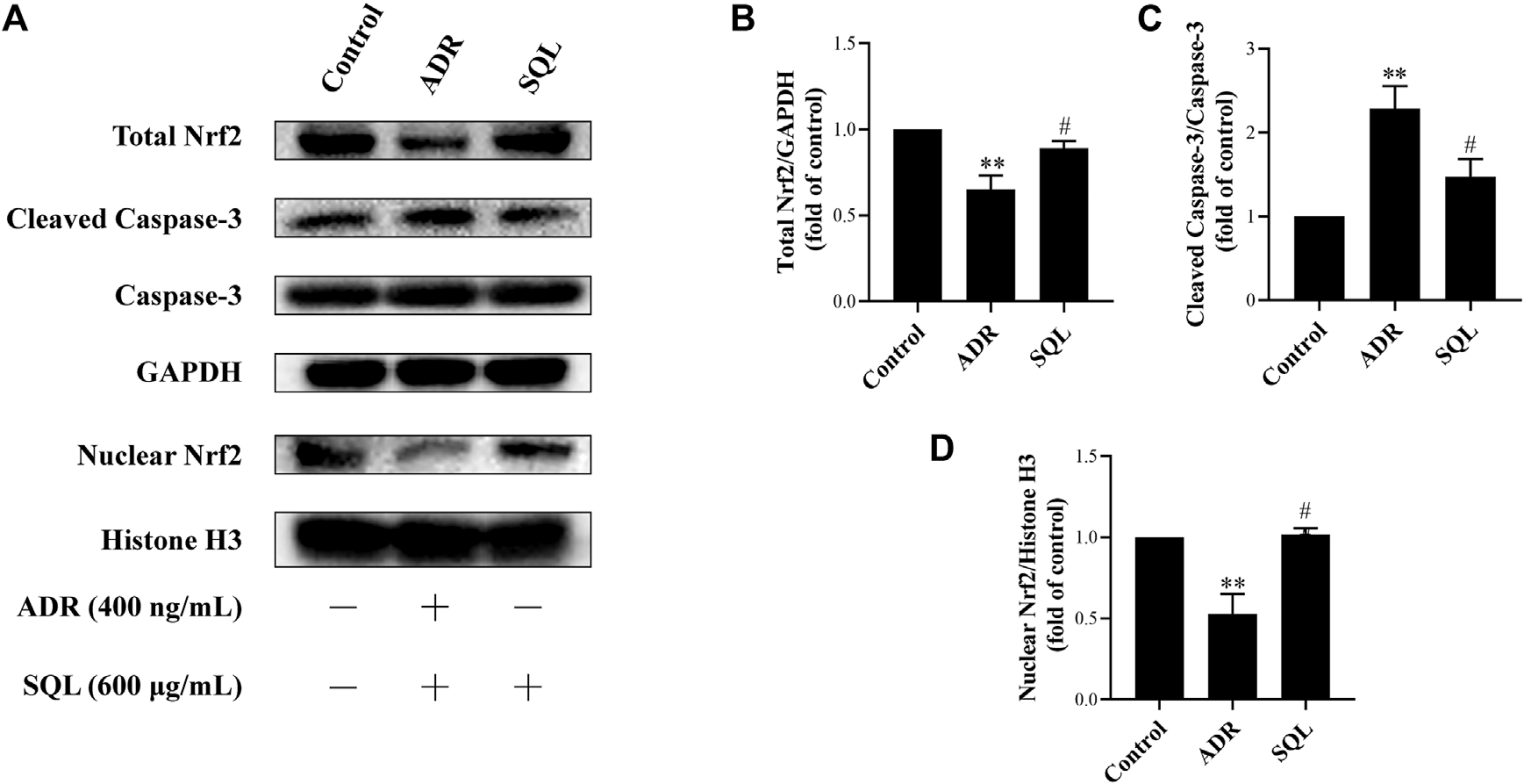
SQL suppressed ADR-injured podocyte apoptosis through activating Nrf2. The protein expressions of total Nrf2, nuclear Nrf2, and Cleaved Caspase-3 were checked by Western blot **(A)**. SQL treatment induced Nrf2 increase **(B)** and translocation in nucleus **(D)**, and then reducing podocyte apoptosis **(C)** (*n* = 3). Data are represented as mean ± SD from independent groups. ^**^ *p* < 0.01 vs. Control group. ^#^ *p* < 0.05 vs. ADR group. ^##^ *p* < 0.01 vs. ADR group.

**FIGURE 8:**
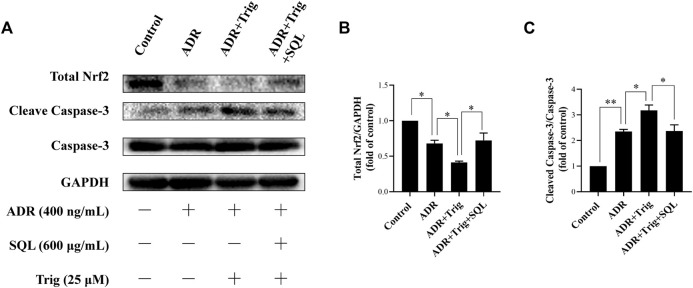
Role of Nrf2 in ADR-induced podocyte apoptosis and SQL-induced protective effect. Total Nrf2 and Cleaved Caspase-3 were measured by Western blot **(A)**. Trig abrogated lower Nrf2 expression **(B)** in ADR-injured podocyte led to higher Cleaved Caspase-3 protein expression **(C)**, while SQL cotreatment substantially increased Total Nrf2 protein expression **(B)** and reduced protein level of Cleaved Caspase-3 **(C)**. Data are represented as mean ± SD from independent groups. ^*^ *p* < 0.05. ^**^ *p* < 0.01.

Numerous studies have reported that the Nrf2/HO-1 signaling pathway is involved in modulation of apoptosis, and HO-1 can directly inhibit podocyte apoptosis. So, we wished to detect the expressions of Nrf2 and HO-1 proteins in kidneys of PHN rats and ADR-treated podocyte cells. As indicated in Figure 6, there was a prominent suppression of HO-1 in PHN rat kidneys, which was elevated with SQ intervention (*p* < 0.01). In line with HO-1 expression, decreased total protein level of Nrf2 was found in PHN rats, and SQ intervention greatly upregulated the levels of total Nrf2 proteins (*p* < 0.05). Since the key to activation of the Nrf2/HO-1 signaling pathway is the nuclear translocation of Nrf2, the impact of SQ on nuclear Nrf2 was also detected. Nuclear Nrf2 was found to be suppressed in kidneys of PHN rats, and recovered with SQ and CP intervention (*p* < 0.05). Nrf2 protein levels in podocyte cells were further checked to confirm the effect of SQ on Nrf2 (Figure 7). The total Nrf2 expression recovered with SQL treatment in presence of ADR (*p* < 0.05), and ADR restricted the Nrf2 nuclear translocation, while SQL did the opposite (*p* < 0.01). To determine whether SQ stimulated Nrf2 activity exert leading role in mitigating podocyte apoptosis, an effective inhibitor of Nrf2, Trig, was added to podocyte. As shown in Figure 8, abrogated Nrf2 expression induced by Trig in ADR-injured podocyte led to higher Cleaved Caspase-3 protein expression (*p* < 0.05). However, SQL cotreatment substantially suppressed Nrf2 reduction and Cleaved Caspase-3 rise (*p* < 0.05). In a nutshell, SQ exerted therapeutic effect on PHN rats by mitigating podocyte apoptosis and proteinuria *via* the Nrf2/HO-1 signaling pathway.

## Discussion

IMN remains the most general form of the nephrotic syndrome and one of the frequent causes of end-stage kidney disease in adults (Cattran and Brenchley, 2017). The fact that a third of IMN patients will achieve spontaneous remission and the principle of primum non nocere (first, do no harm) has led to the suggestion that IST and other toxic therapeutic regimens should be limited to patients (Hofstra et al., 2013). The need for more promising remedies to promote treatment strategies in early stage of IMN is urgent. In this study, we identified and validated SQ, a formulated preparation with good clinical effect on IMN, for excellent repression on engendering of proteinuria and the protective effect on podocyte in PHN rats and ADR-induced apoptotic podocytes, and in-depth explorations shown here proposed that SQ could reduce podocyte apoptosis *via* the Nrf2/HO-1 signaling pathway.

To evaluate the renoprotective effects of SQ on IMN, a classical PHN rat model was employed as the experimental model that is marked by IgG, C3, and C5b-9 depositions on the subepithelial aspect of the glomerular capillary loops, together with the pachynsis of GBM and a severe proteinuria (Sugisaki et al., 1973). The PHN model is widely used as its relative stability and similar pathological changes to human membranous nephropathy at both the clinical and histological levels since 1973 (Beck and Salant, 2014). An injection of rabbit antiserum against Fx1A complex of the proximal convoluted tubule to rats was implemented to induce subepithelial glomerular immune deposits, the proteinuria of rats increased continuously during the experiment (Cunningham et al., 2001). Subepithelial electron-dense deposits could be detected after 3–5 days of anti-Fx1A antiserum injection, and PHN model rats developed persistent albuminuria after about 7–10 days (Jiang et al., 2020).

Preliminary clinical observations indicated that SQ performed better clinical efficacy in the low risk group and the medium risk group of IMN patients that are in the early stage of IMN course. Therefore, it was adopted to ascertain the early curative effect of SQ on IMN that we started SQ intervention in the PHN rat model at the moment of anti-Fx1A antiserum injection when proteinuria was in the establishment stage, and related biochemical indicators in each group were sensed before the peak of proteinuria in PHN rats.

It is well known that podocyte injury is the pivotal event of proteinuria after immune response in the development of IMN (Liu et al., 2017; Jin et al., 2019). As in the PHN model, the target antigen components on podocytes combine with rabbit antibody to form *in situ* immune complexes on the GBM (Zanetti and Druet, 1980). Central to the pathogenesis of IMN is the subepithelial immune complexes deposition with GBM thickening, which produce podocyte injury through complement-dependent processes (Ronco and Debiec, 2005). After the complement system is activated by immune complexes, C5b-9 assembles on the podocyte membrane resulting in podocyte injury (Ronco and Debiec, 2012). As the barrier of the glomerular filtration, podocyte injury or death led to a large amount of proteinuria secretion and the progression of IMN (Yang et al., 2021). Hence, inhibiting immune action and reducing podocyte lesion could evidently dominate suppress proteinuria of IMN. Our results showed that treatment of SQ exerted significant repression in glomerular IgG deposition, and significant difference of complement C3 and C5b-9 MAC together with GBM thickening also could be seen between the PHN and SQ treatment group correspondingly. What is more is that SQ efficiently restored the expression of synaptopodin, marker of podocyte injury. As a result, remission of proteinuria and resume of serum albumin were promoted dramatically in the SQ-treated group.

Parallel to proteinuria and the loss of podocyte injury marker, diffuse fusion of the foot process was observed under electron microscopy in PHN rats. After SQ intervention, glomerular fusion of the foot process was markedly diminished, and the normal morphology of podocytes was restored to a certain extent. The above results confirmed the renoprotective of SQ focused on local podocyte injury, which is consistent with our previous study (Tian et al., 2019).

Podocyte injury or loss is key element in the formation of albuminuria and the progression and development of IMN (Jo et al., 2021). As highly specialized and terminal differentiated cells, podocyte have very limited ability of division and proliferation (Nagata, 2016). When injured by stimulating factors including C5b-9, podocytes are prone to death and eventually abscission occurs (Zhang et al., 2021). In the condition of podocyte death, apoptosis is an important form, and following the production of proteinuria and the progress of IMN. There is accumulating evidence indicating that reducing podocyte apoptosis can strikingly relieve proteinuria of IMN (Jin et al., 2019; Sun et al., 2021), and it is found that C5b-9 assemble could lead to glomerular epithelial cell injury *via* the apoptosis pathway (Ren et al., 2008). In this study, a quantitative analysis of TUNEL staining revealed that podocyte apoptosis in PHN rats was greatly decreased with treatment of SQ.

Furthermore, we evaluate the effect of SQ on the key proteins of the apoptosis pathway as follows: Cleaved Caspase-3, Caspase-3, Bax, and Bcl-2. The process of cell apoptosis is sensitized by executioner caspase (Green and Firzgerald, 2016). As the key apoptosis executioner, the cleaved activation of Caspase-3 depends on the release of Cyto-C to a large extent. Bcl-2 and Bax in the Bcl-2 family are the most important regulatory factor in cell apoptosis, which can mediate the release of Cyto-C (Bedoui et al., 2020). Bax promotes apoptosis and Bcl-2 inhibits it, and the ratio of Bax/Bcl-2 is an important index to evaluate cell apoptosis (Reed, 2006). It is confirmed that the inhibition of Bax expression and the promotion of Bcl-2 expression can reduce podocyte apoptosis (Chen et al., 2019). In our study, we showed that SQ treatment had marked effect on decreasing protein levels of Cleaved Caspase-3 and Bax and elevating Bcl-2 level in PHN rats, meanwhile the ratio of Bax/Bcl-2 was diminished. To further confirm the anti-apoptotic effect of SQ, an ADR-injured apoptotic podocyte model was used in this study. Similarly, Cleaved Caspase-3 in podocytes is reduced by SQL invention in presence of ADR stimulation. However, the underlying mechanism by which SQ exerts inhibitory effect on podocyte apoptosis needs to be further investigated.

Emerging evidence has confirmed that the Nrf2/HO-1 pathway is widely involved in podocyte apoptosis of IMN (Sutariya et al., 2017; Di Tu et al., 2020). HO-1, one isoform of rate limiting enzymes in heme catabolism, modulates different kinds of biological processes (Wang Y. et al., 2019). It is known that HO-1 expression is regulated at the transcriptional level, and the combination of activated Nrf2 and antioxidant response element (ARE) can promote HO-1 expression (Yang et al., 2013; Zeng et al., 2013). HO-1 was found to be highly expressed to exert cytoprotective effect in kidney under the condition of IMN (Wu et al., 2012). *In vivo*, glomerular extracts from C-BSA or PHN rats showed decreased Nrf2/HO-1 compared with control rats (Liu Y. et al., 2019; Di Tu et al., 2020), and *in vitro*, Nrf2/HO-1 activation also plays a vital role in podocyte apoptosis by injurious stimulus (Fan et al., 2021). In this study, results showed that protein expression of total Nrf2 or HO-1, which mediates podocyte apoptosis, was elevated remarkably after SQ treatment *in vivo* and *in vitro*. As a key nuclear transcription factor, Nrf2 needs to be translocated into the nucleus to promote downstream protective genes (Zhu et al., 2021). Our data showed that SQ markedly enhanced nuclear Nrf2 expression *in vivo* and *in vitro*. When Nrf2 was abrogated by Trig, ADR-induced podocyte apoptosis was enhanced, and SQL treatment could reduce podocyte apoptosis by activating Nrf2, further manifesting the role of Nrf2 in podocyte apoptosis and the protective of SQ. Taken together, these results could reveal, to a certain extent, the renoprotective effect of SQ for the treatment of IMN by ameliorating proteinuria and podocyte apoptosis. Furthermore, the Nrf2/HO-1 signaling pathway mediated apoptosis is involved in mechanism of the therapeutic effect of SQ *in vivo* and *in vitro*.

## Conclusion

The present results demonstrated that SQ exerts well protective effect against PHN by reducing proteinuria, and this antiproteinuric effect is associated with reduction of apoptotic podocytes. The Nrf2/HO-1 signaling pathway is involved in the anti-apoptotic effect of SQ on podocyte. In our follow-up studies, the in-depth study on the mechanism of SQ reducing podocyte apoptosis in PHN rats targeting the Nrf2/HO-1 signaling pathway will be explored.

## Data Availability

The original contributions presented in the study are included in the article/Supplementary Material; further inquiries can be directed to the corresponding authors.

## Supplementary Material

Supplementary Figure 1 Chemical profiles of SQ. UV chromatogram of SQ at 205 nm **(A)** and 284 nm **(B)** was presented. 11 peaks were identified as follows: (1) caylcosin-7-O-β-D-glucopyranoside, (2) ononin, (3) 6aR, 11aR-3-hydroxy-9,10-dimethoxypterocarpan-3-O-β-D- glucopyranoside, (4) calycosin, (5) notoginsenoside R1, (6) isomucronulatol-7-O-β-D-glucopyranoside, (7) ginsenoside Rg1, (8) ginsenoside Re, (9) formononetin, (10) ginsenoside Rb1, and (11) ginsenoside Rd.
