# Corrigendum: Sanqi Oral Solution Mitigates Proteinuria in Rat Passive Heymann Nephritis and Blocks Podocyte Apoptosis *via* Nrf2/HO-1 Pathway

**DOI:** 10.3389/fphar.2022.905693

**Published:** 2022-04-27

**Authors:** Xiaowan Wang, Jinchu Liu, Ruimin Tian, Bidan Zheng, Chuang Li, Lihua Huang, Zhisheng Lu, Jing Zhang, Wei Mao, Bo Liu, Kun Bao, Peng Xu

**Affiliations:** ^1^ State Key Laboratory of Dampness Syndrome of Chinese Medicine, The Second Affiliated Hospital of Guangzhou University of Chinese Medicine, Guangzhou, China; ^2^ Department of Nephrology, Guangdong Provincial Hospital of Chinese Medicine, Guangzhou, China; ^3^ Guangdong Provincial Key Laboratory of Chinese Medicine for Prevention and Treatment of Refractory Chronic Diseases, Guangzhou, China; ^4^ Guangdong Provincial Academy of Chinese Medical Sciences, Guangzhou, China; ^5^ Guangzhou Key Laboratory of Chirality Research on Active Components of Traditional Chinese Medicine, Guangzhou, China

**Keywords:** Sanqi oral solution, passive Heymann nephritis, podocyte apoptosis, proteinuria, Nrf2/ HO-1 signaling pathway

In the original article, there was an error. The concentration units of Sanqi oral solution lyophilized power (SQL) and doxorubicin hydrochloride (ADR) in cell experiment were incorrectly written as “600 mg/ml and 400 μg/ml.” It should be “600 μg/ml and 400 ng/ml.”

A correction has been made to **Materials and Methods**, **Podocyte Culture and Treatment**, *Paragraph 1*:

“The conditionally immortalized temperature sensitive mouse podocyte cell line used in this study was established by Professor Peter Mundel (Medical College of Harvard University, Boston, MA, United States). Briefly, podocytes were cultured in RPMI 1640 medium with 10% fetal bovine serum at 33°C in the presence of 10 U/ml recombinant mouse interferon-γ (Sigma, St. Louis, MO, United States). For inducing differentiation, podocytes were thermoshifted to 37°C and cultured in interferon-free medium for 10–14 days. SQL and Trig were dissolved in PBS and storage solutions were stored at −20°C. Podocytes were cultured overnight before experiments and treated with 400 ng/ml ADR with or without 25 μM Nrf2 inhibitor Trig or 600 μg/ml SQL intervention for 24 h.”

In the original article, there was a mistake in the labels of Figures 7, 8 as published. The concentration unit of SQL in cell experiment should be 600 μg/ml, not 600 mg/ml. The corrected Figures 7, 8 appears below.

**FIGURE 7:**
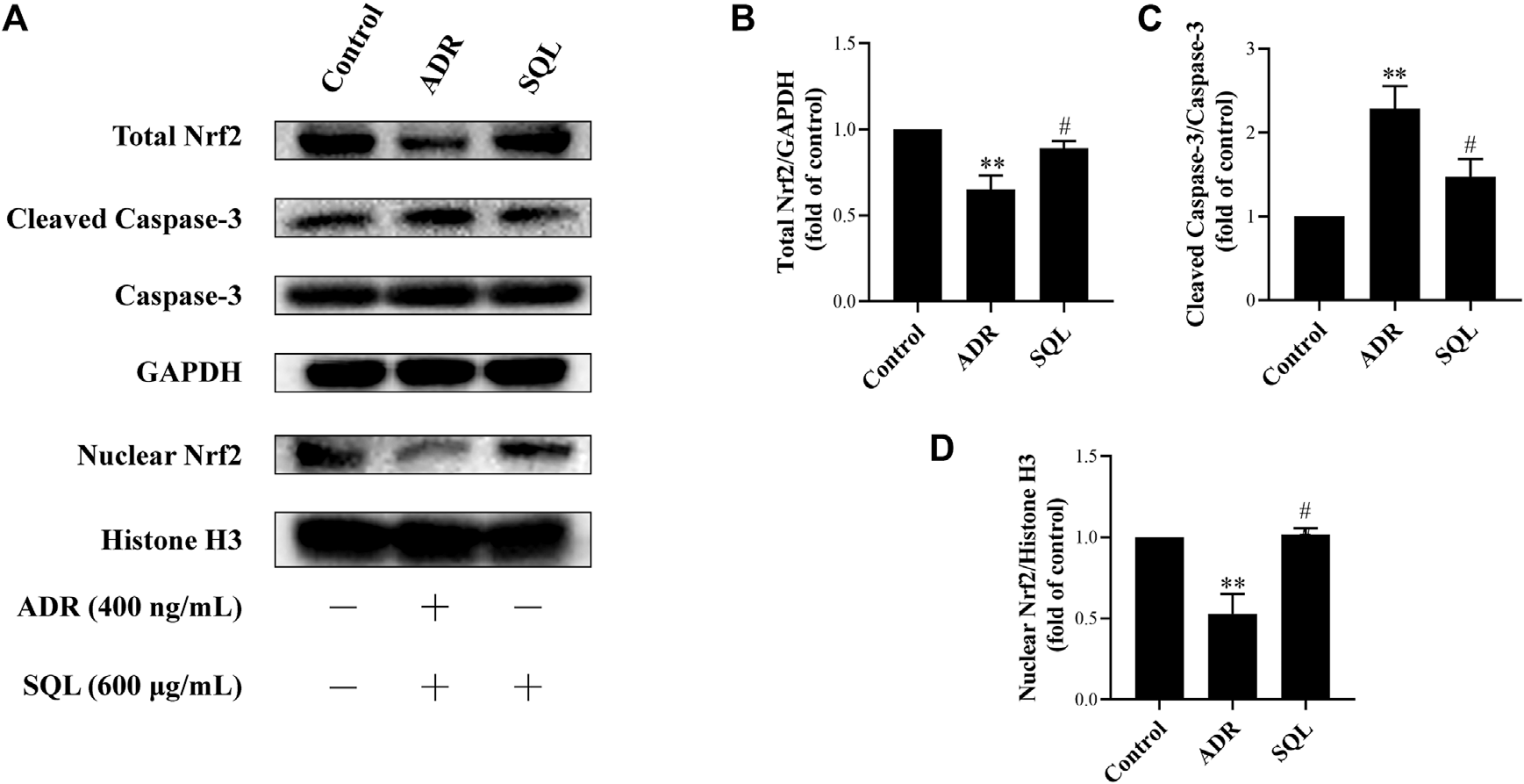
SQL suppressed ADR-injured podocyte apoptosis through activating Nrf2. The protein expressions of total Nrf2, nuclear Nrf2, and Cleaved Caspase-3 were checked by Western blot **(A)**. SQL treatment induced Nrf2 increase **(B)** and translocation in nucleus **(D)**, and then reducing podocyte apoptosis **(C)** (*n* = 3). Data are represented as mean ± SD from independent groups. **p < 0.01 vs. Control group. ^#^ *p* < 0.05 vs. ADR group. ^##^ *p* < 0.01 vs. ADR group.

**FIGURE 8:**
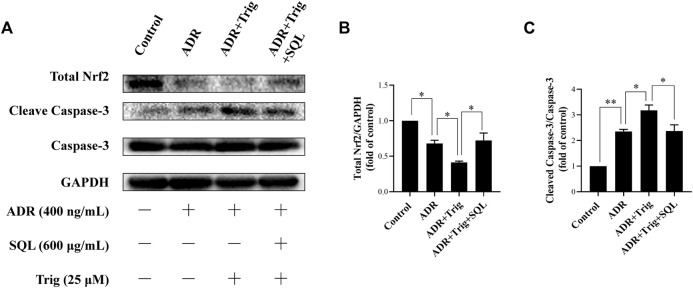
Role of Nrf2 in ADR-induced podocyte apoptosis and SQL-induced protective effect. Total Nrf2 and Cleaved Caspase-3 were measured by Western blot **(A)**. Trig abrogated lower Nrf2 expression **(B)** in ADR-injured podocyte led to higher Cleaved Caspase-3 protein expression **(C)**, while SQL cotreatment substantially increased Total Nrf2 protein expression **(B)** and reduced protein level of Cleaved Caspase-3 **(C)**. Data are represented as mean ± SD from independent groups. *p < 0.05. **p < 0.01.

The authors apologize for this error and state that this does not change the scientific conclusions of the article in any way. The original article has been updated.

