# Supplementary figures and images for: Sanqi Oral Solution Mitigates Proteinuria in Rat Passive Heymann Nephritis and Blocks Podocyte Apoptosis via Nrf2/HO-1 Pathway
Source: Front Pharmacol. 2021 Nov 19;12:727874. doi: 10.3389/fphar.2021.727874 (PMC8640486; doi:10.3389/fphar.2021.727874)

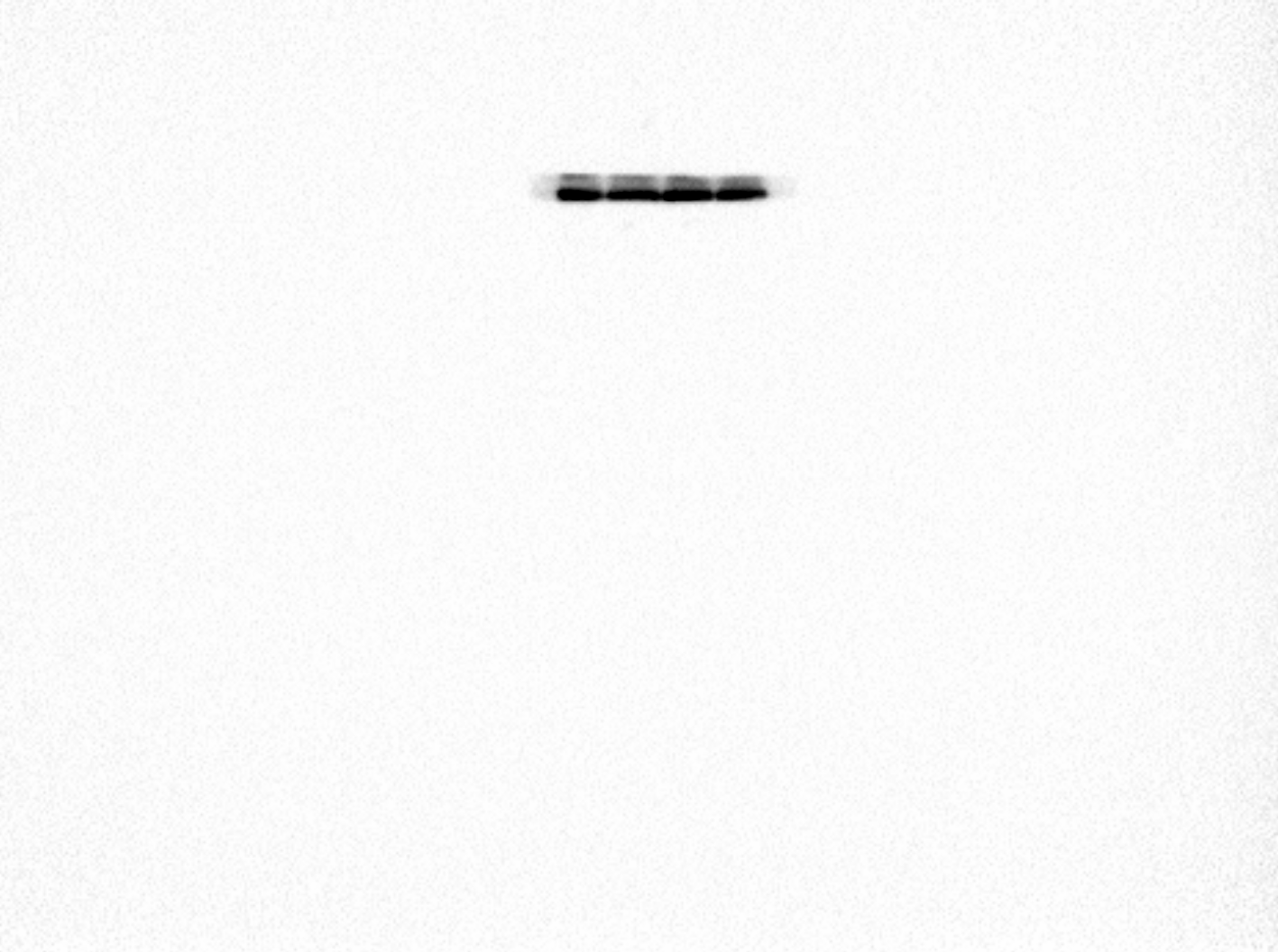

Supplement: Supplementary file 1 [file DataSheet3.zip › 5. Podocyte Total proteins+Trig WB scans/Cellular total protein-trig-c3-1.tif]

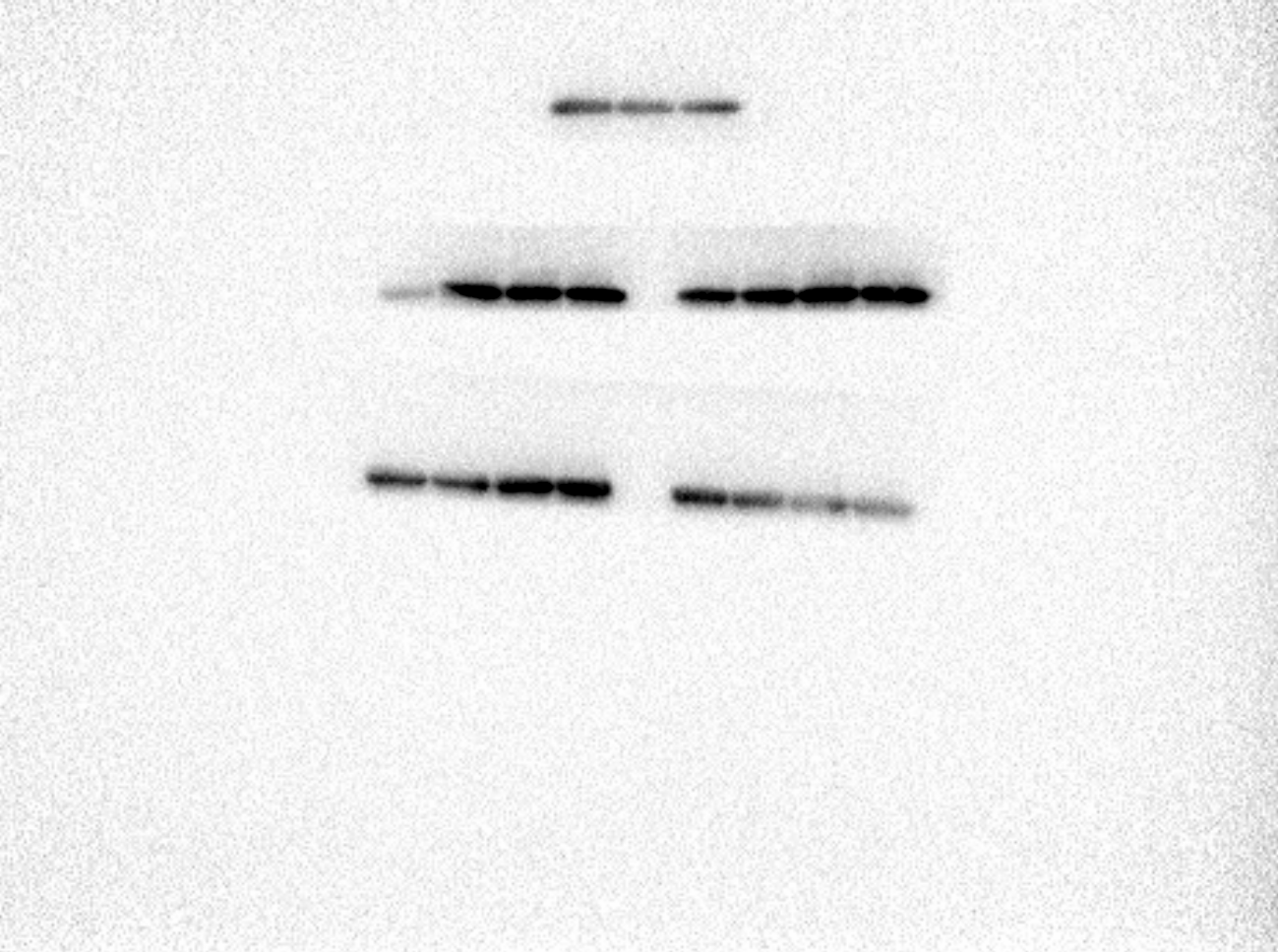

Supplement: Supplementary file 1 [file DataSheet3.zip › 5. Podocyte Total proteins+Trig WB scans/Cellular total protein-trig-c3-2.tif]

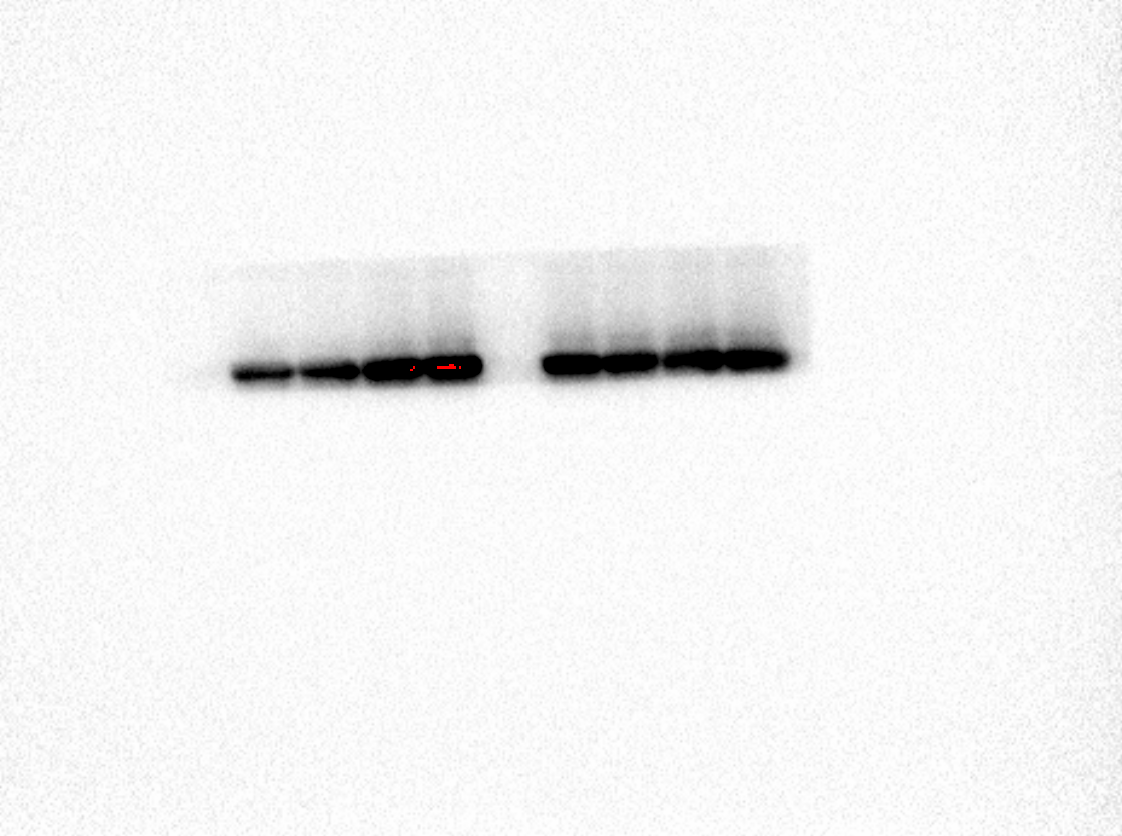

Supplement: Supplementary file 1 [file DataSheet3.zip › 5. Podocyte Total proteins+Trig WB scans/Cellular total protein-trig-c3-3.tif]

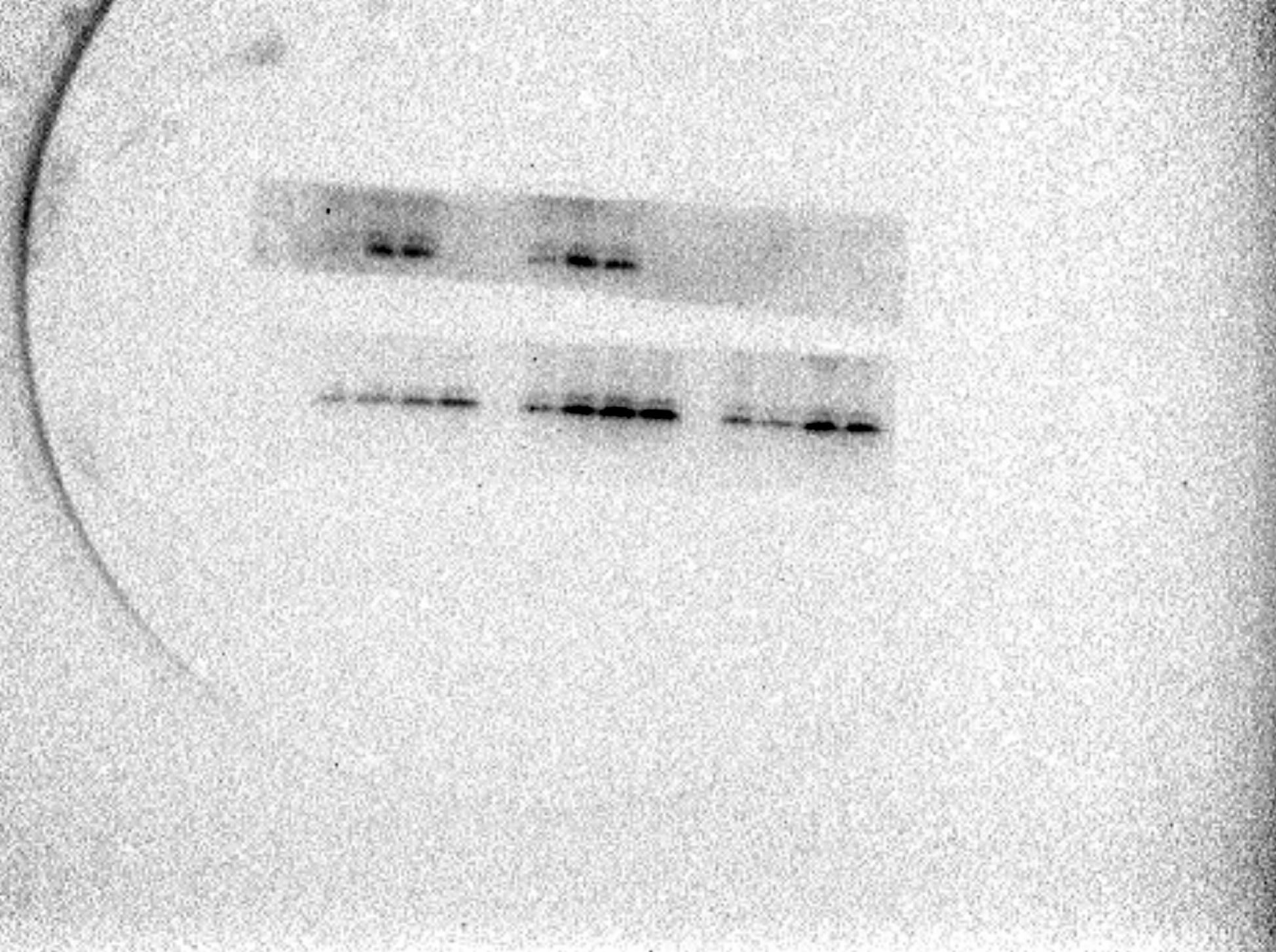

Supplement: Supplementary file 1 [file DataSheet3.zip › 5. Podocyte Total proteins+Trig WB scans/Cellular total protein-trig-cc3-1.tif]

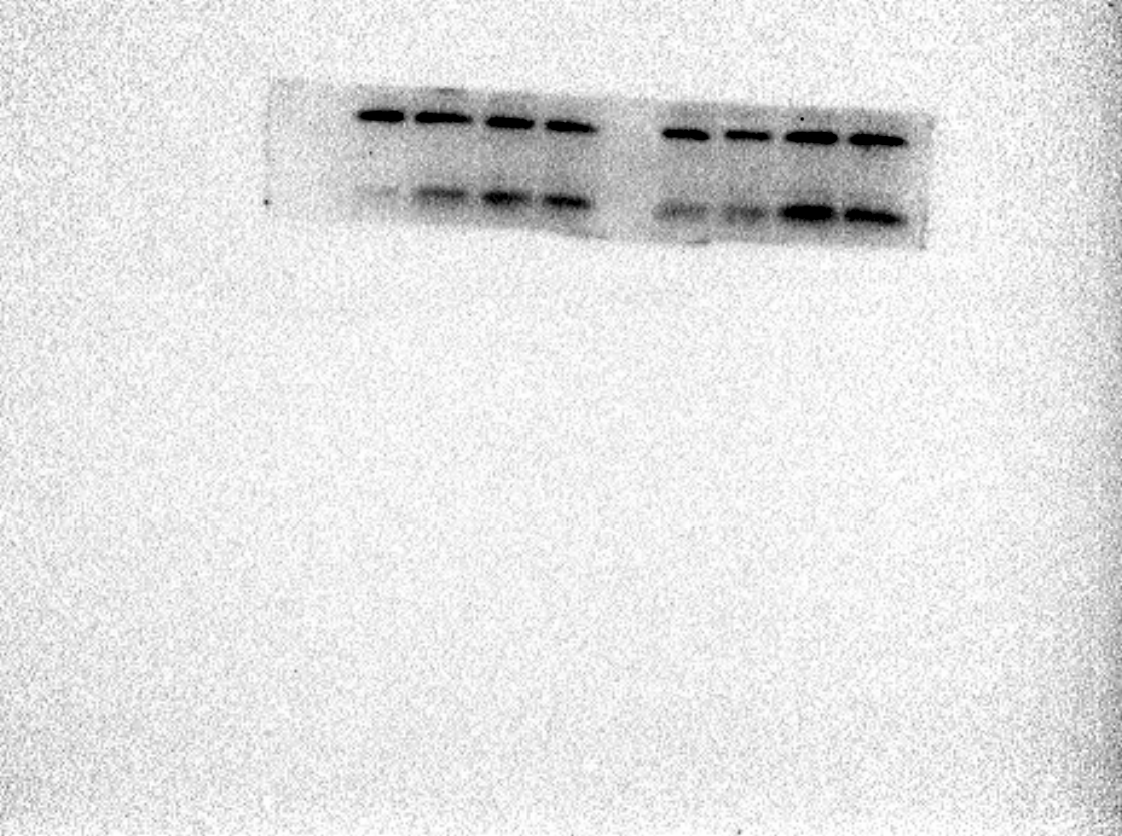

Supplement: Supplementary file 1 [file DataSheet3.zip › 5. Podocyte Total proteins+Trig WB scans/Cellular total protein-trig-cc3-2.tif]

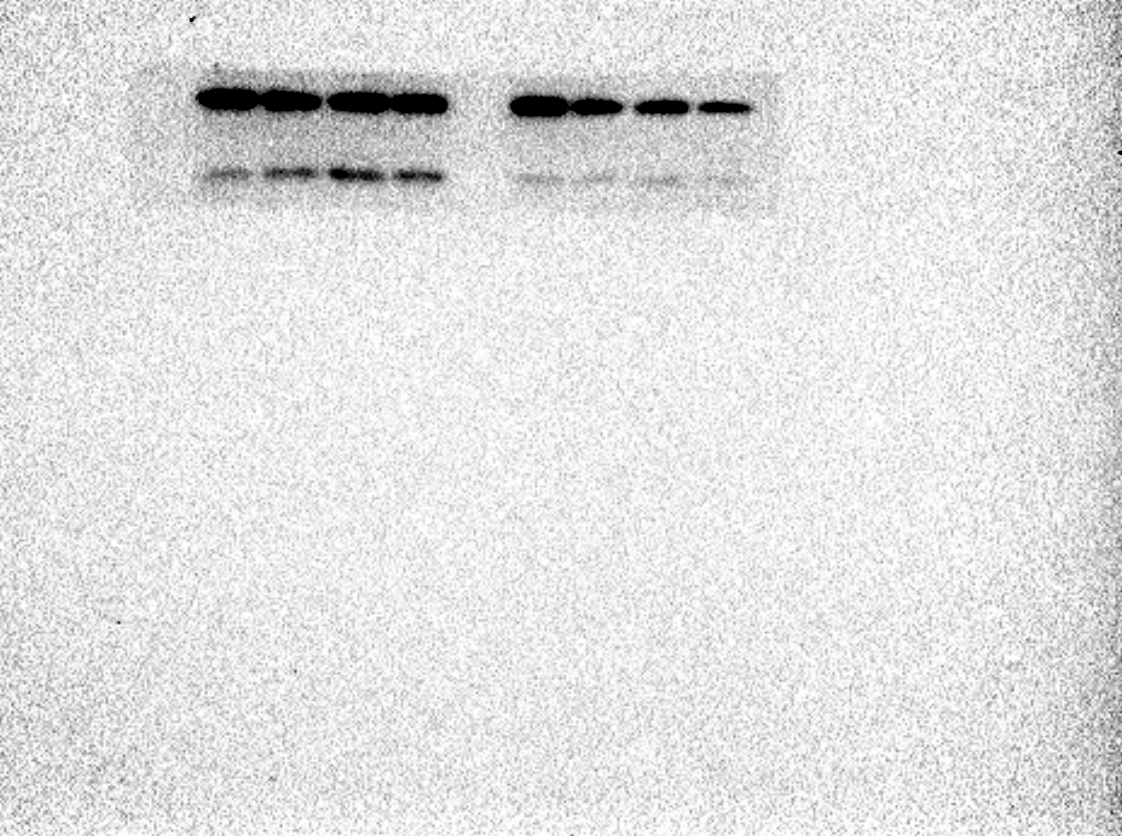

Supplement: Supplementary file 1 [file DataSheet3.zip › 5. Podocyte Total proteins+Trig WB scans/Cellular total protein-trig-cc3-3.tif]

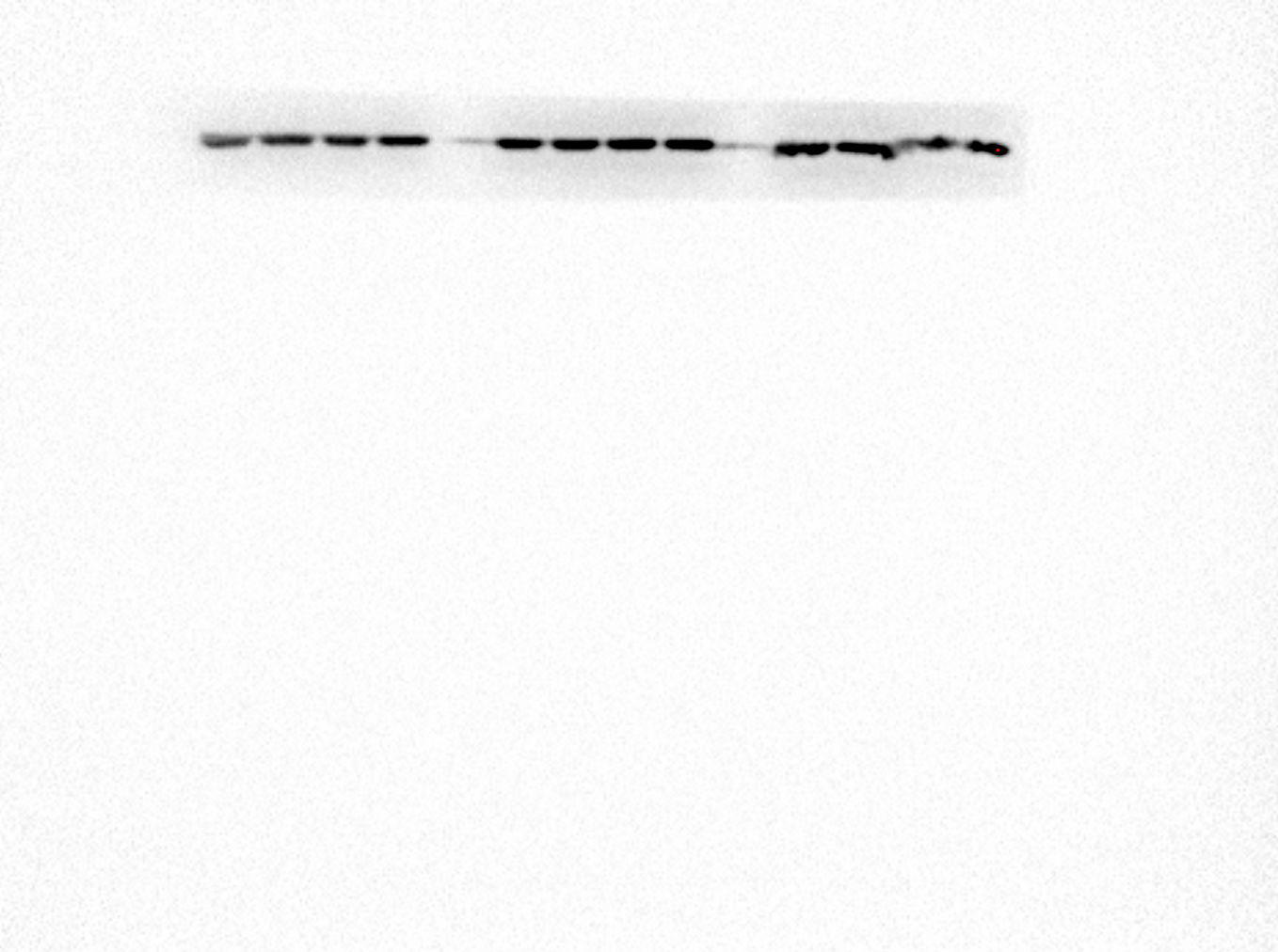

Supplement: Supplementary file 1 [file DataSheet3.zip › 5. Podocyte Total proteins+Trig WB scans/Cellular total protein-trig-gapdh-1.tif]

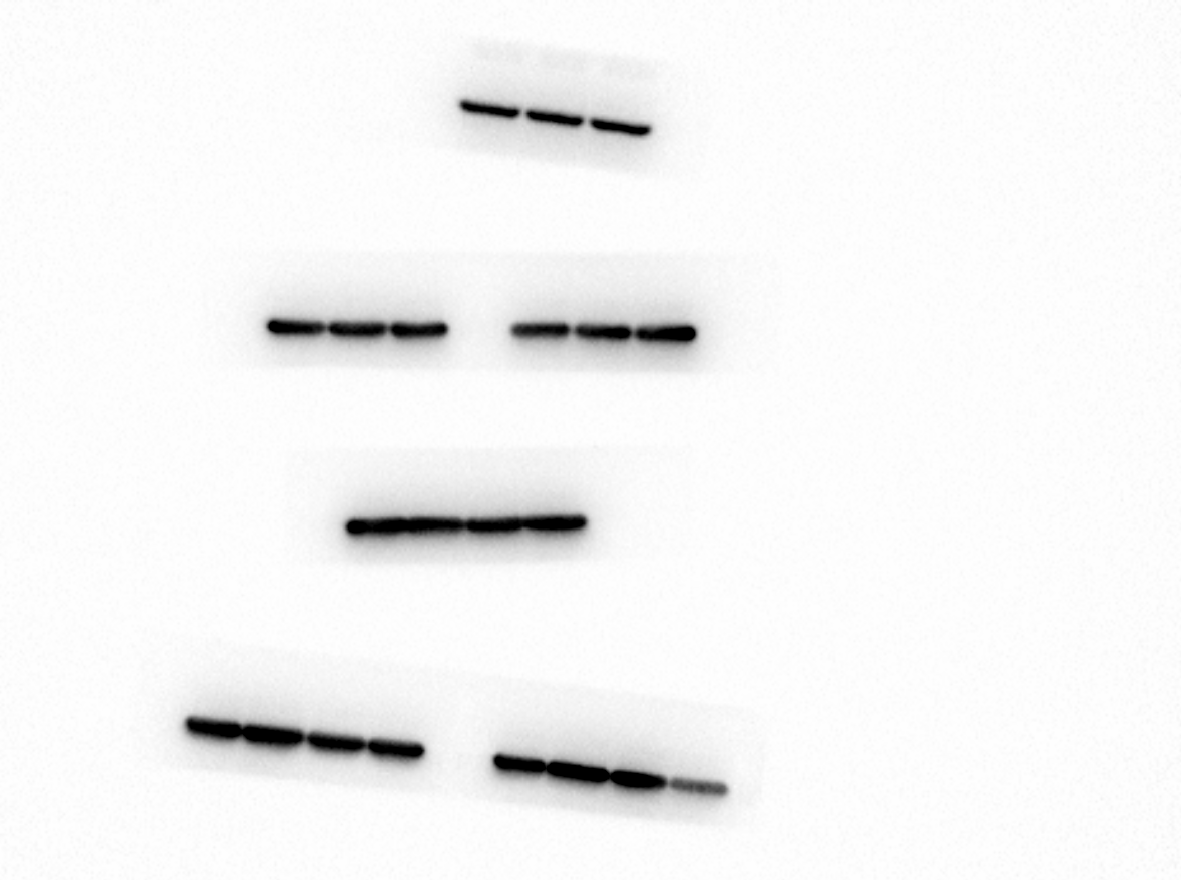

Supplement: Supplementary file 1 [file DataSheet3.zip › 5. Podocyte Total proteins+Trig WB scans/Cellular total protein-trig-gapdh-2 3.tif]

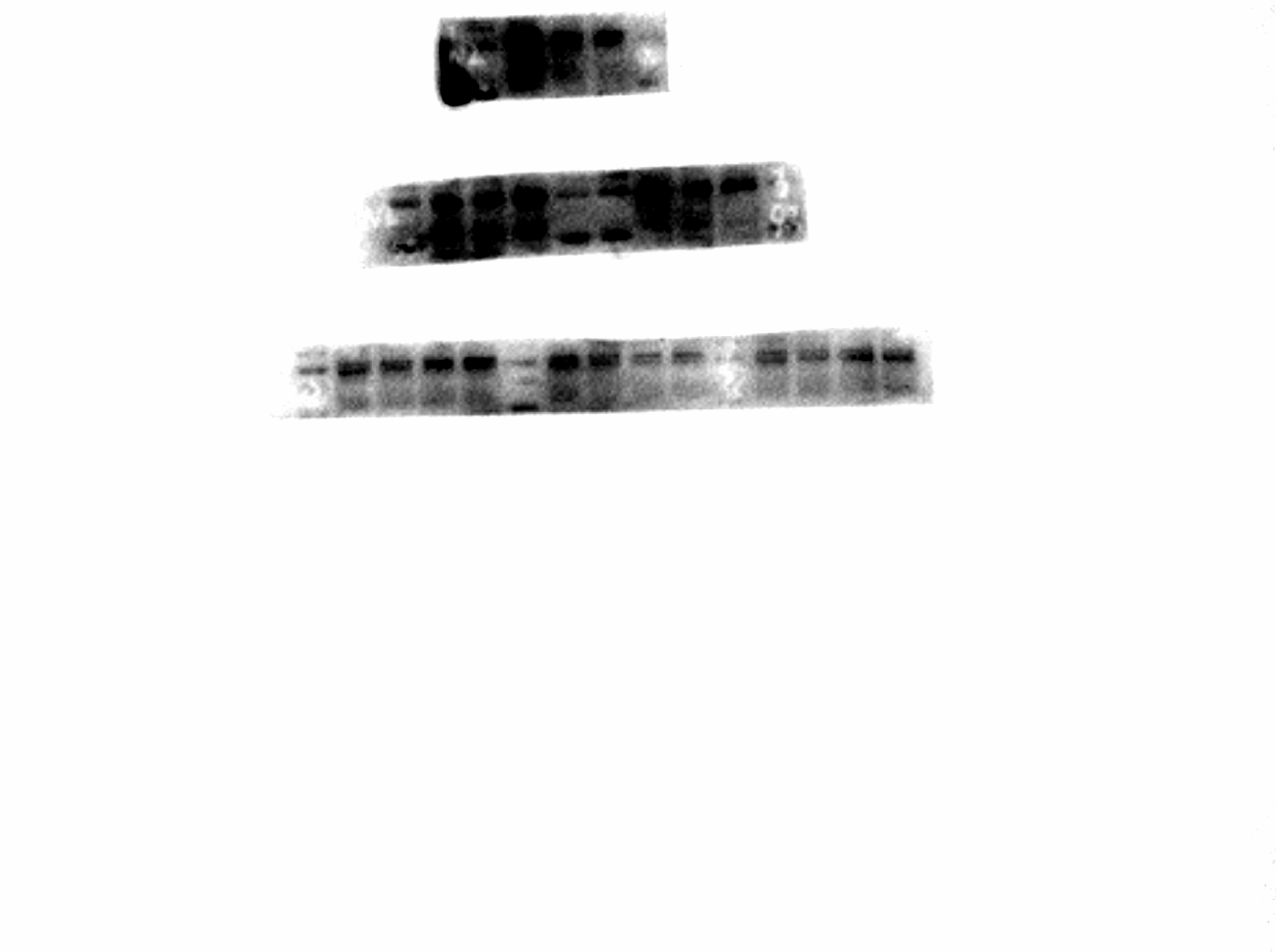

Supplement: Supplementary file 1 [file DataSheet3.zip › 5. Podocyte Total proteins+Trig WB scans/Cellular total protein-trig-nrf2-1.tif]

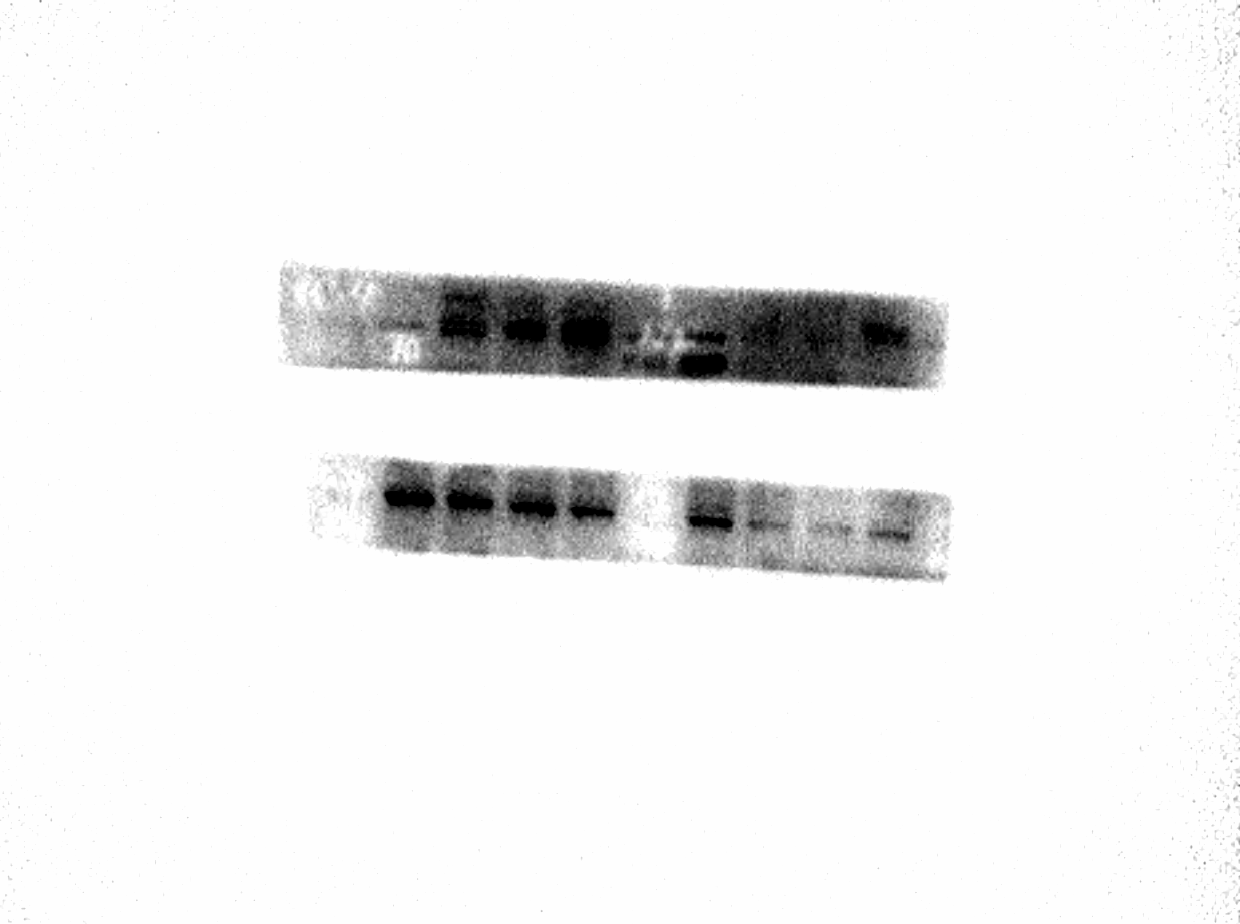

Supplement: Supplementary file 1 [file DataSheet3.zip › 5. Podocyte Total proteins+Trig WB scans/Cellular total protein-trig-nrf2-2.tif]

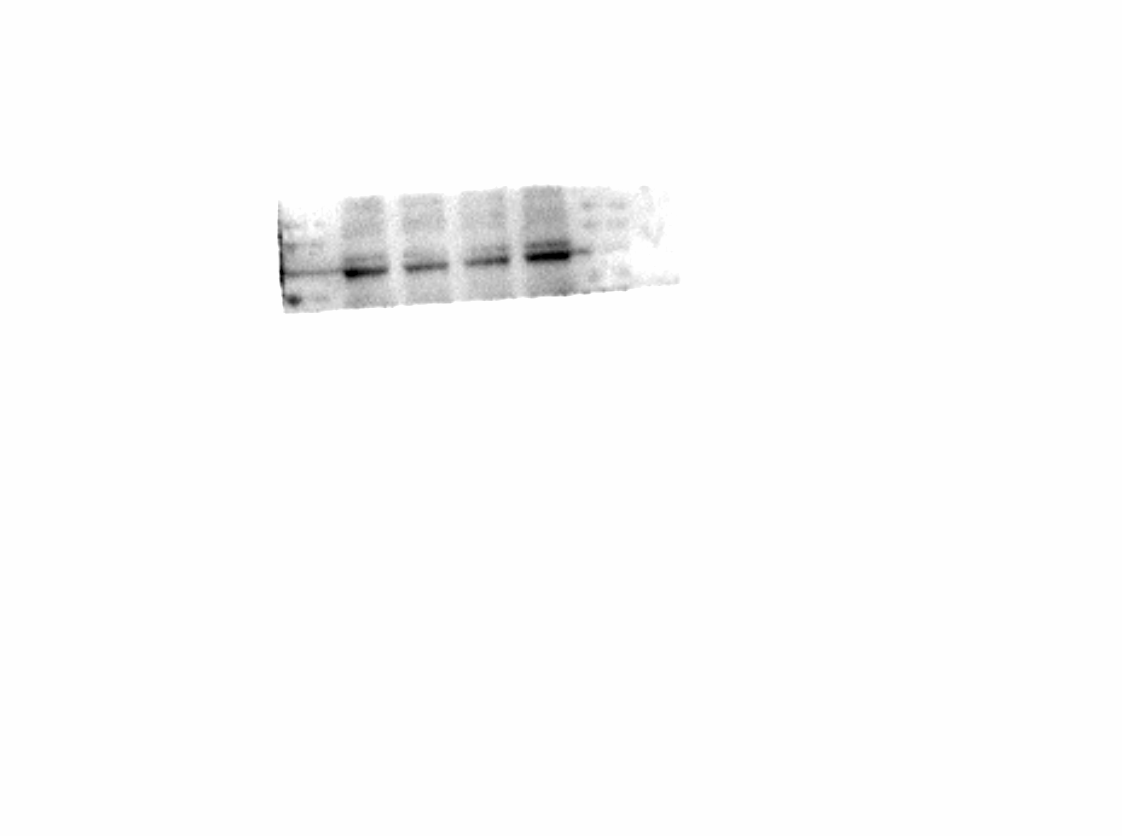

Supplement: Supplementary file 1 [file DataSheet3.zip › 5. Podocyte Total proteins+Trig WB scans/Cellular total protein-trig-nrf2-3.tif]

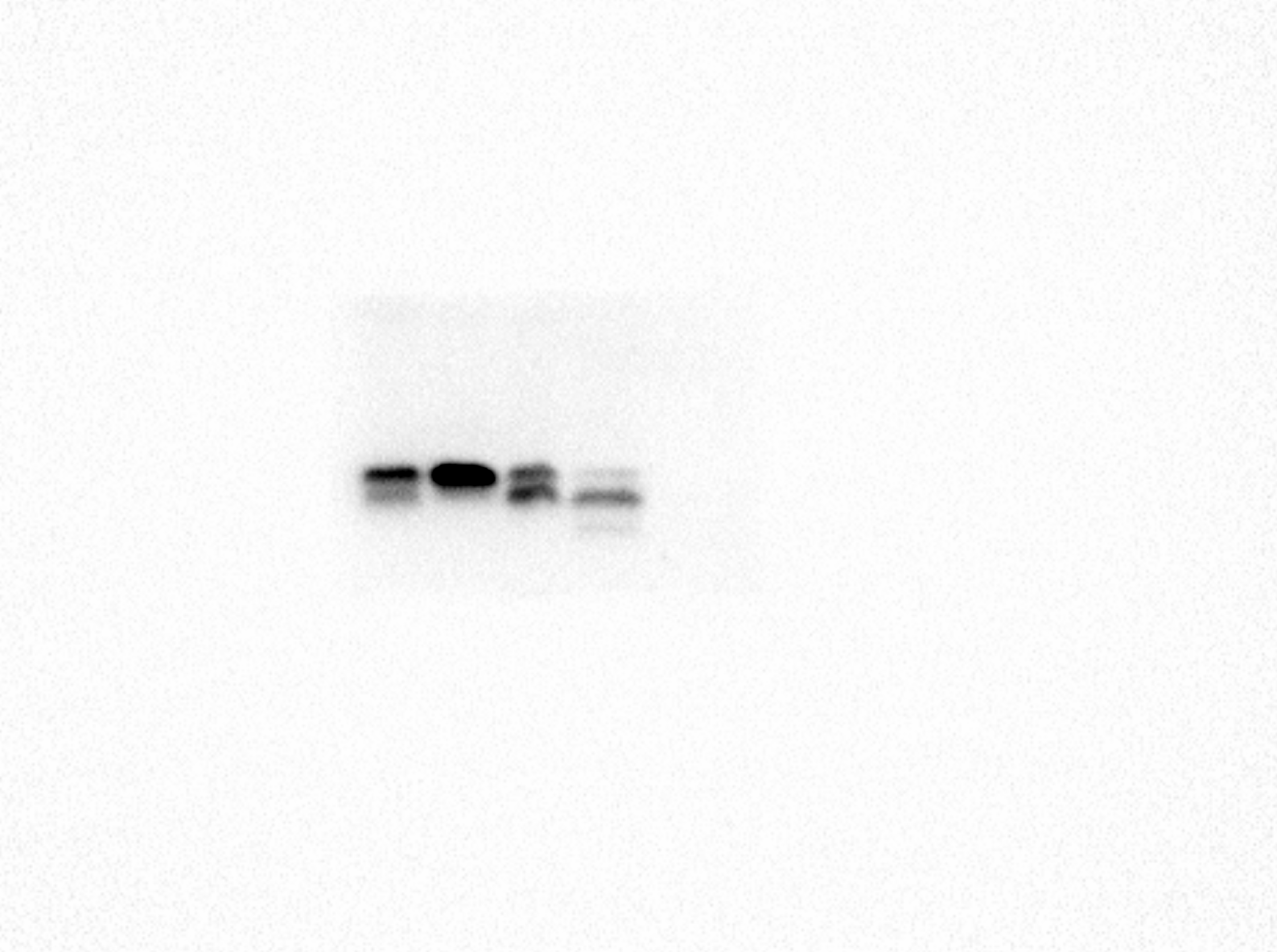

Supplement: Supplementary file 2 [file DataSheet4.ZIP › 1. Kidney Total proteins WB scans/Bax-1.tif]

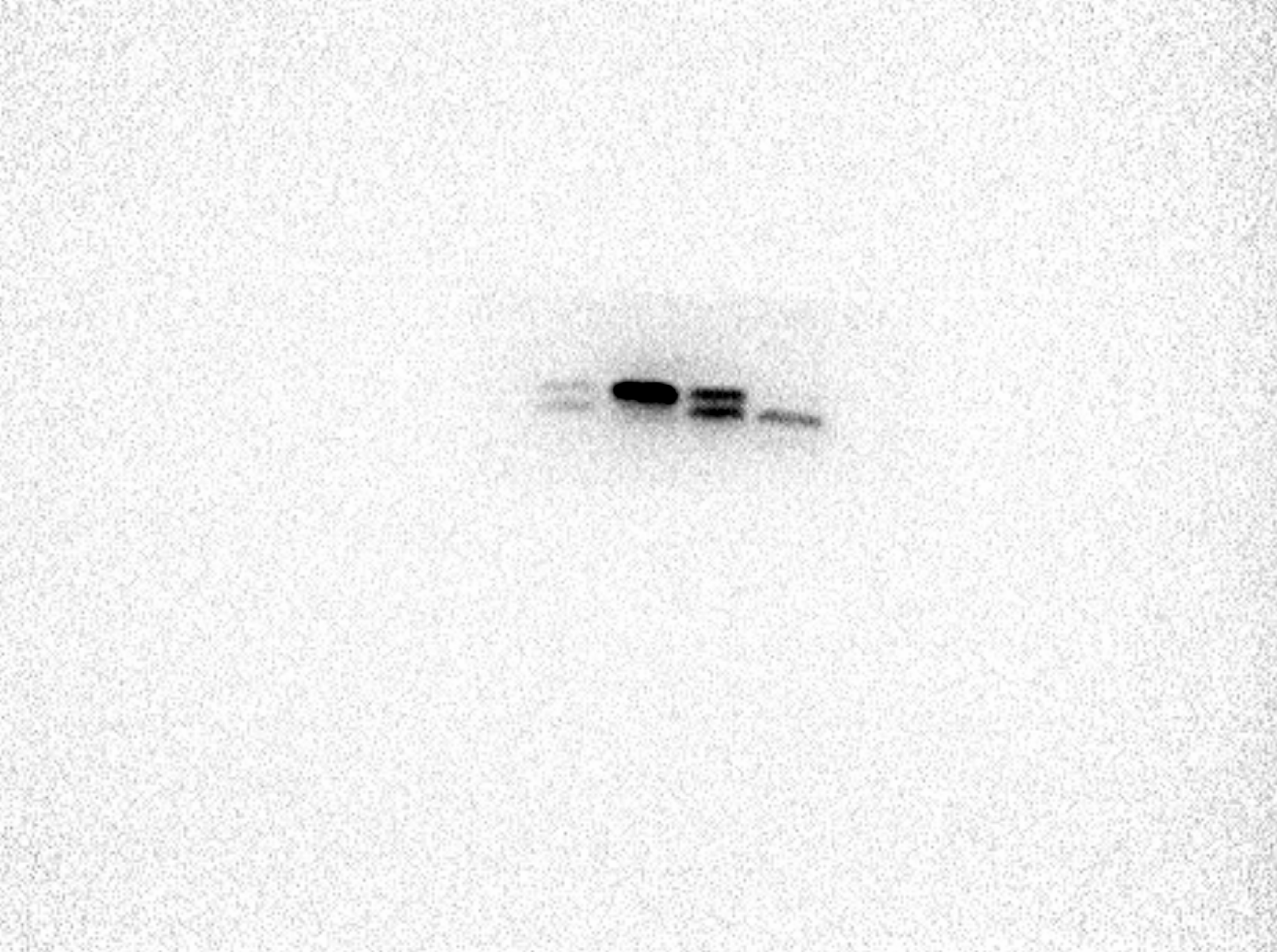

Supplement: Supplementary file 2 [file DataSheet4.ZIP › 1. Kidney Total proteins WB scans/Bax-2.tif]

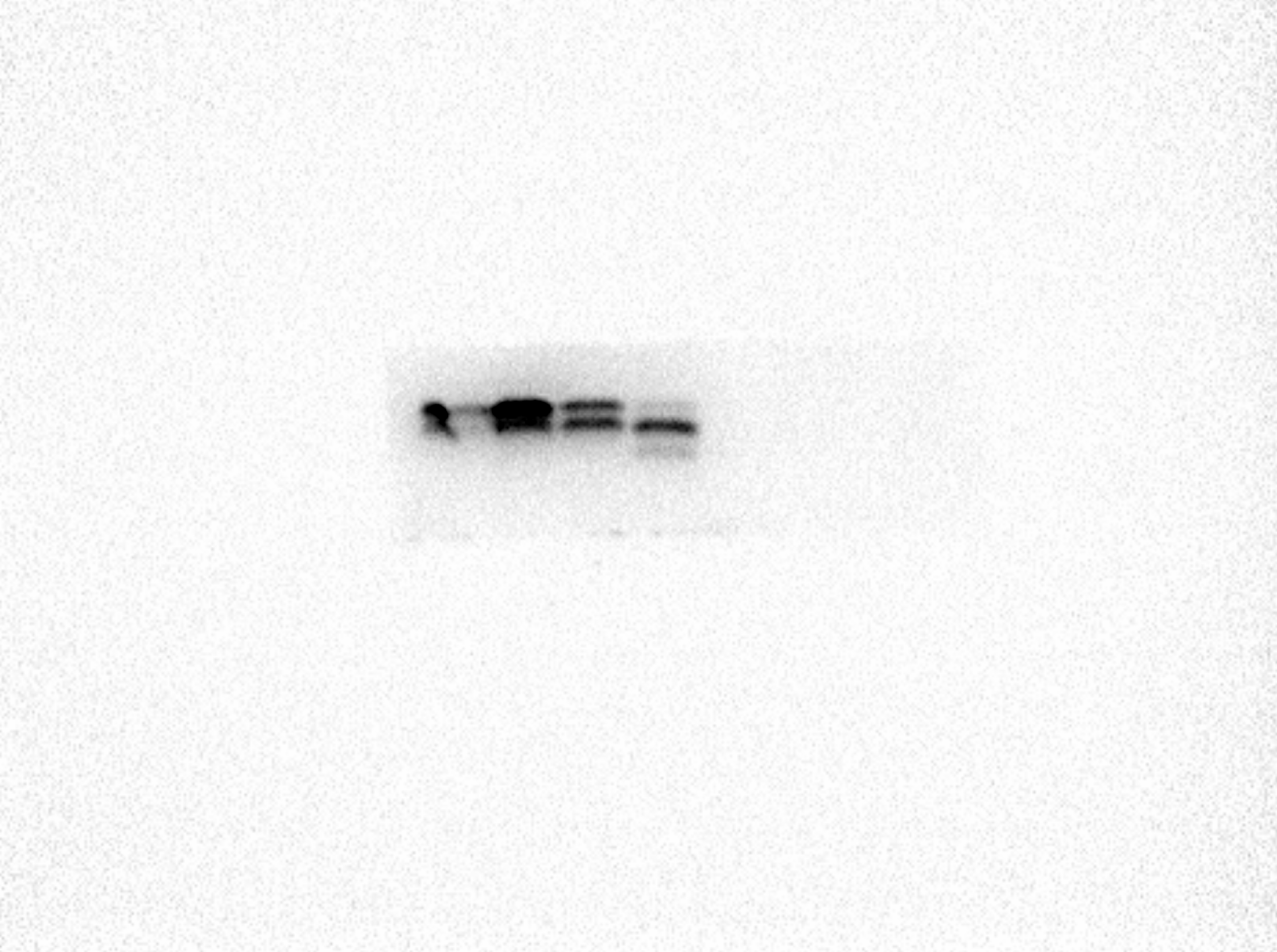

Supplement: Supplementary file 2 [file DataSheet4.ZIP › 1. Kidney Total proteins WB scans/Bax-3.tif]

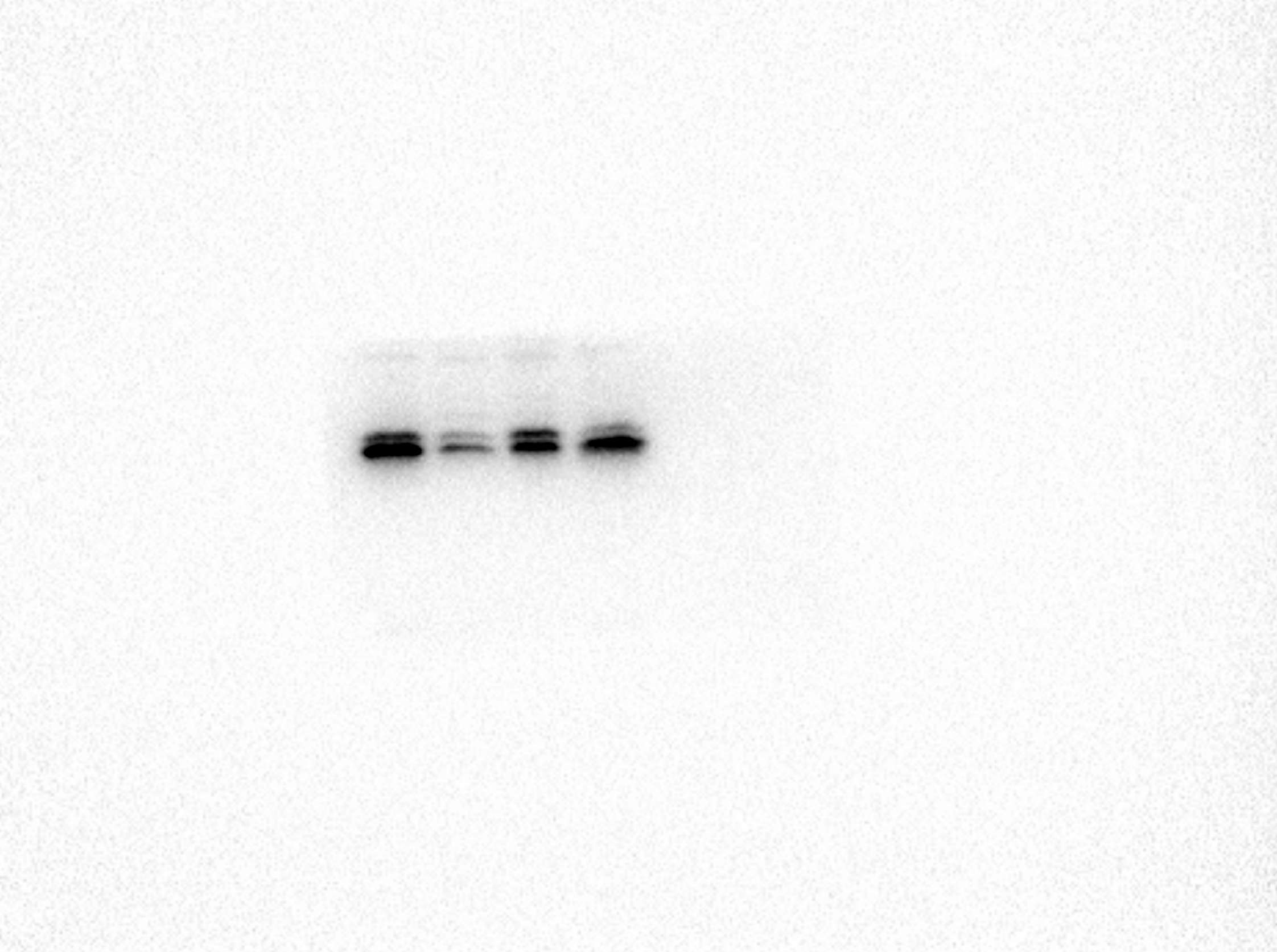

Supplement: Supplementary file 2 [file DataSheet4.ZIP › 1. Kidney Total proteins WB scans/Bcl2-1.tif]

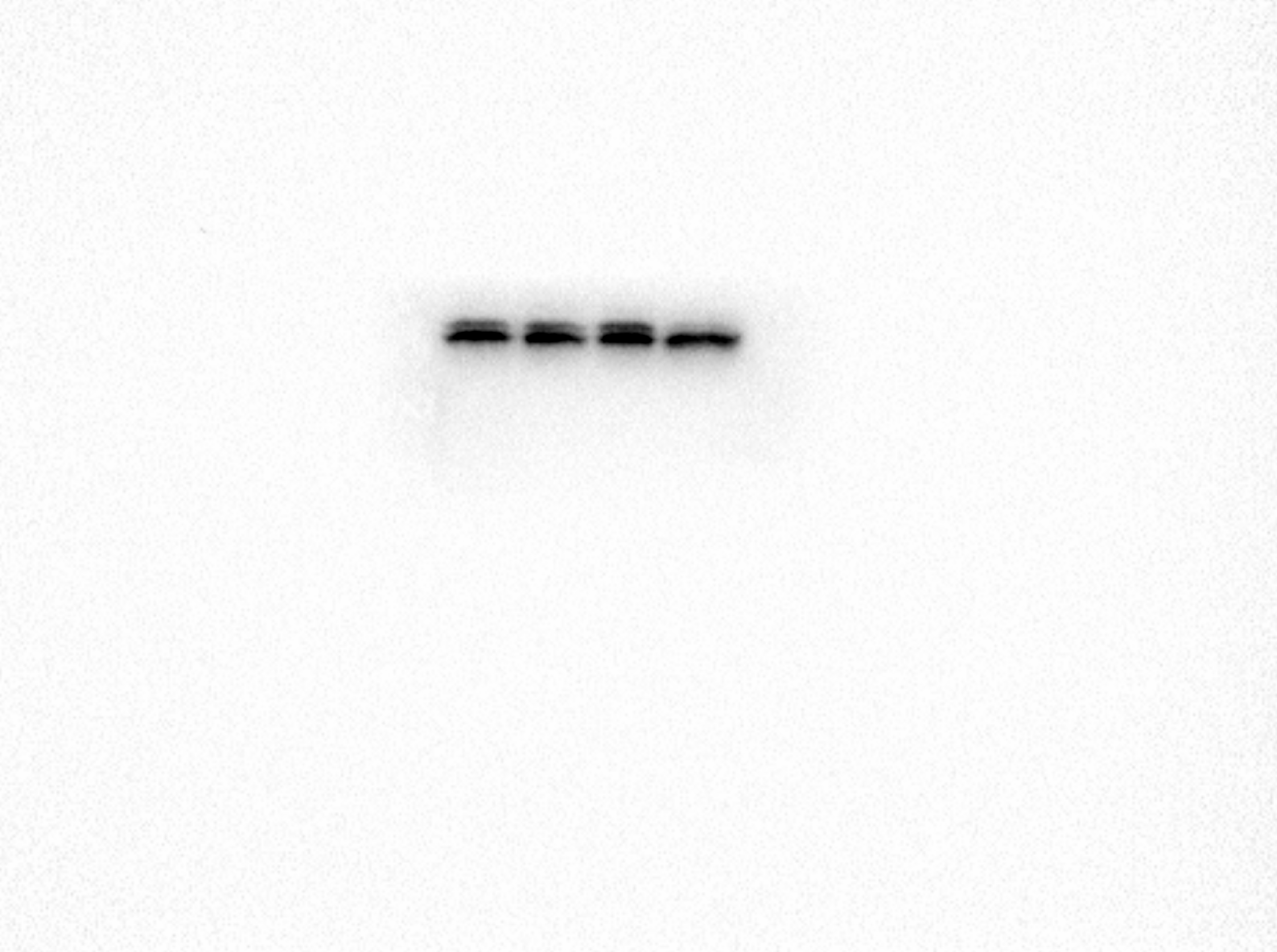

Supplement: Supplementary file 2 [file DataSheet4.ZIP › 1. Kidney Total proteins WB scans/Bcl2-2.tif]

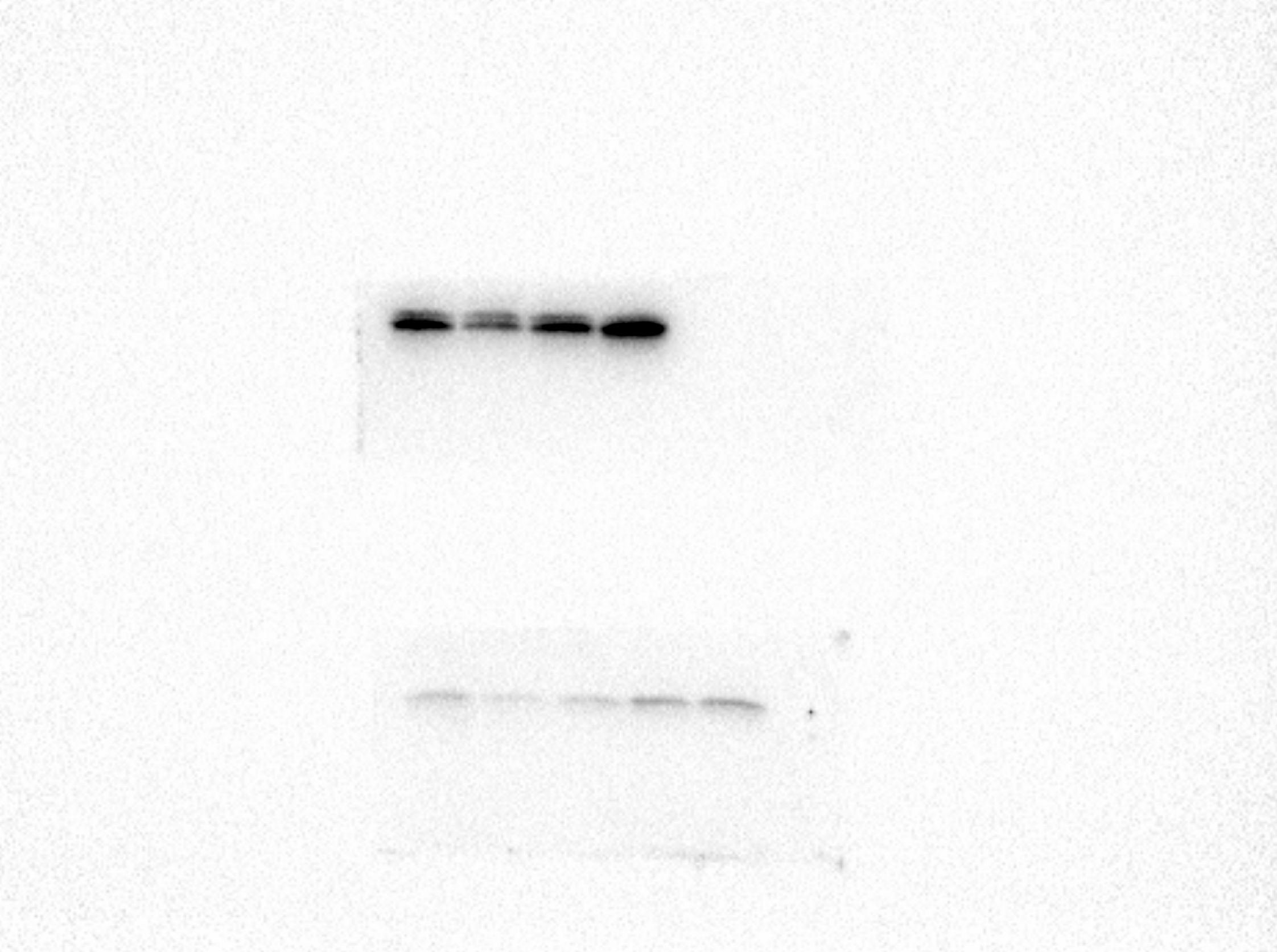

Supplement: Supplementary file 2 [file DataSheet4.ZIP › 1. Kidney Total proteins WB scans/Bcl2-3.tif]

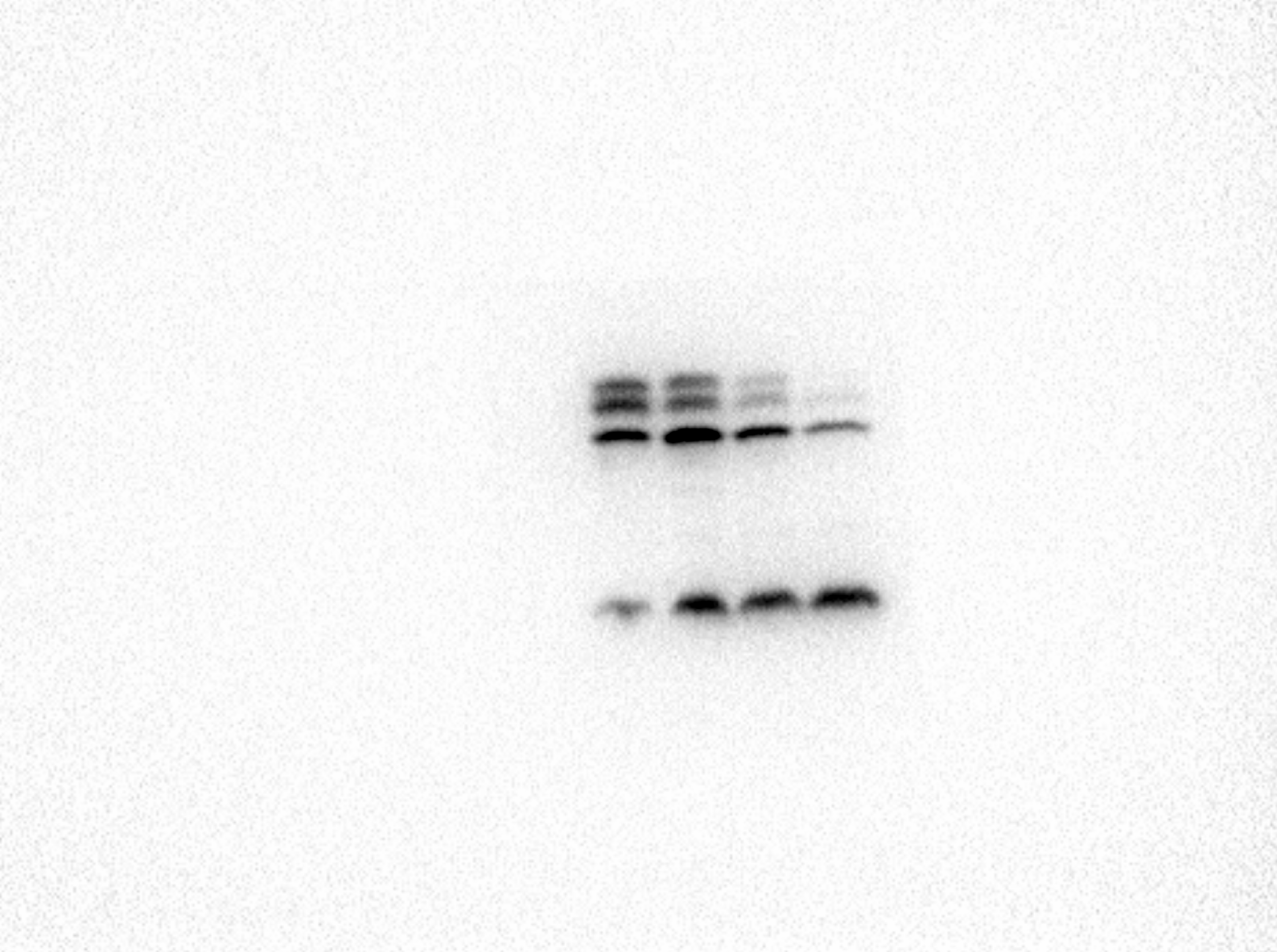

Supplement: Supplementary file 2 [file DataSheet4.ZIP › 1. Kidney Total proteins WB scans/Caspase3-1.tif]

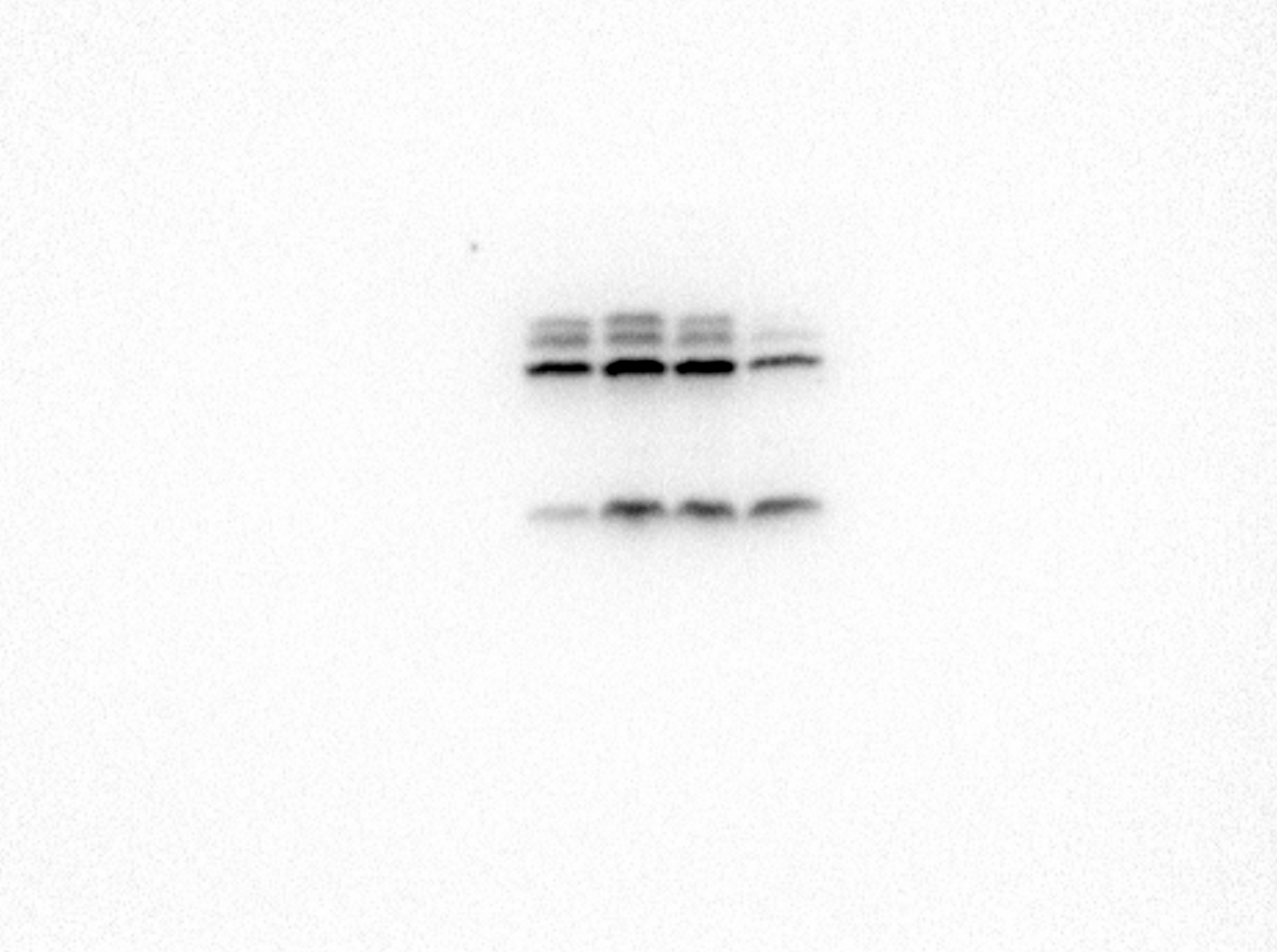

Supplement: Supplementary file 2 [file DataSheet4.ZIP › 1. Kidney Total proteins WB scans/Caspase3-2.tif]

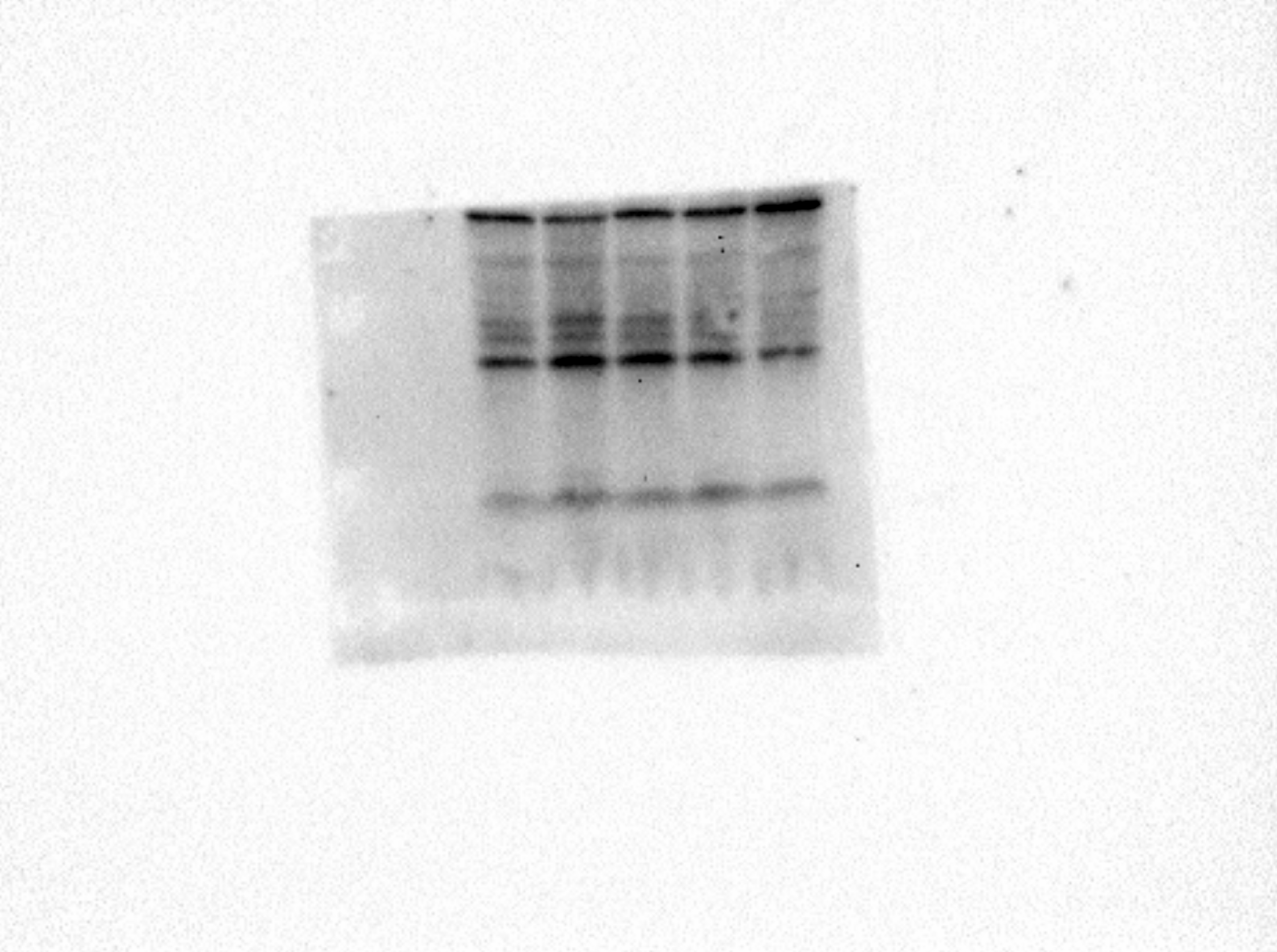

Supplement: Supplementary file 2 [file DataSheet4.ZIP › 1. Kidney Total proteins WB scans/Caspase3-3.tif]

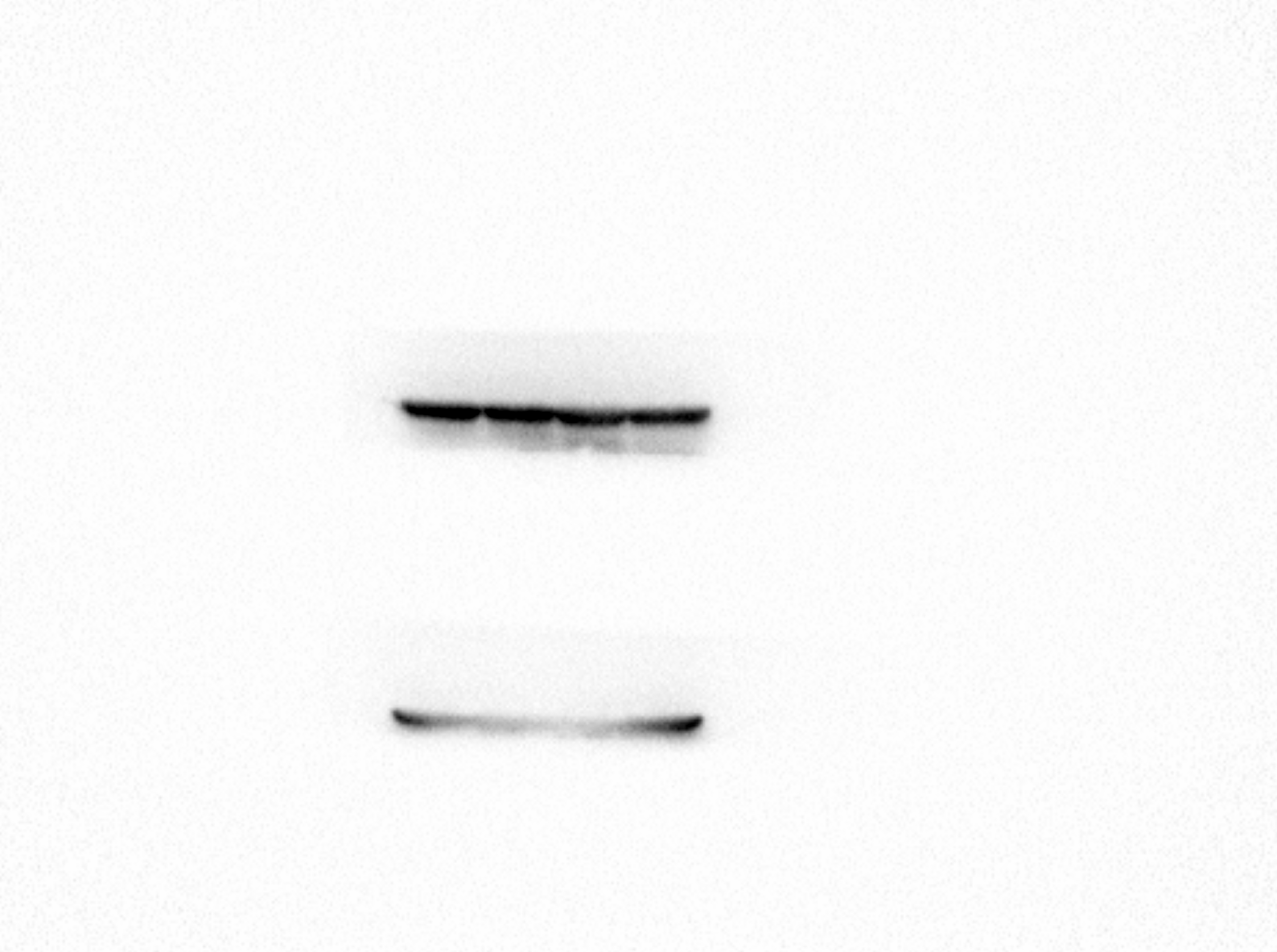

Supplement: Supplementary file 2 [file DataSheet4.ZIP › 1. Kidney Total proteins WB scans/GAPDH-1.tif]

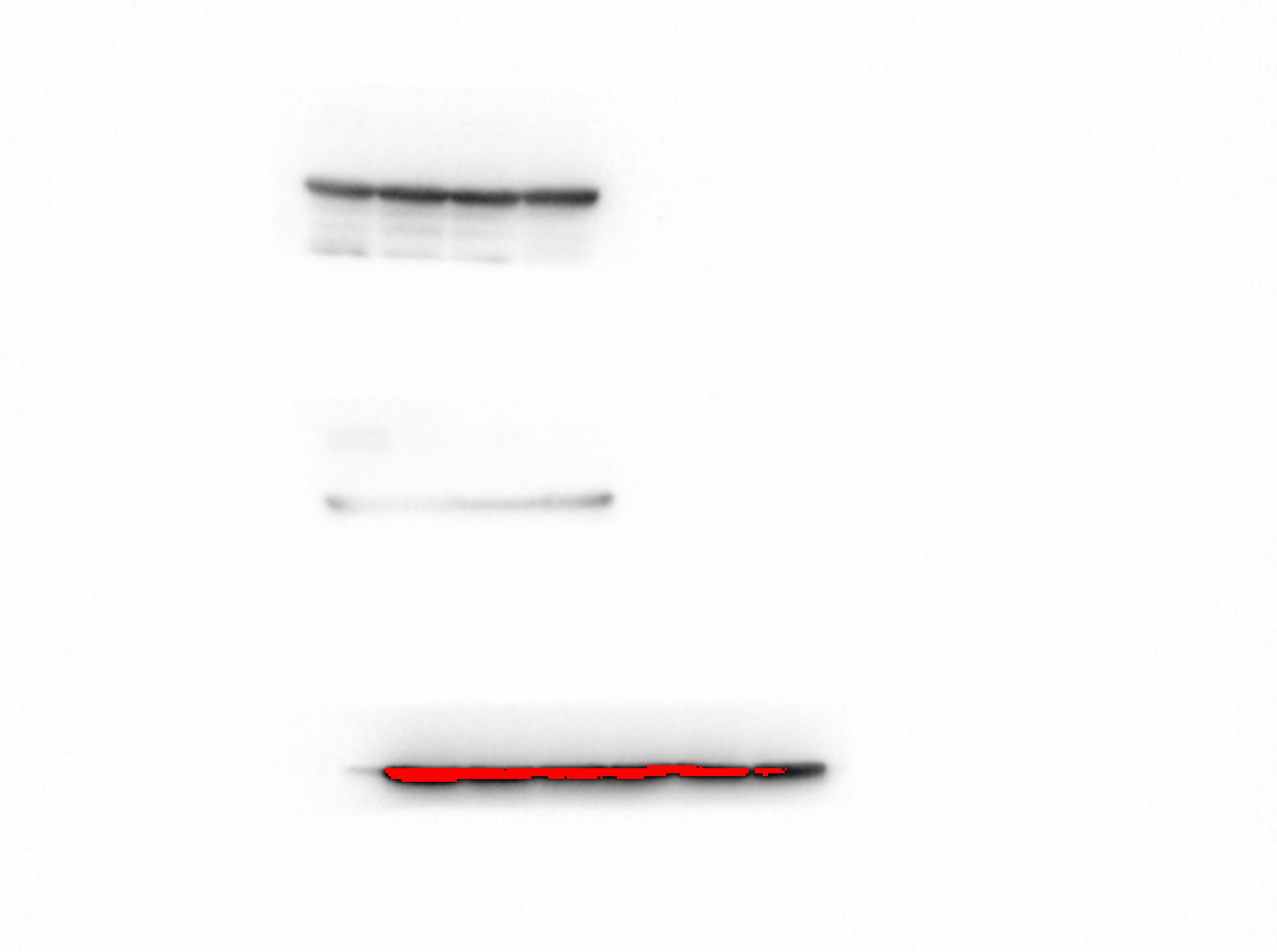

Supplement: Supplementary file 2 [file DataSheet4.ZIP › 1. Kidney Total proteins WB scans/GAPDH-2.tif]

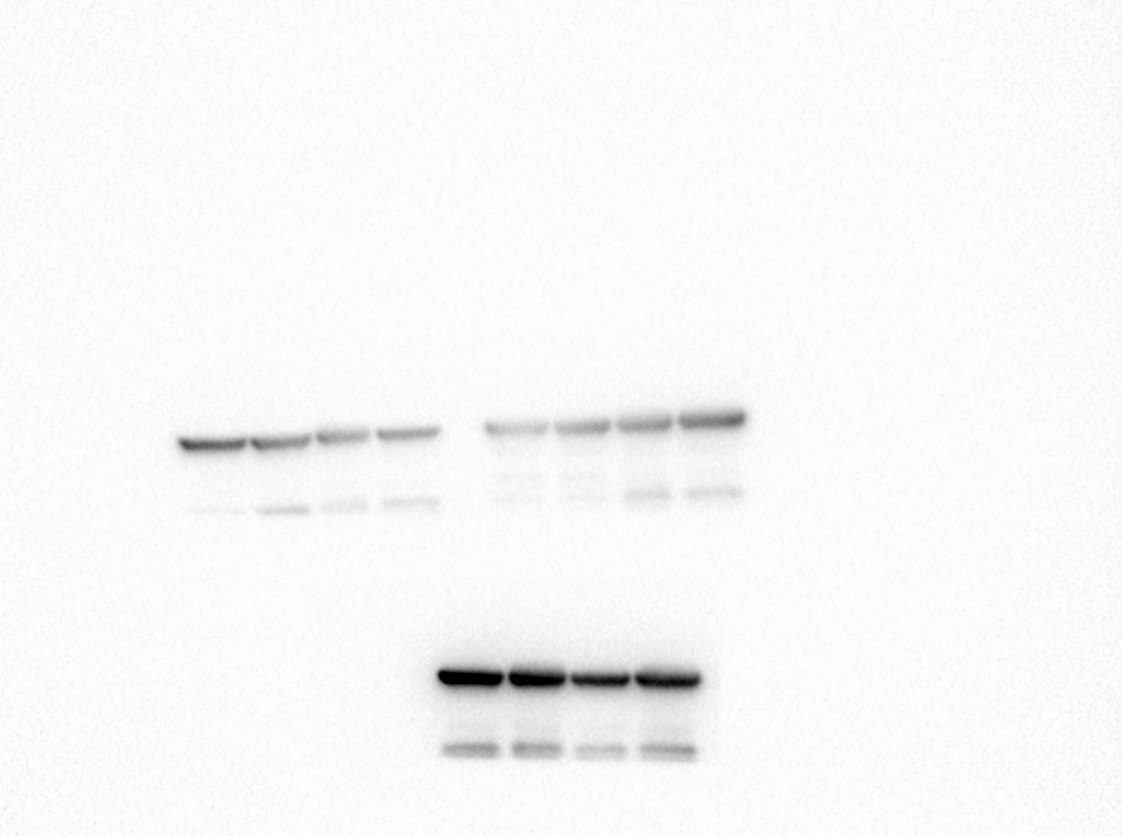

Supplement: Supplementary file 2 [file DataSheet4.ZIP › 1. Kidney Total proteins WB scans/GAPDH-3.tif]

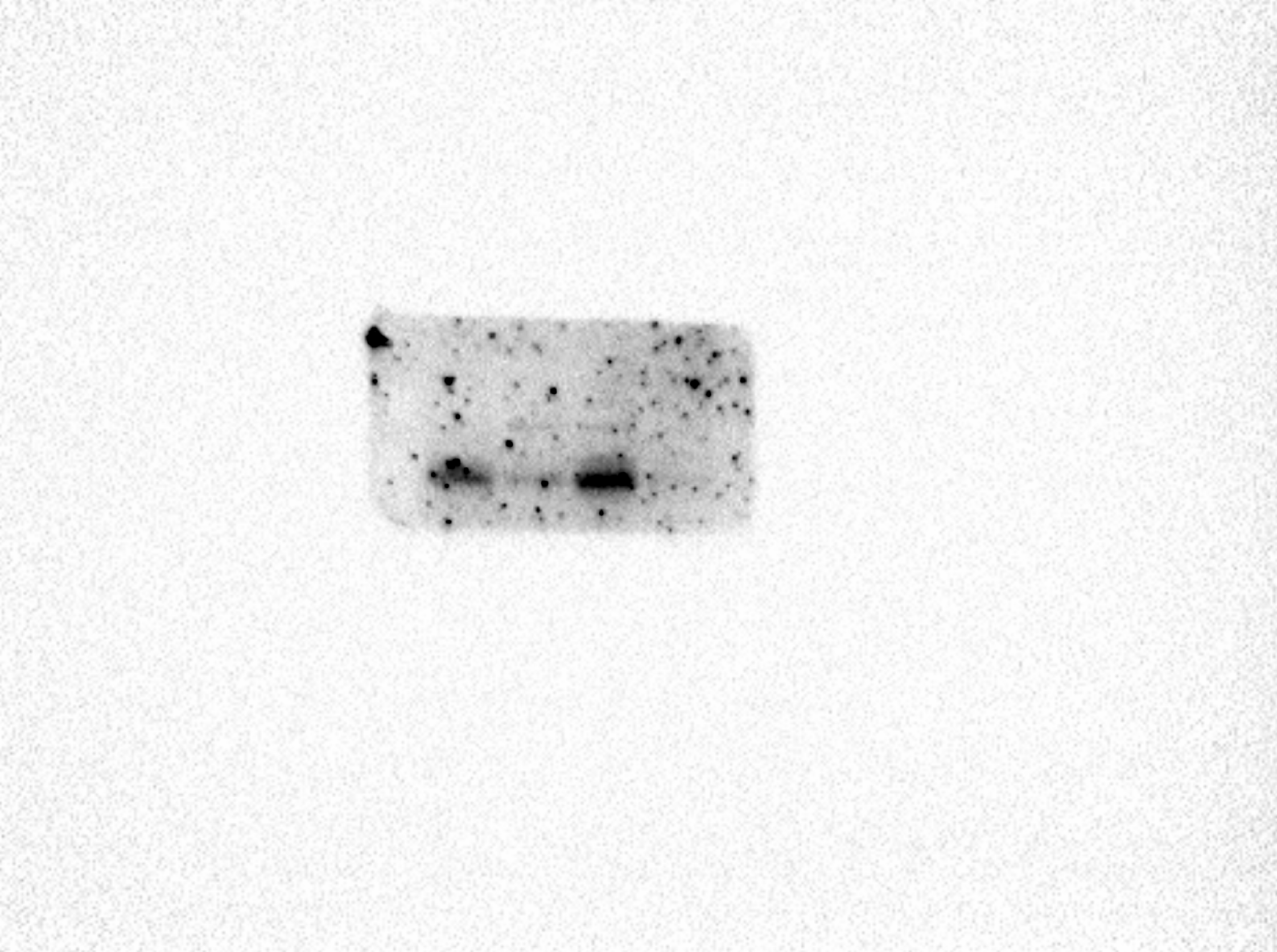

Supplement: Supplementary file 2 [file DataSheet4.ZIP › 1. Kidney Total proteins WB scans/HO-1-1.tif]

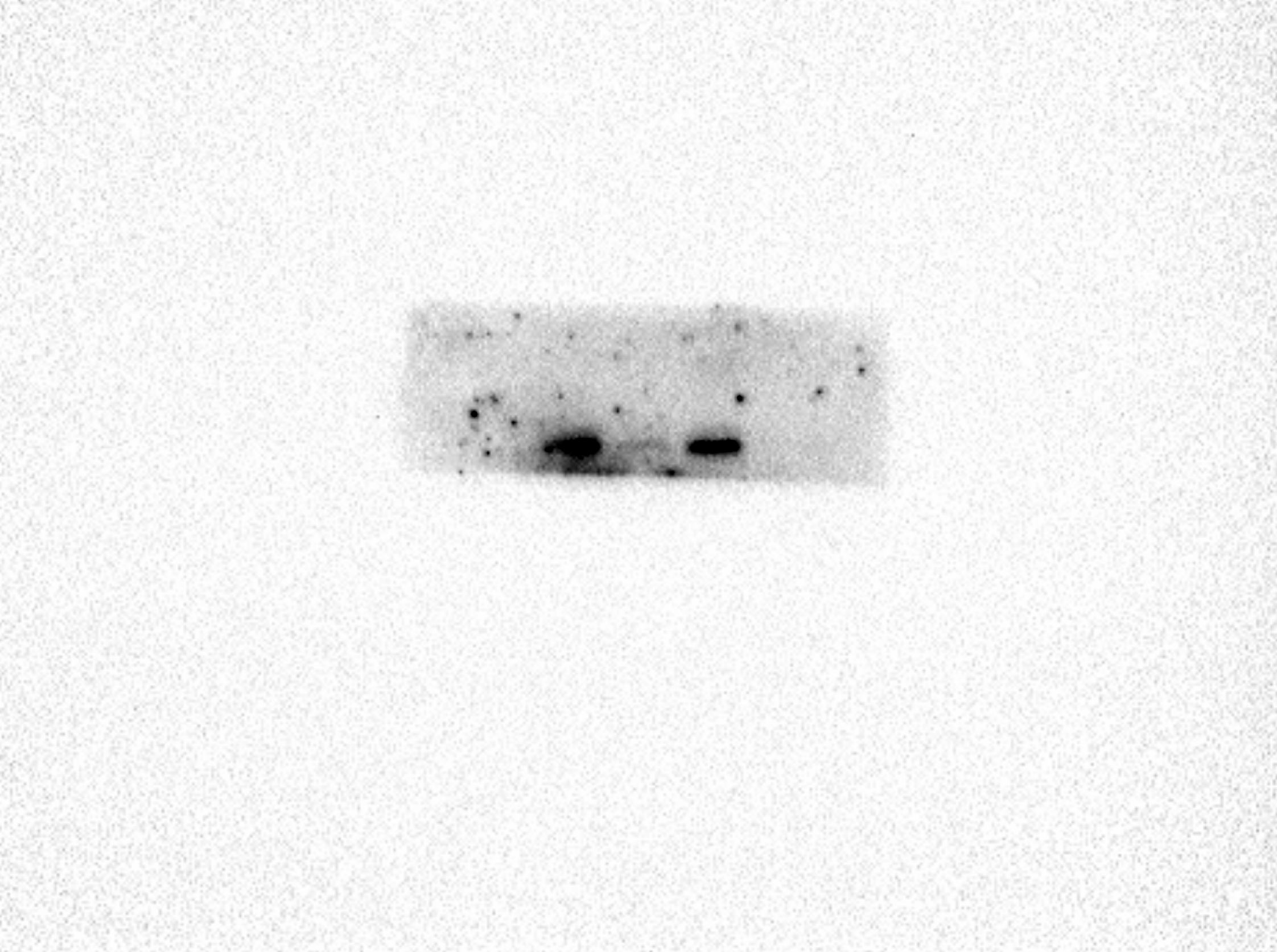

Supplement: Supplementary file 2 [file DataSheet4.ZIP › 1. Kidney Total proteins WB scans/HO-1-2.tif]

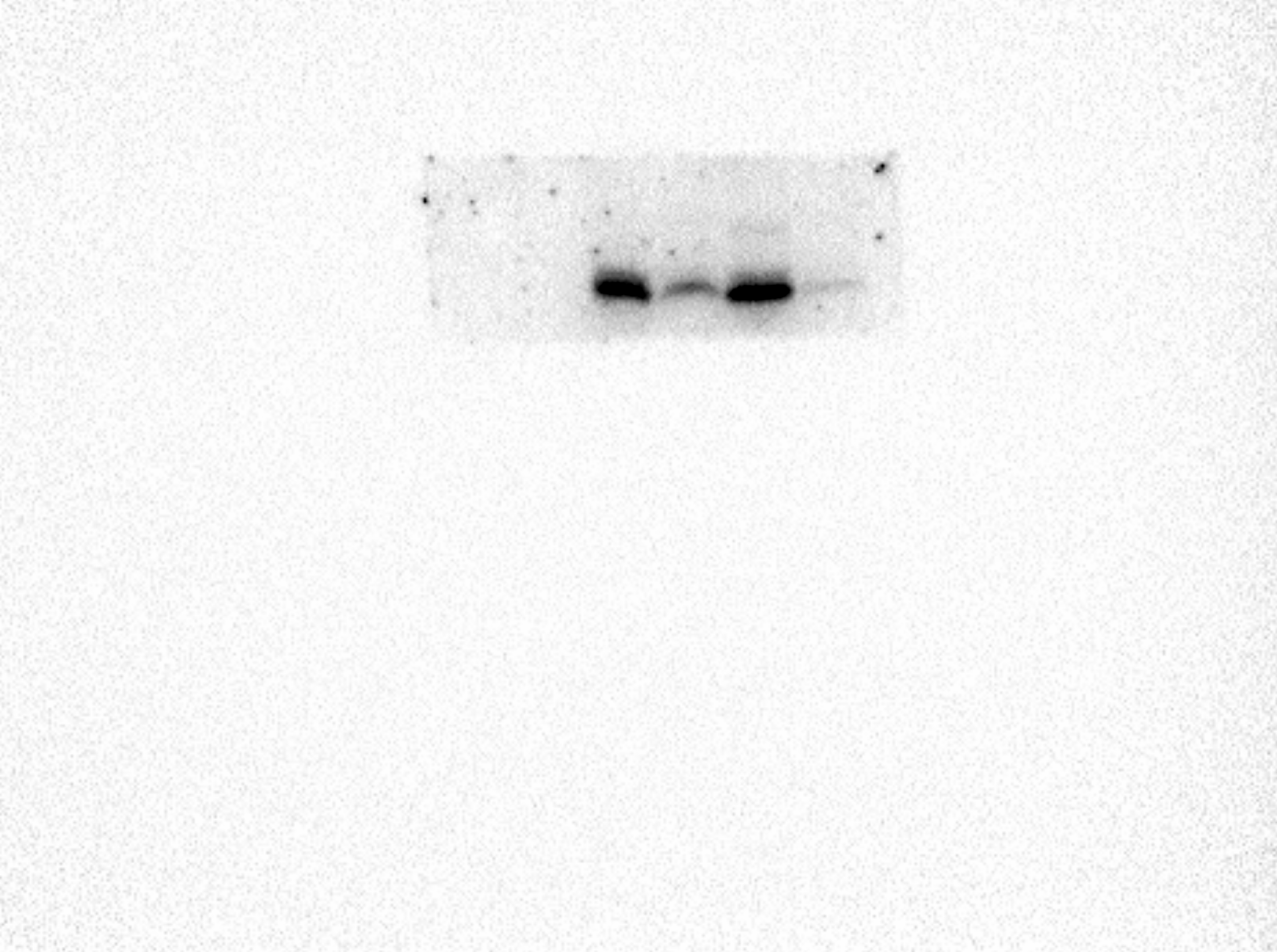

Supplement: Supplementary file 2 [file DataSheet4.ZIP › 1. Kidney Total proteins WB scans/HO-1-3.tif]

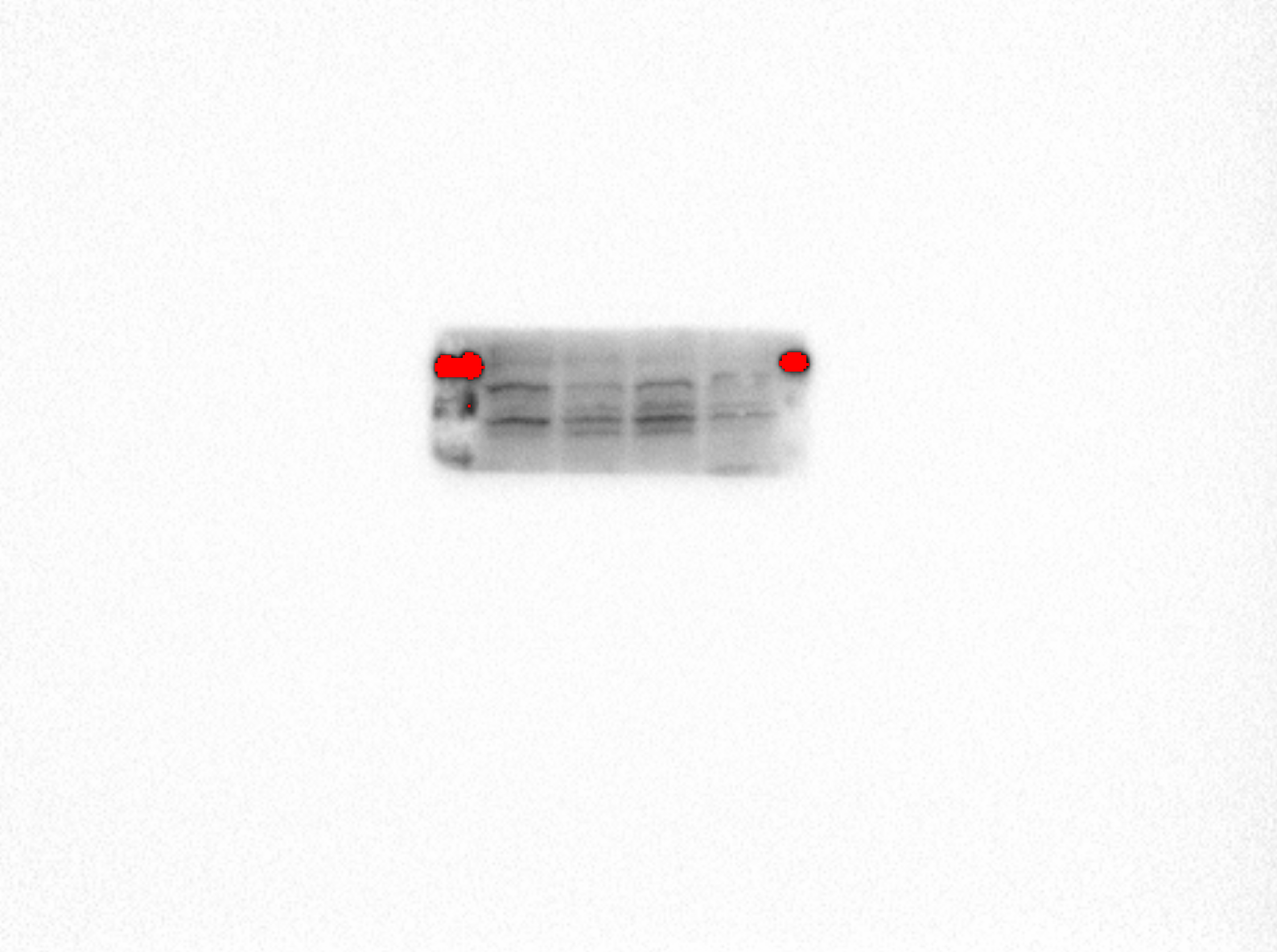

Supplement: Supplementary file 2 [file DataSheet4.ZIP › 1. Kidney Total proteins WB scans/Total Nrf2-1.tif]

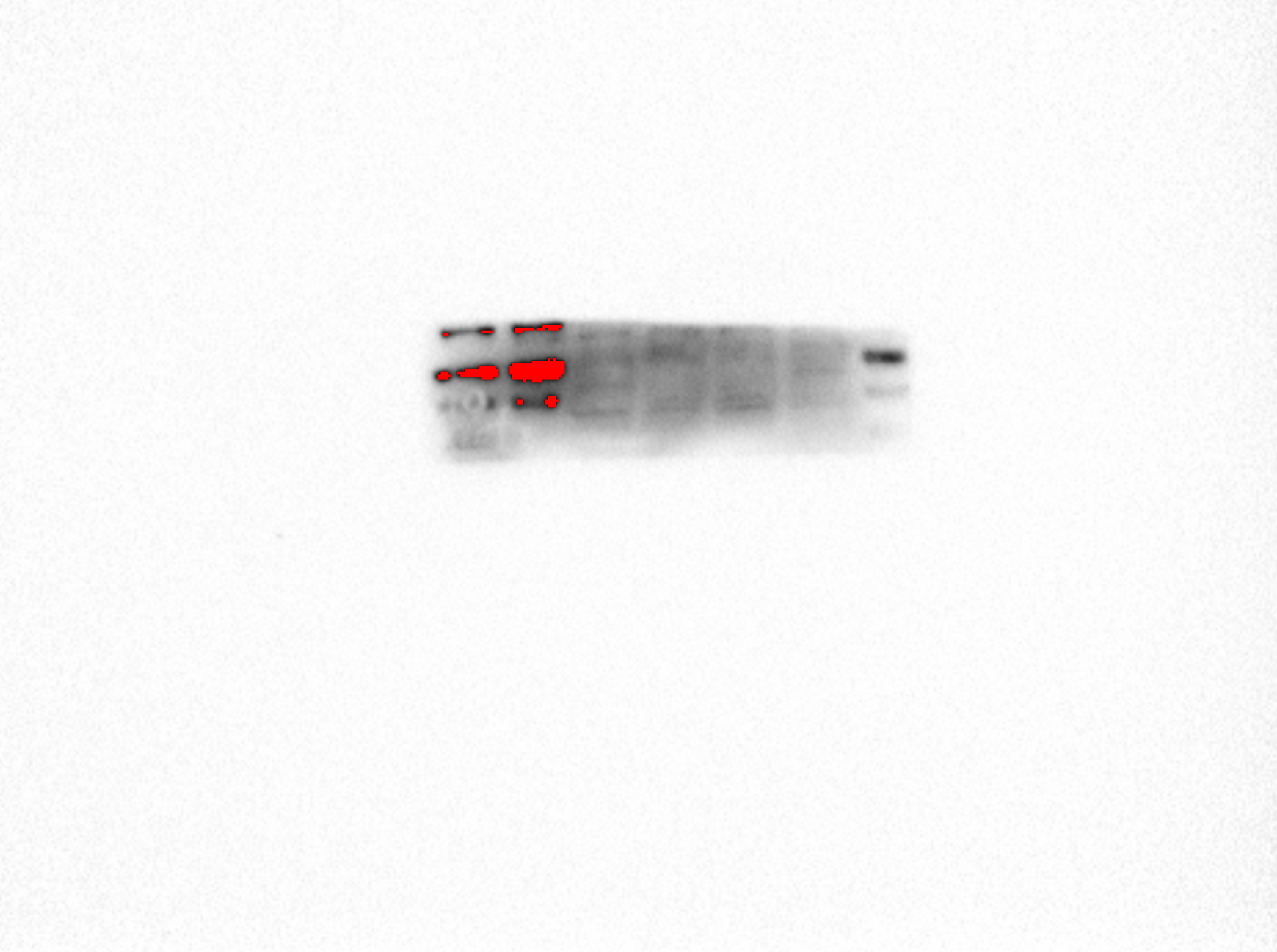

Supplement: Supplementary file 2 [file DataSheet4.ZIP › 1. Kidney Total proteins WB scans/Total Nrf2-2.tif]

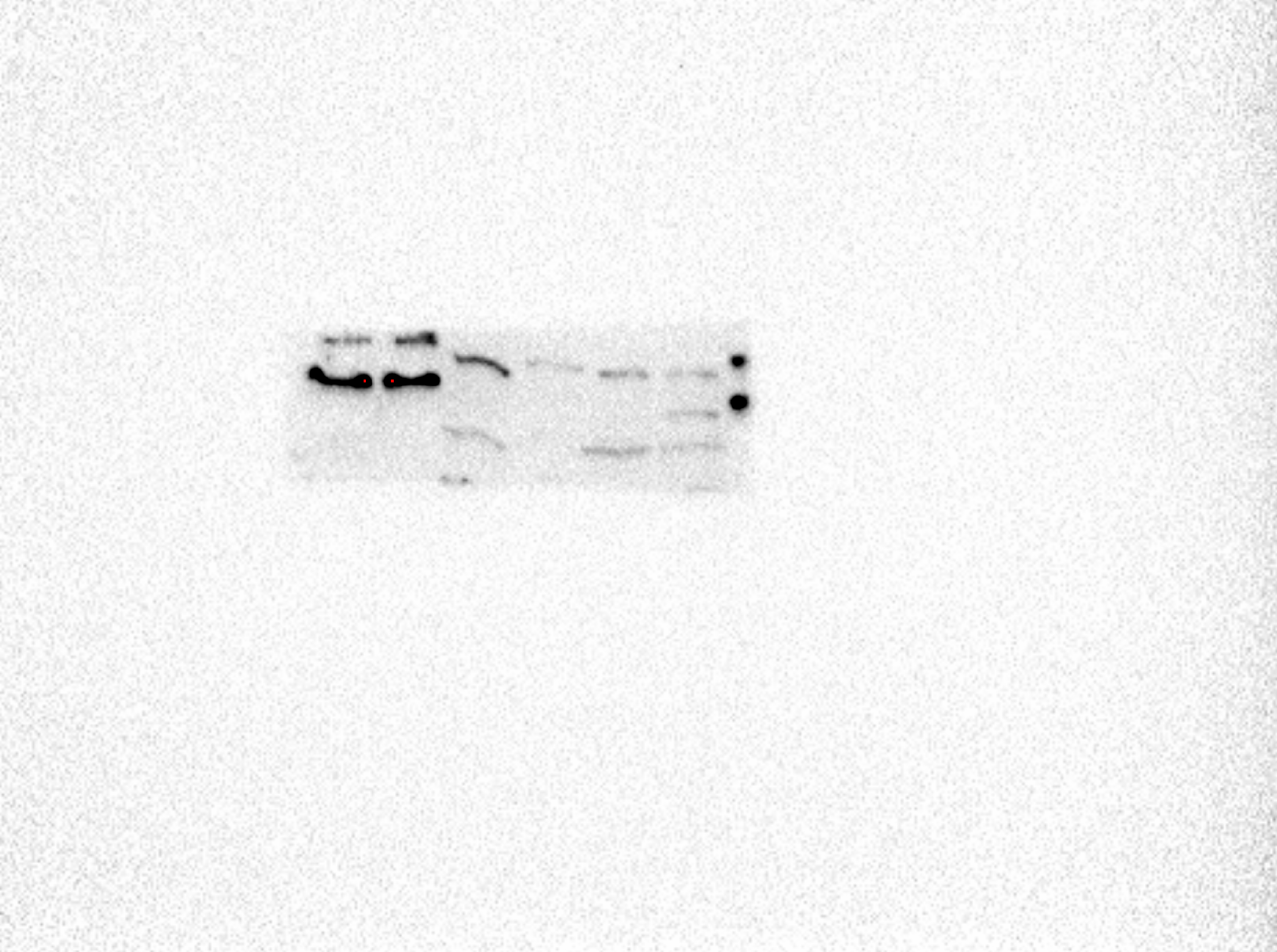

Supplement: Supplementary file 2 [file DataSheet4.ZIP › 1. Kidney Total proteins WB scans/Total Nrf2-3.tif]

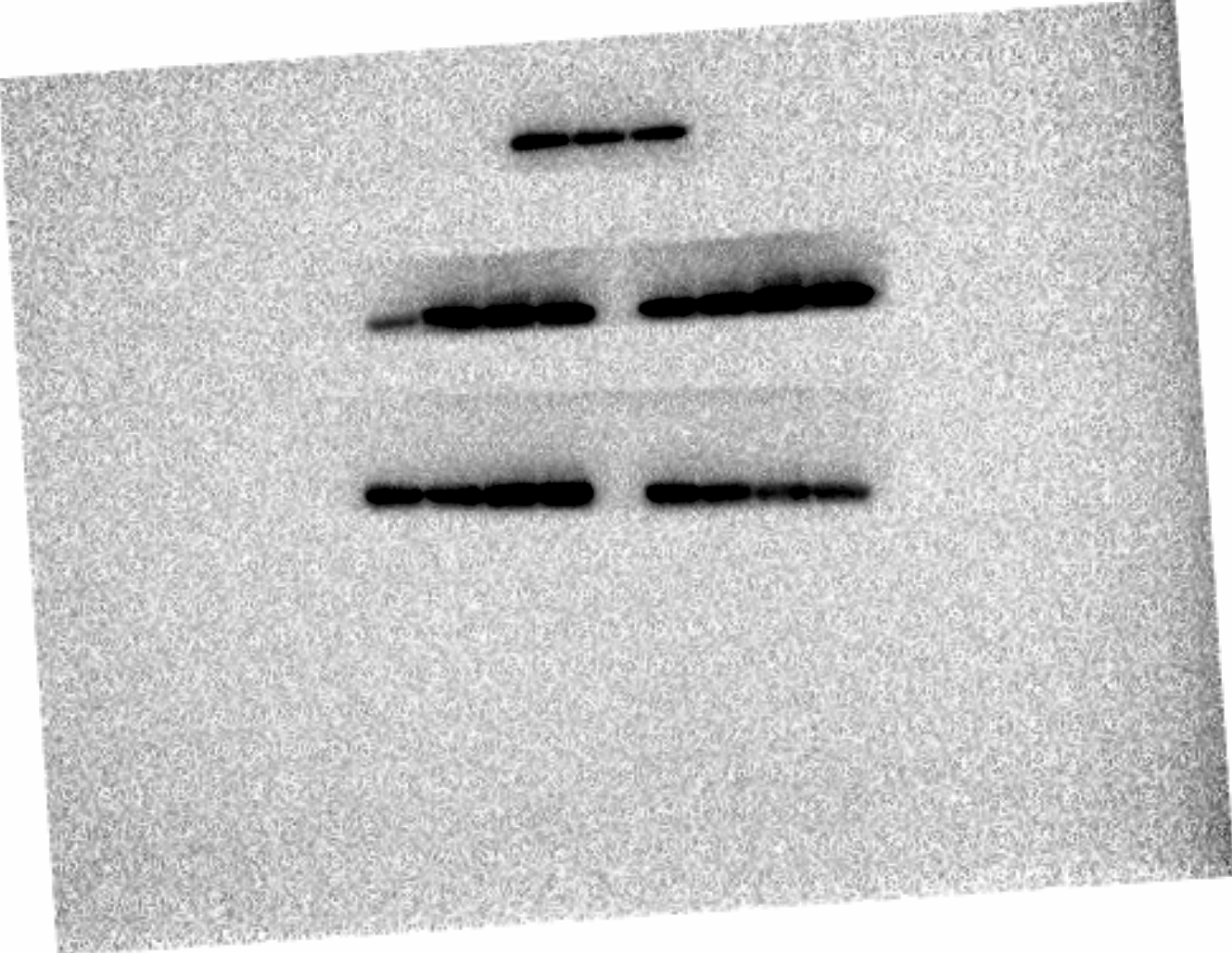

Supplement: Supplementary file 3 [file DataSheet6.ZIP › 3. Podocyte Total Proteins WB scans/Cellular total protein-Caspase3-3.tif]

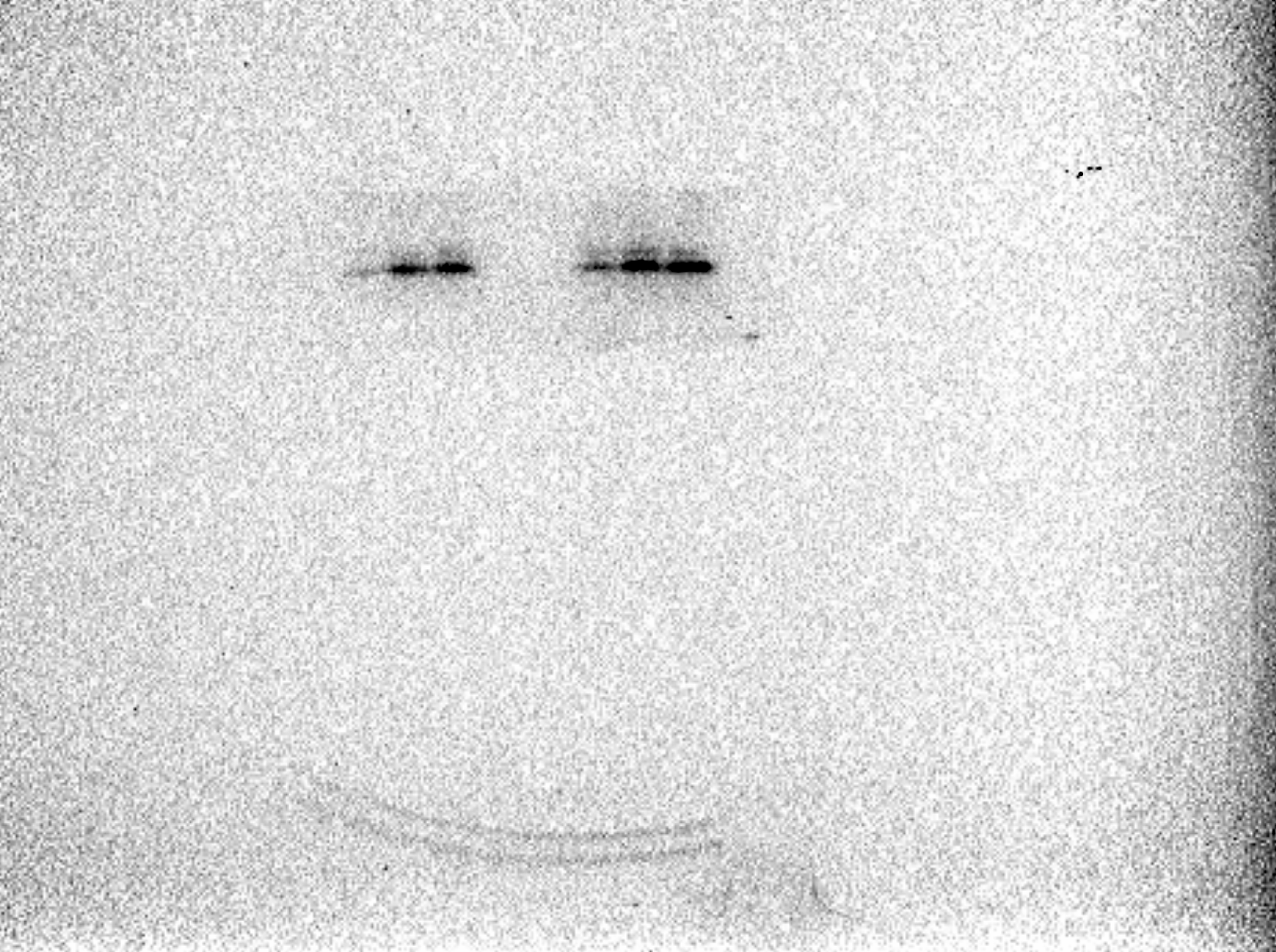

Supplement: Supplementary file 3 [file DataSheet6.ZIP › 3. Podocyte Total Proteins WB scans/Cellular total protein-cleave caspase3-1.tif]

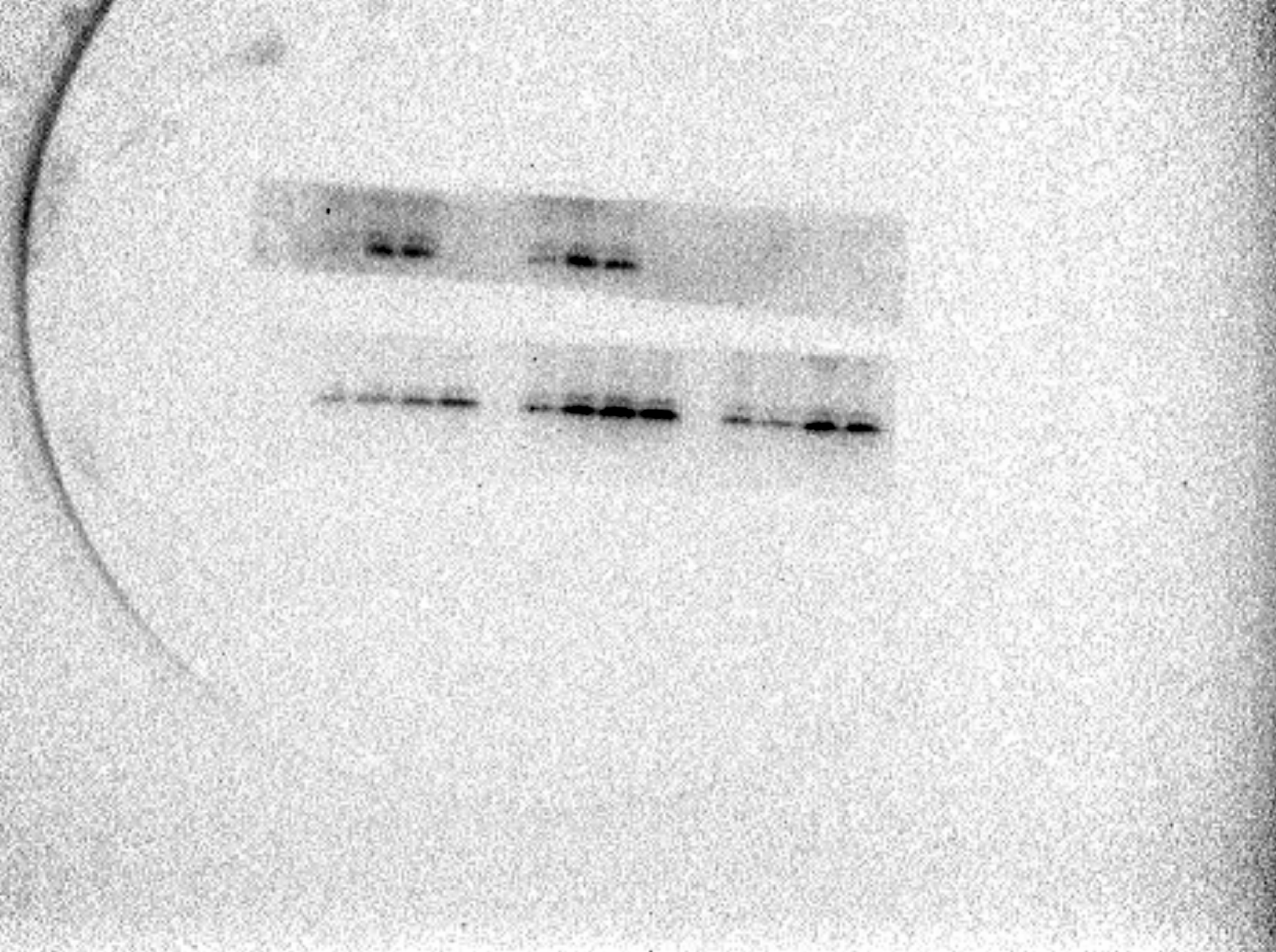

Supplement: Supplementary file 3 [file DataSheet6.ZIP › 3. Podocyte Total Proteins WB scans/Cellular total protein-cleave caspase3-2.tif]

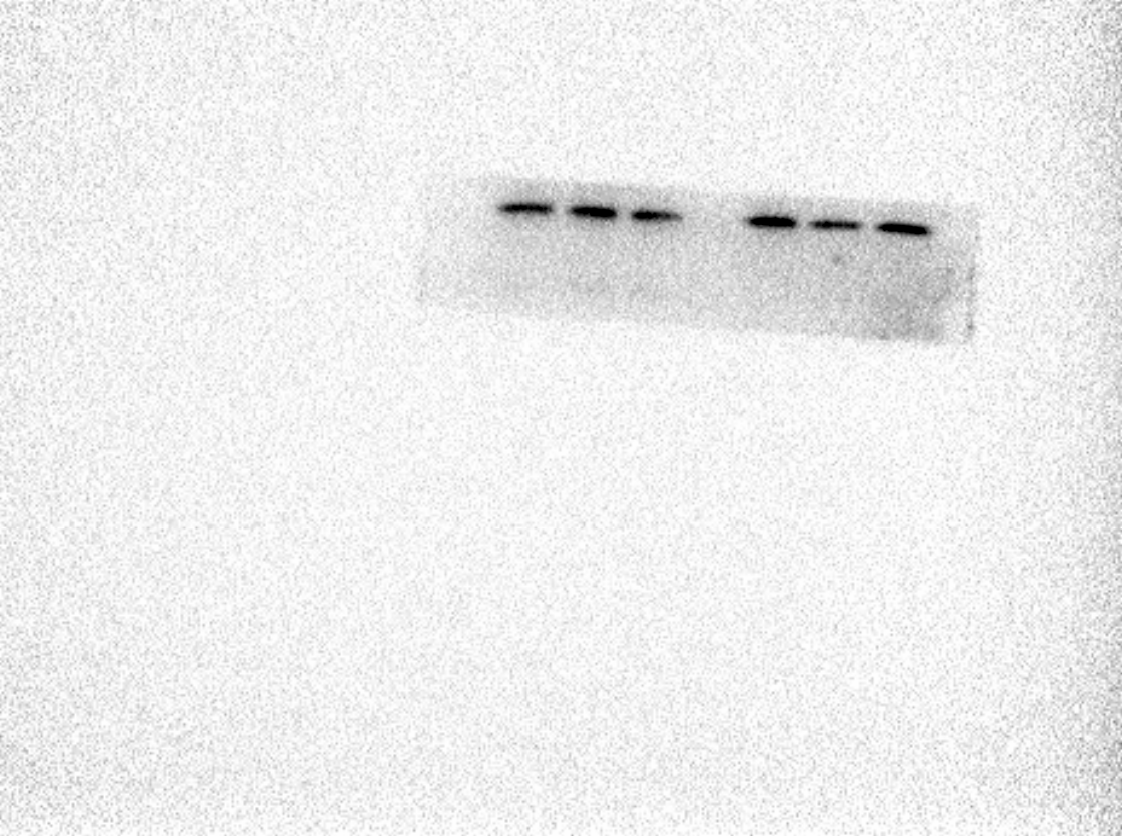

Supplement: Supplementary file 3 [file DataSheet6.ZIP › 3. Podocyte Total Proteins WB scans/Cellular total protein-cleave caspase3-3.tif]

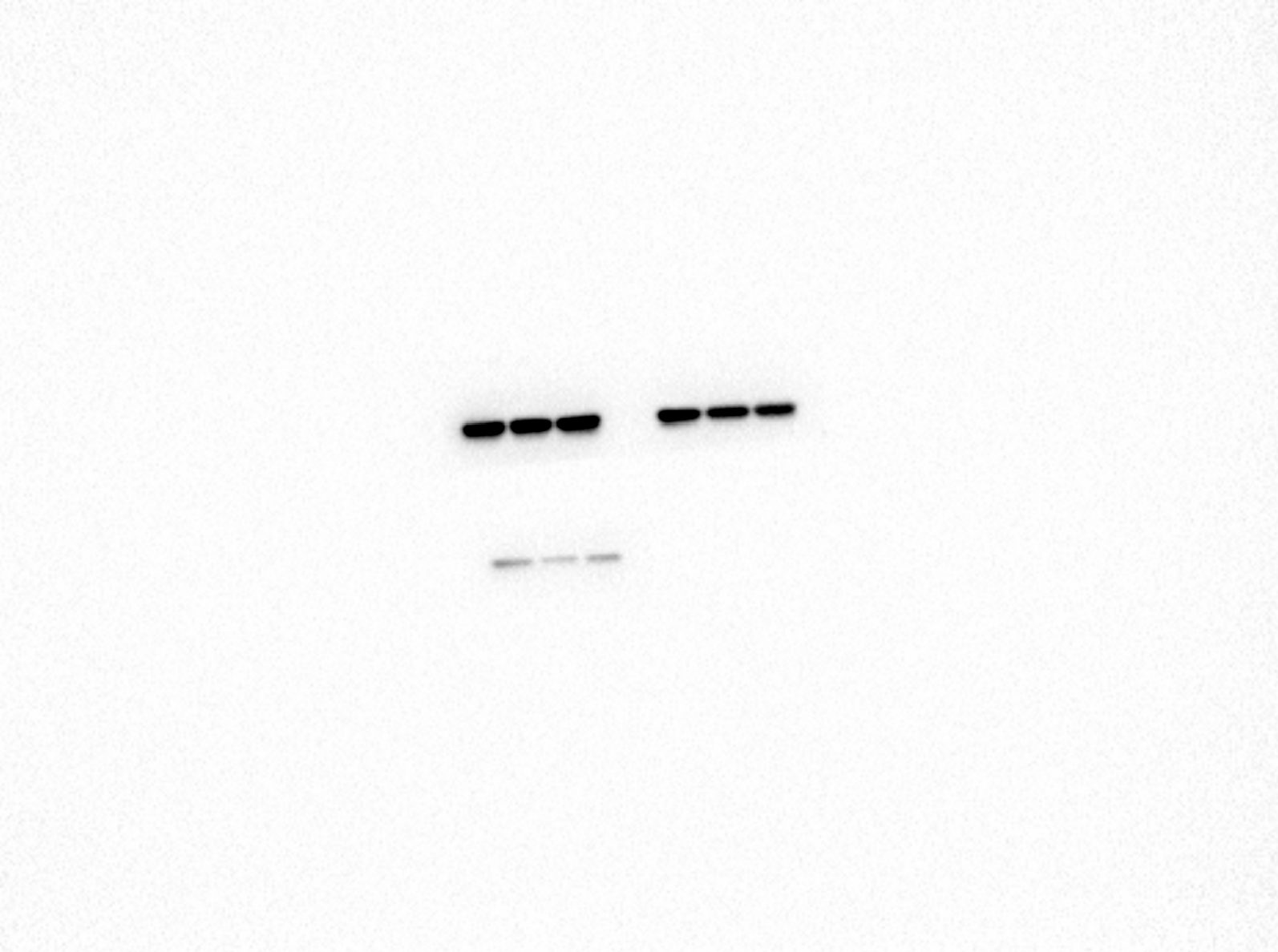

Supplement: Supplementary file 3 [file DataSheet6.ZIP › 3. Podocyte Total Proteins WB scans/Cellular total protein-gapdh-1.tif]

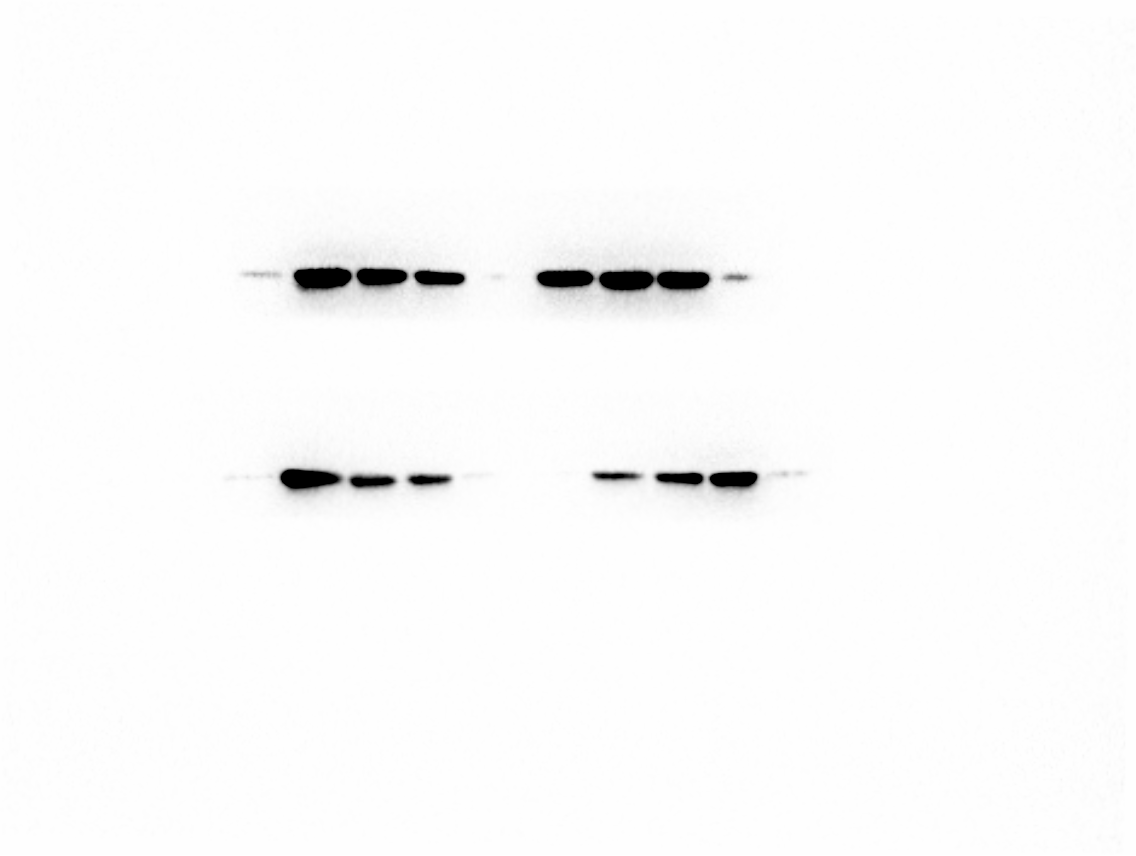

Supplement: Supplementary file 3 [file DataSheet6.ZIP › 3. Podocyte Total Proteins WB scans/Cellular total protein-gapdh-2.tif]

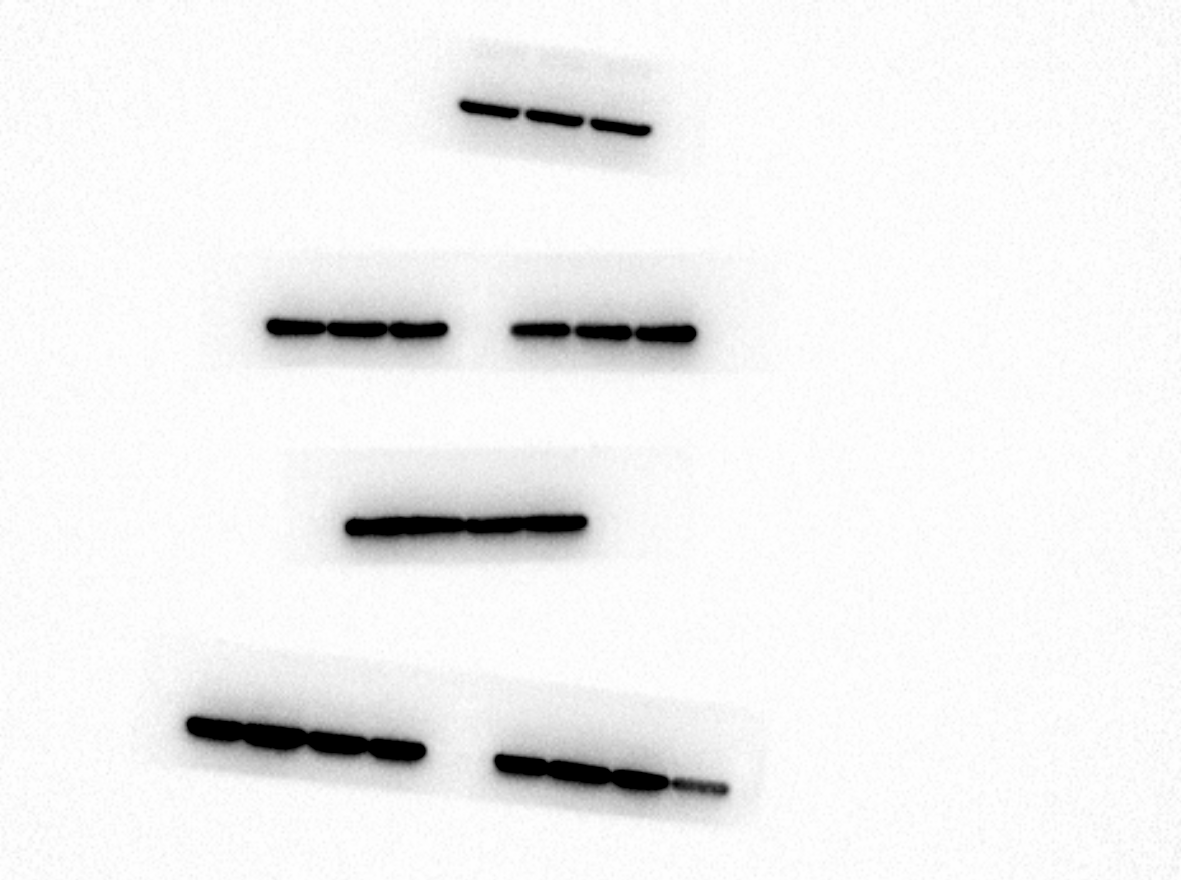

Supplement: Supplementary file 3 [file DataSheet6.ZIP › 3. Podocyte Total Proteins WB scans/Cellular total protein-gapdh-3.tif]

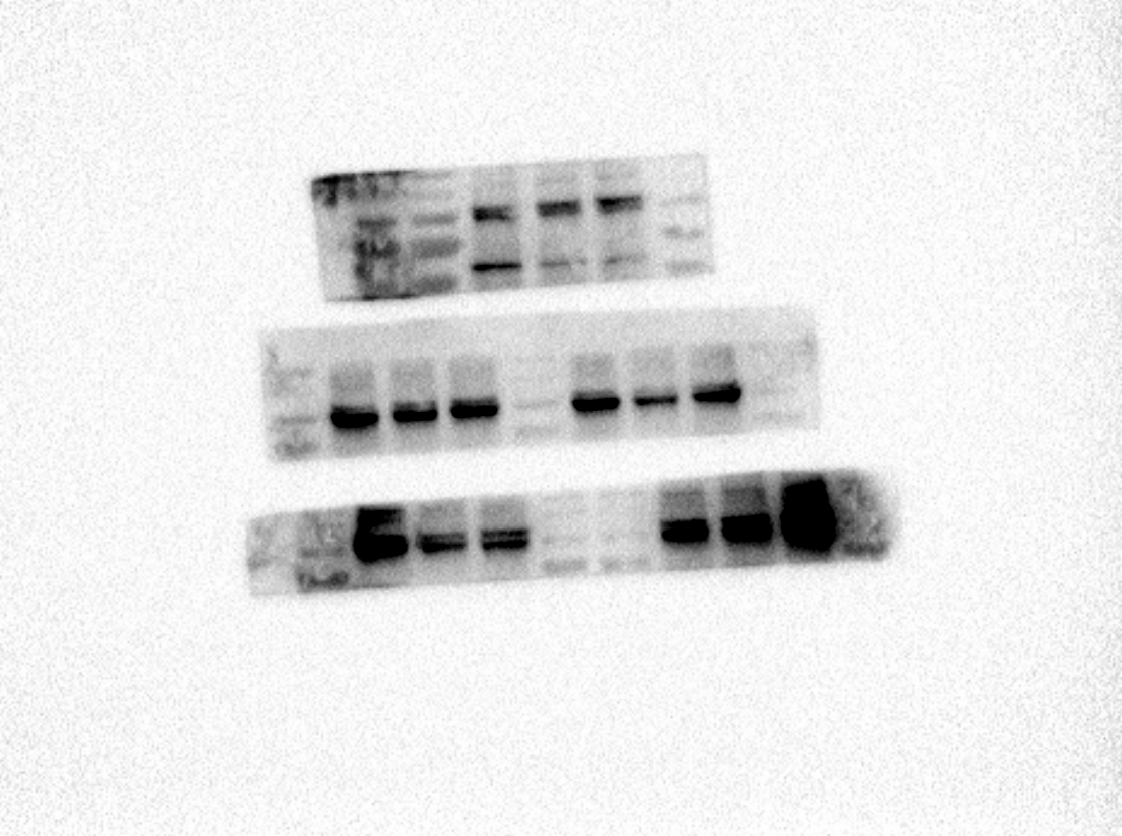

Supplement: Supplementary file 3 [file DataSheet6.ZIP › 3. Podocyte Total Proteins WB scans/Cellular total protein-nrf2-1 2.tif]

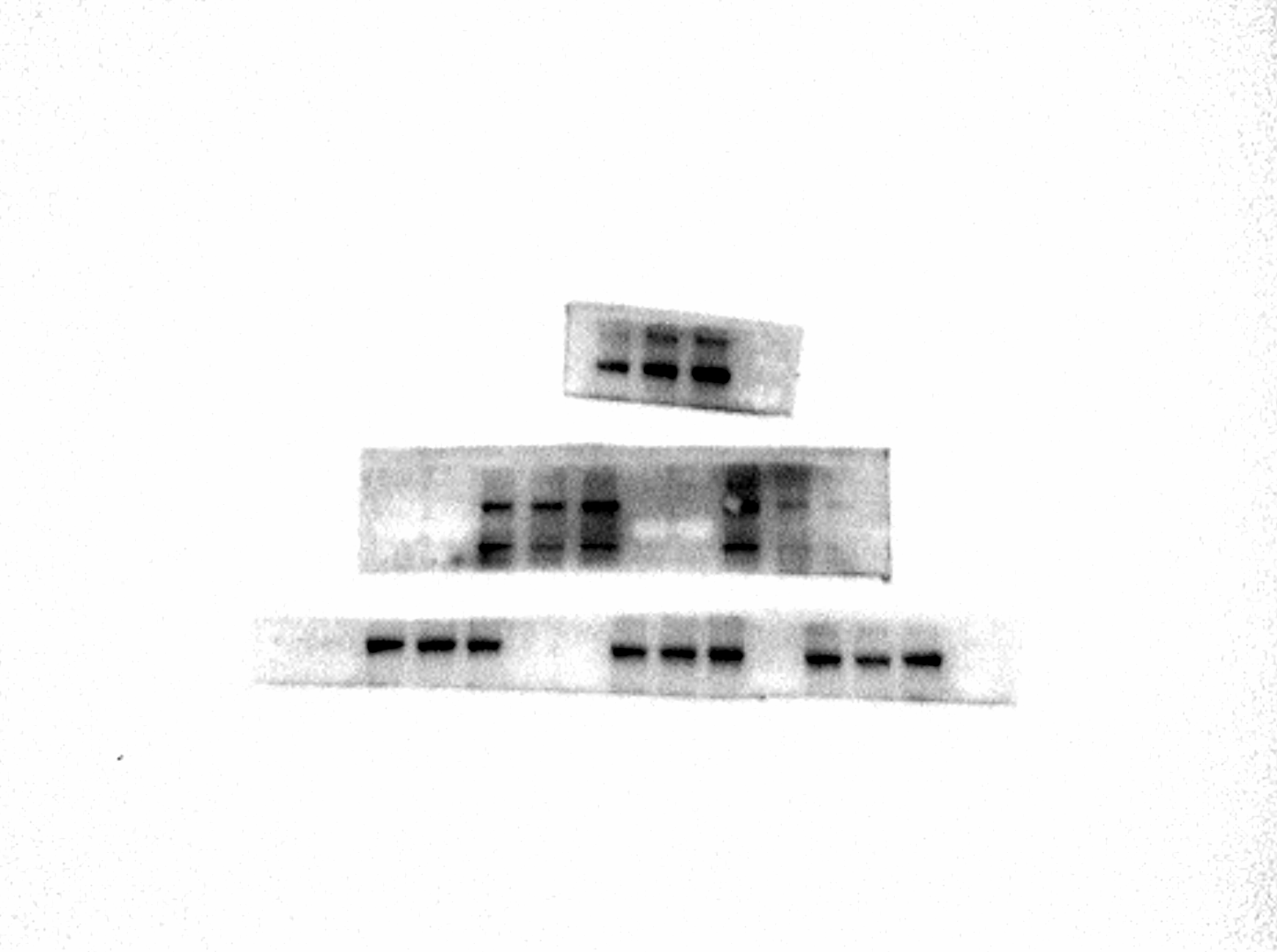

Supplement: Supplementary file 3 [file DataSheet6.ZIP › 3. Podocyte Total Proteins WB scans/Cellular total protein-nrf2-3.tif]

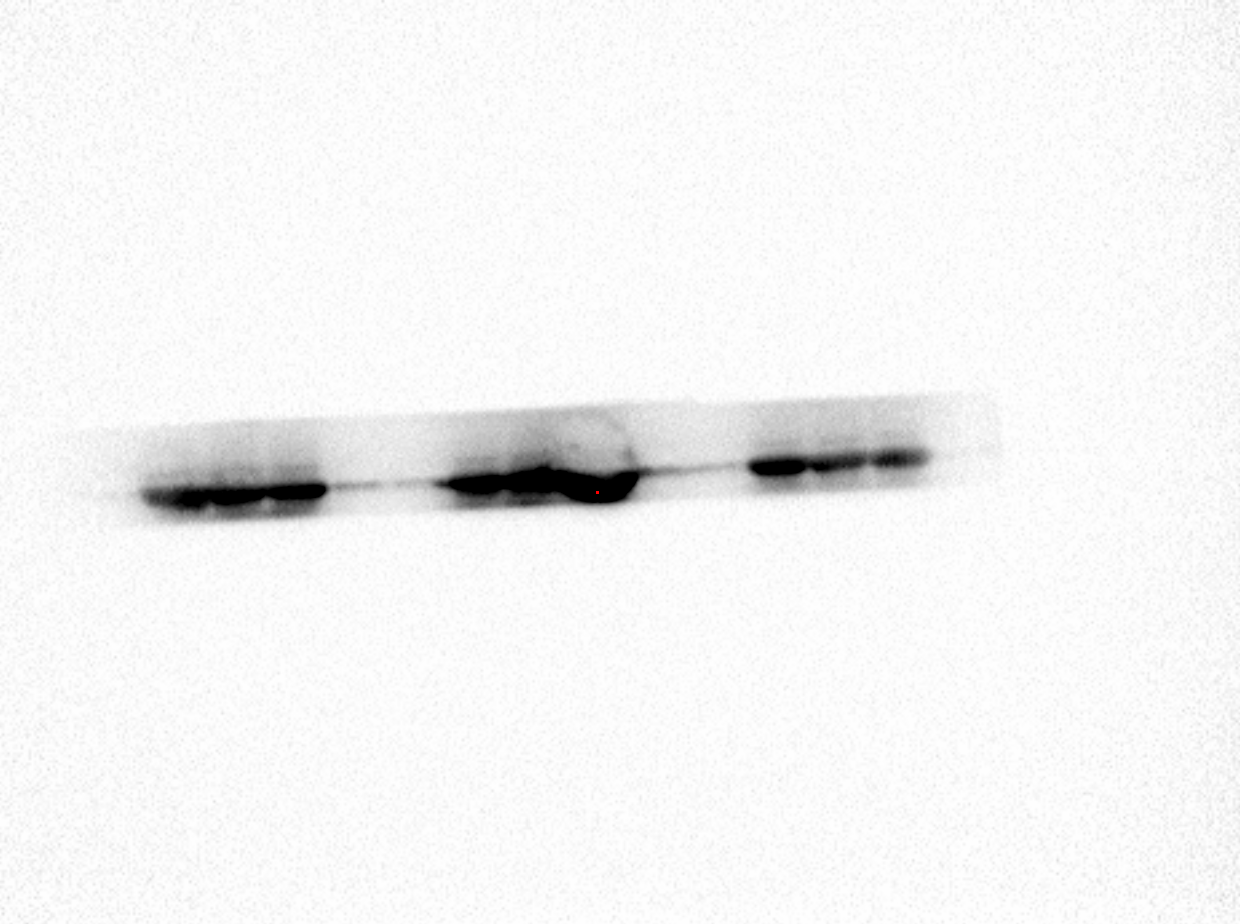

Supplement: Supplementary file 3 [file DataSheet6.ZIP › 3. Podocyte Total Proteins WB scans/Cellular total protein-pro caspase3-1.tif]

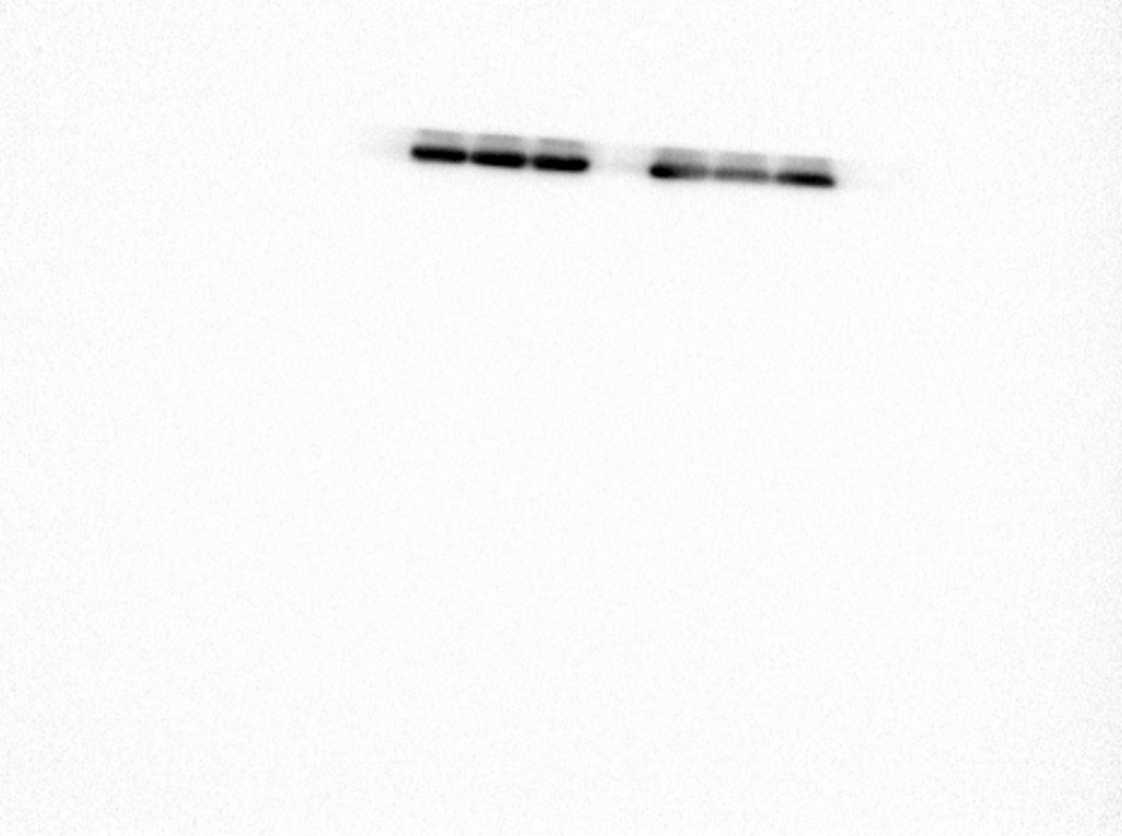

Supplement: Supplementary file 3 [file DataSheet6.ZIP › 3. Podocyte Total Proteins WB scans/Cellular total protein-pro caspase3-2.tif]

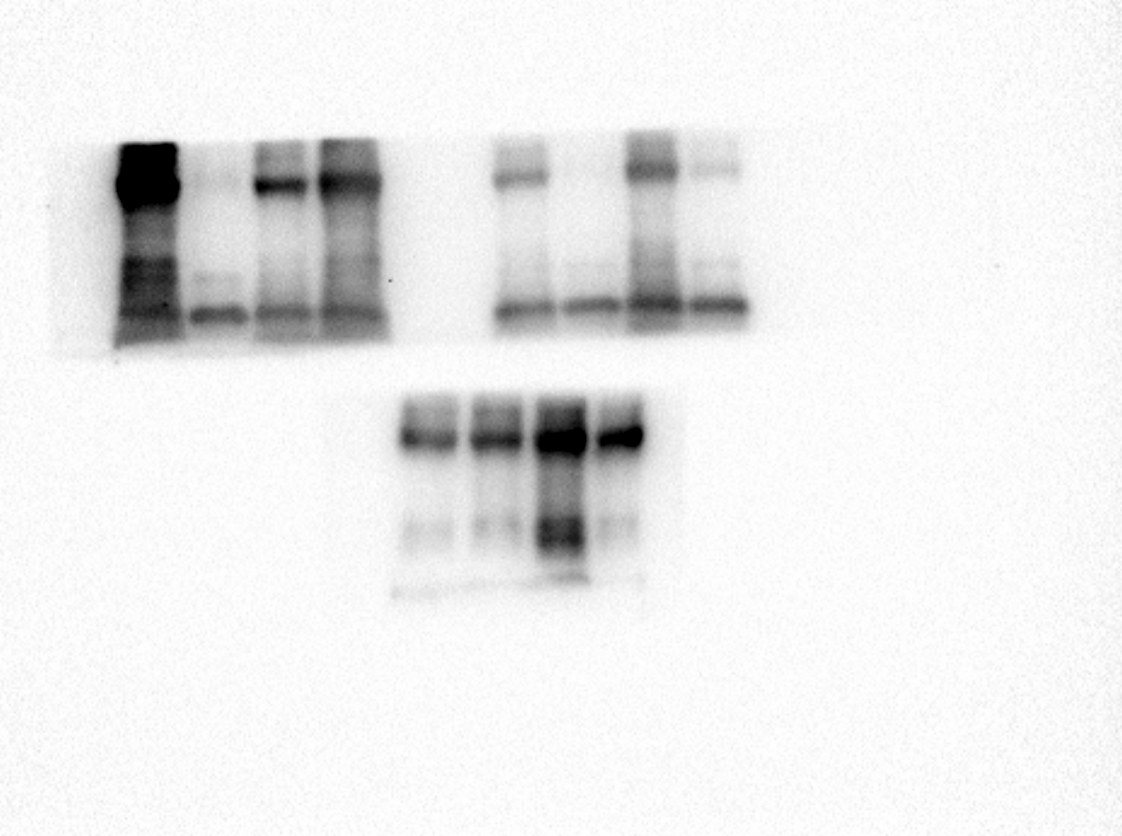

Supplement: Supplementary file 7 [file DataSheet5.ZIP › 2. Kidney Nucleus Proteins WB scans/Tissue nuclear Histone H3-1 2.tif]

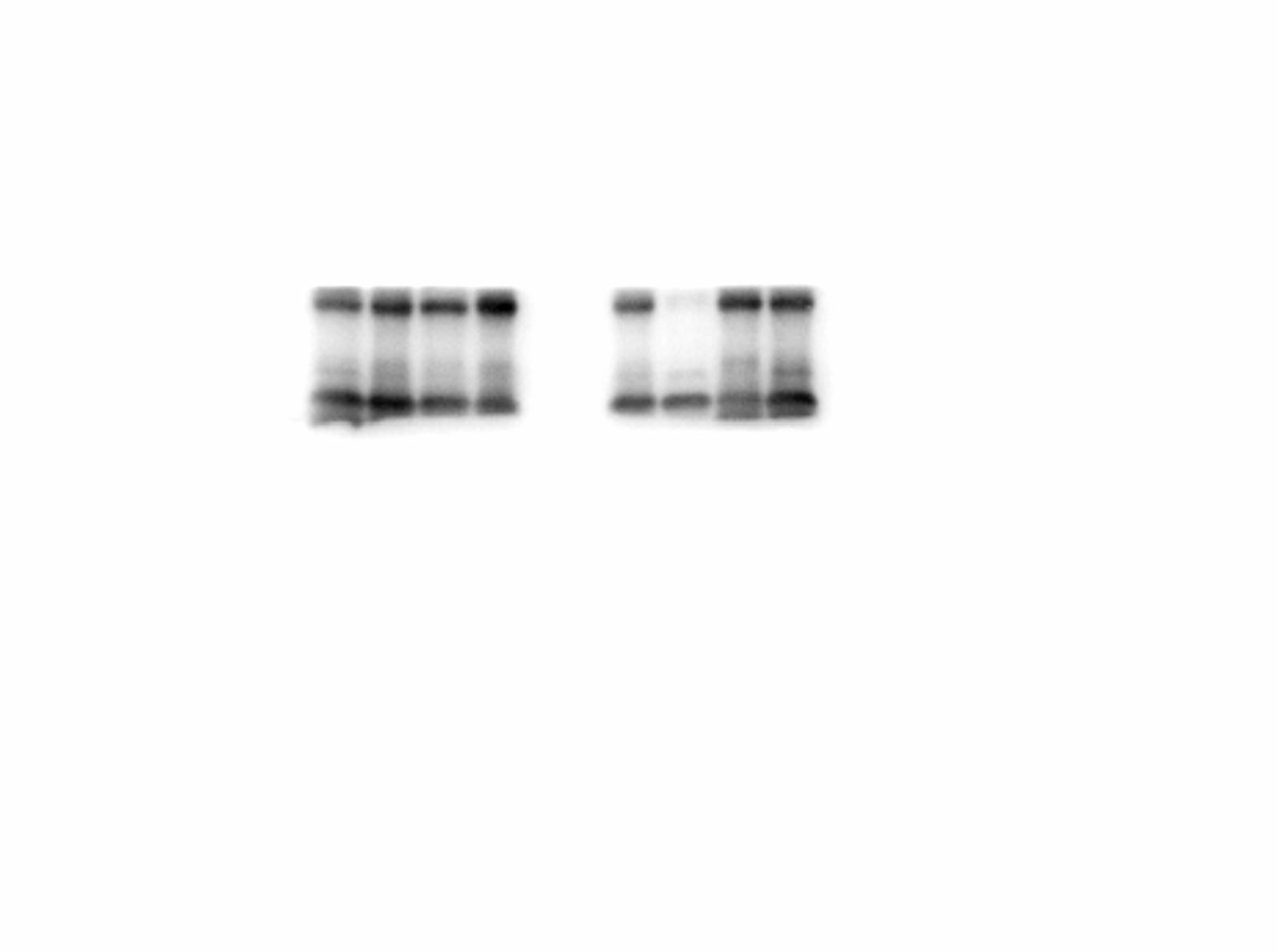

Supplement: Supplementary file 7 [file DataSheet5.ZIP › 2. Kidney Nucleus Proteins WB scans/Tissue nuclear Histone H3-3.tif]

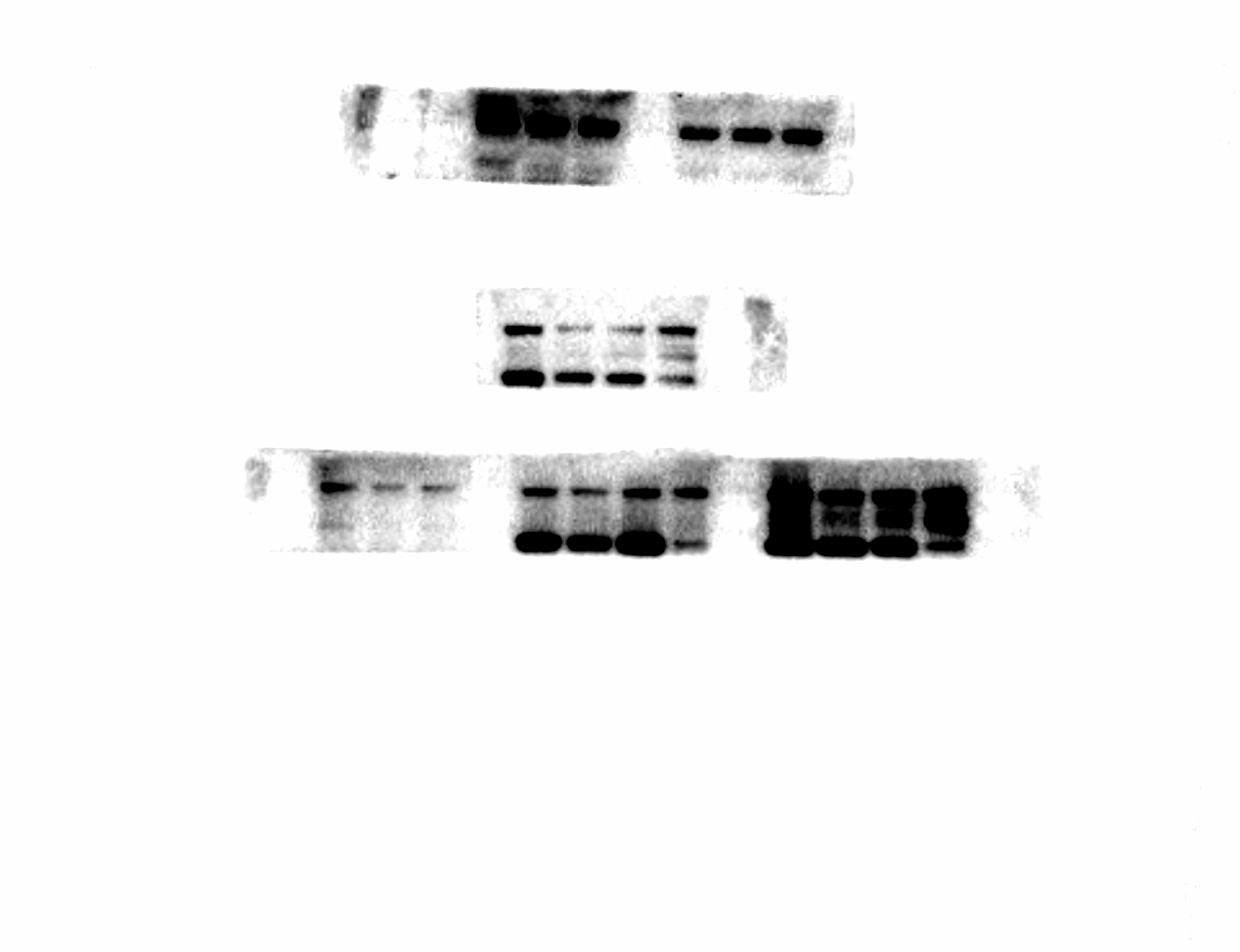

Supplement: Supplementary file 7 [file DataSheet5.ZIP › 2. Kidney Nucleus Proteins WB scans/Tissue nuclear NRF2-1.tif]

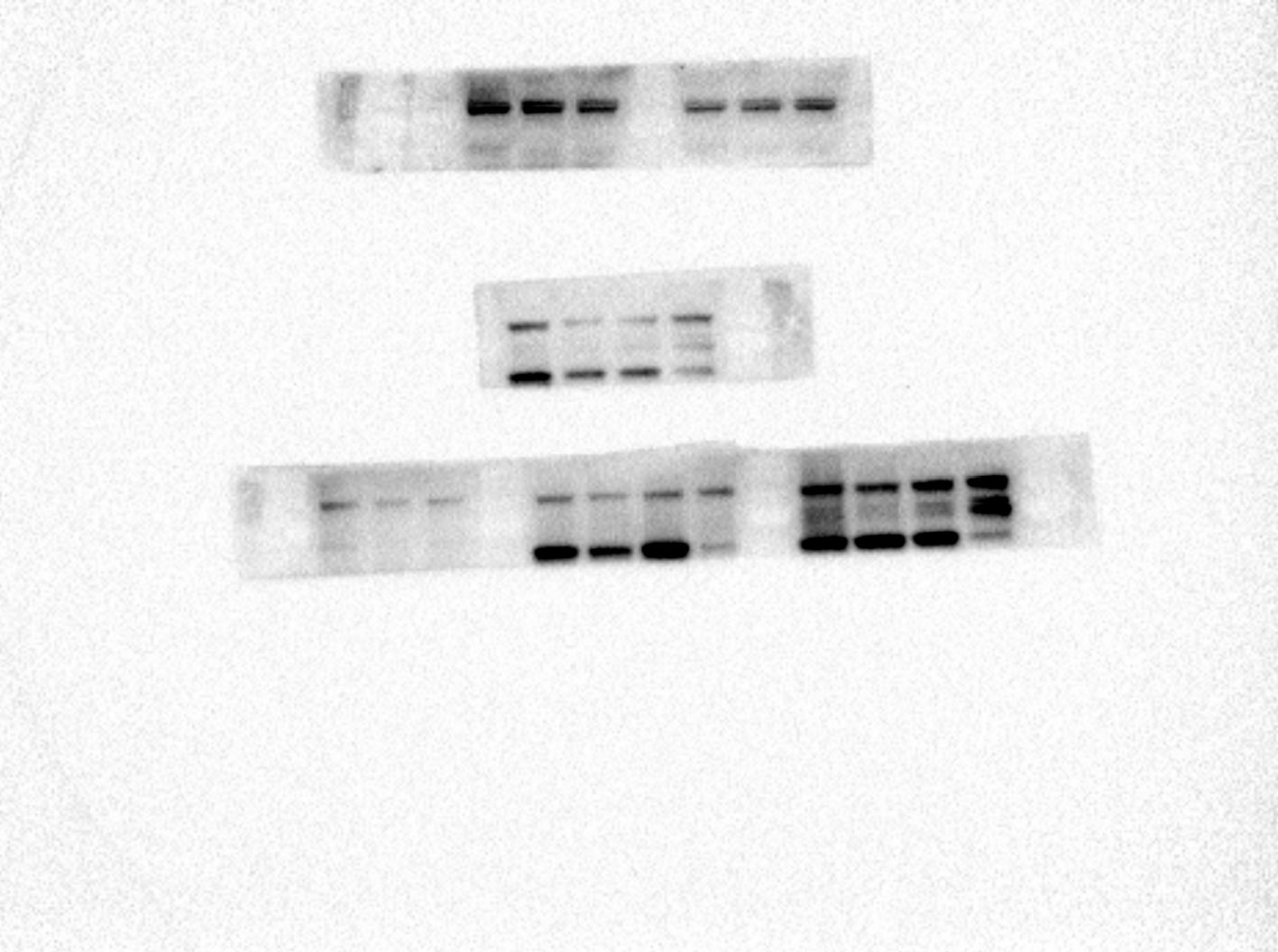

Supplement: Supplementary file 7 [file DataSheet5.ZIP › 2. Kidney Nucleus Proteins WB scans/Tissue nuclear NRF2-2.tif]

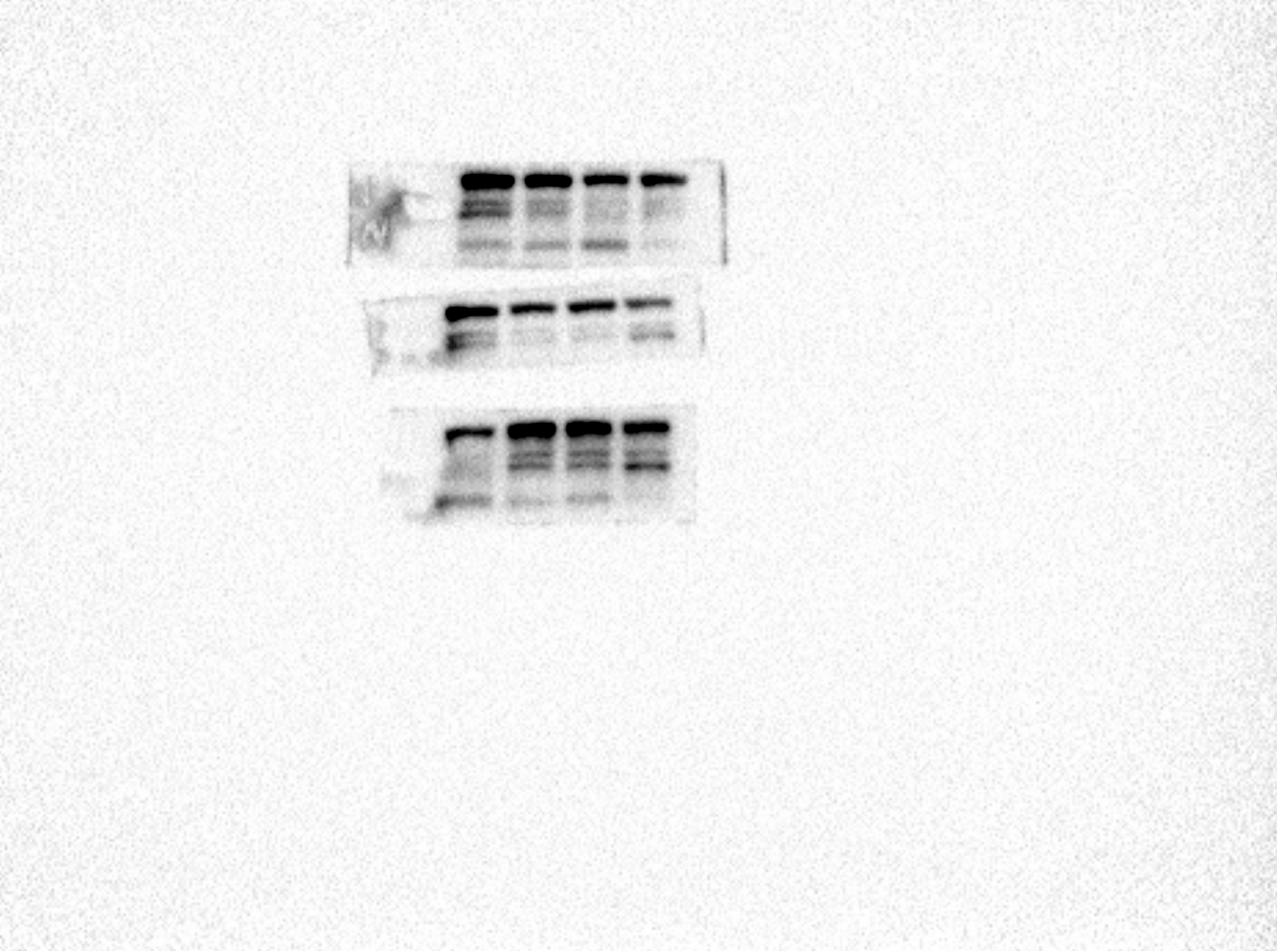

Supplement: Supplementary file 7 [file DataSheet5.ZIP › 2. Kidney Nucleus Proteins WB scans/Tissue nuclear NRF2-3.tif]

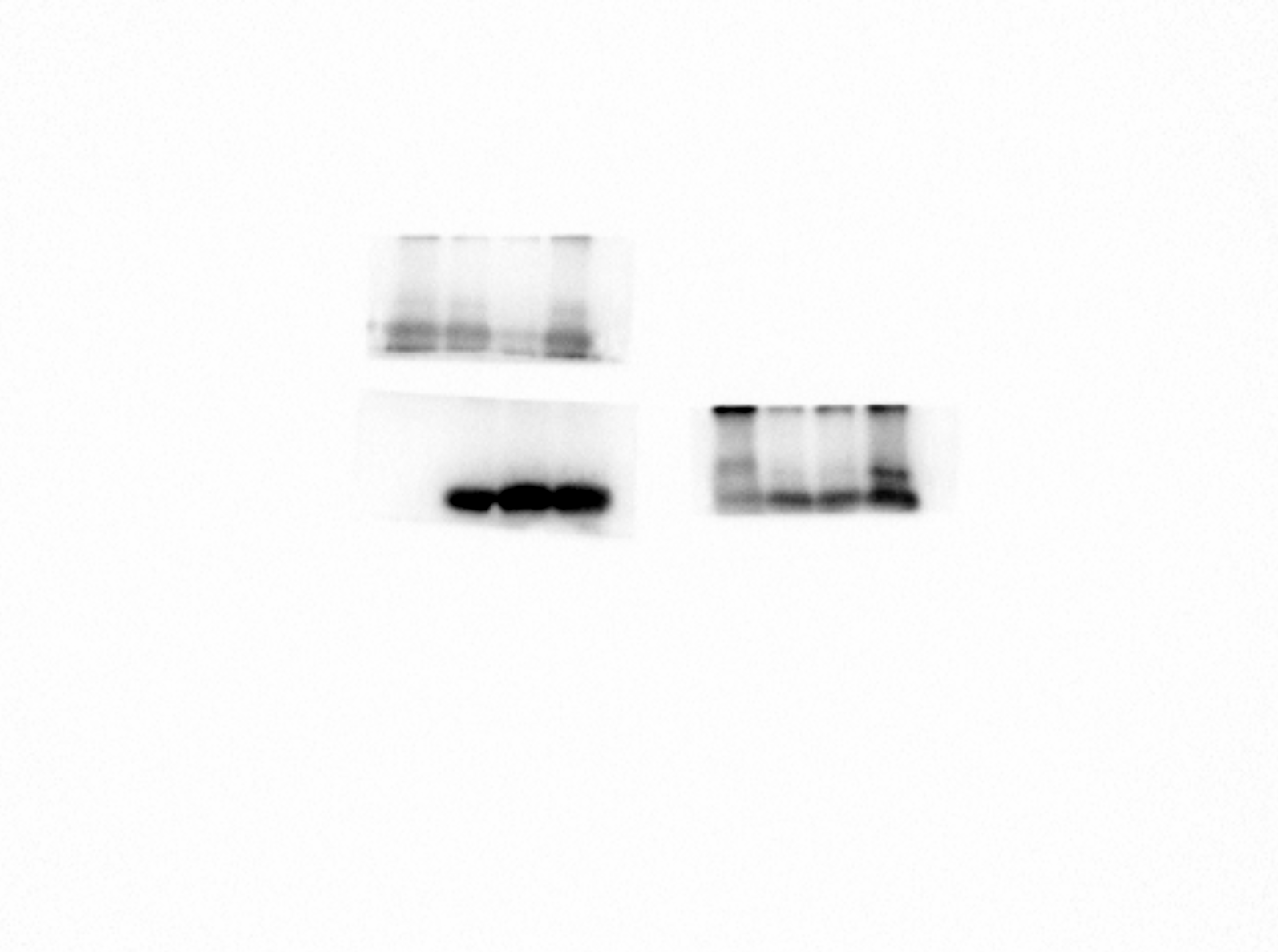

Supplement: Supplementary file 8 [file DataSheet7.ZIP › 4. Podocyte Nucleus Proteins WB scans/Cellular nuclear Histone H3-1.tif]

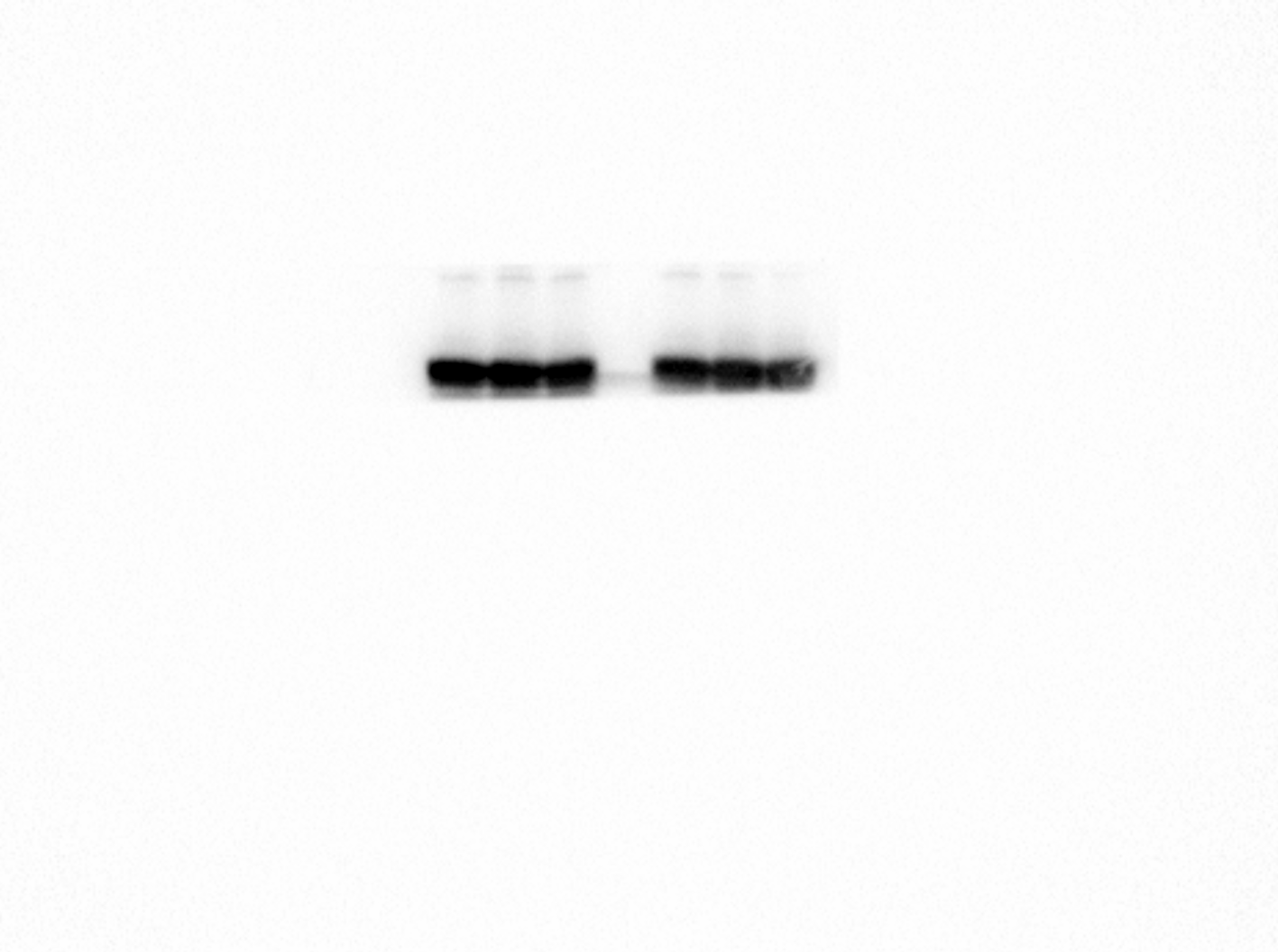

Supplement: Supplementary file 8 [file DataSheet7.ZIP › 4. Podocyte Nucleus Proteins WB scans/Cellular nuclear Histone H3-2.tif]

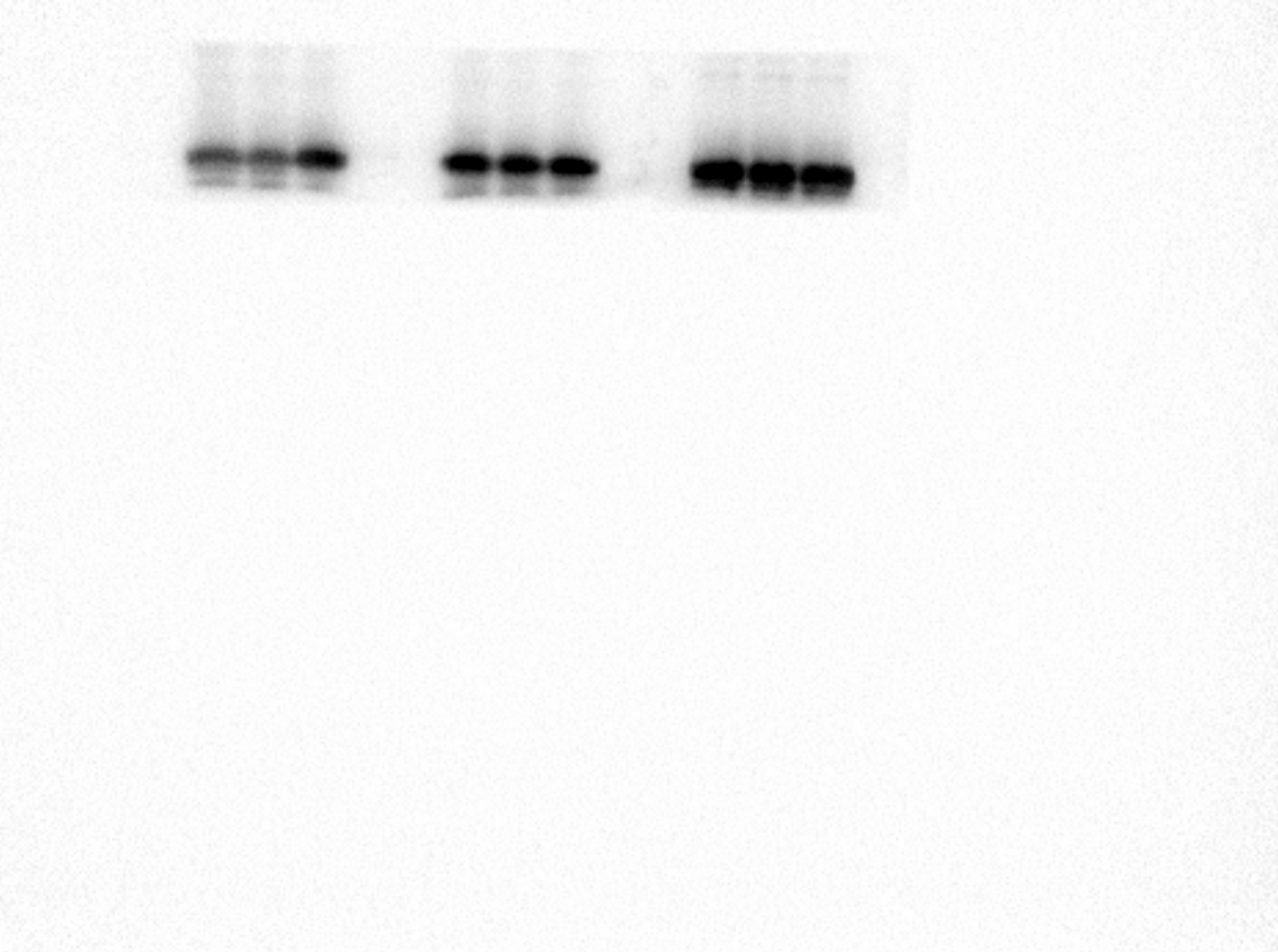

Supplement: Supplementary file 8 [file DataSheet7.ZIP › 4. Podocyte Nucleus Proteins WB scans/Cellular nuclear Histone H3-3.tif]

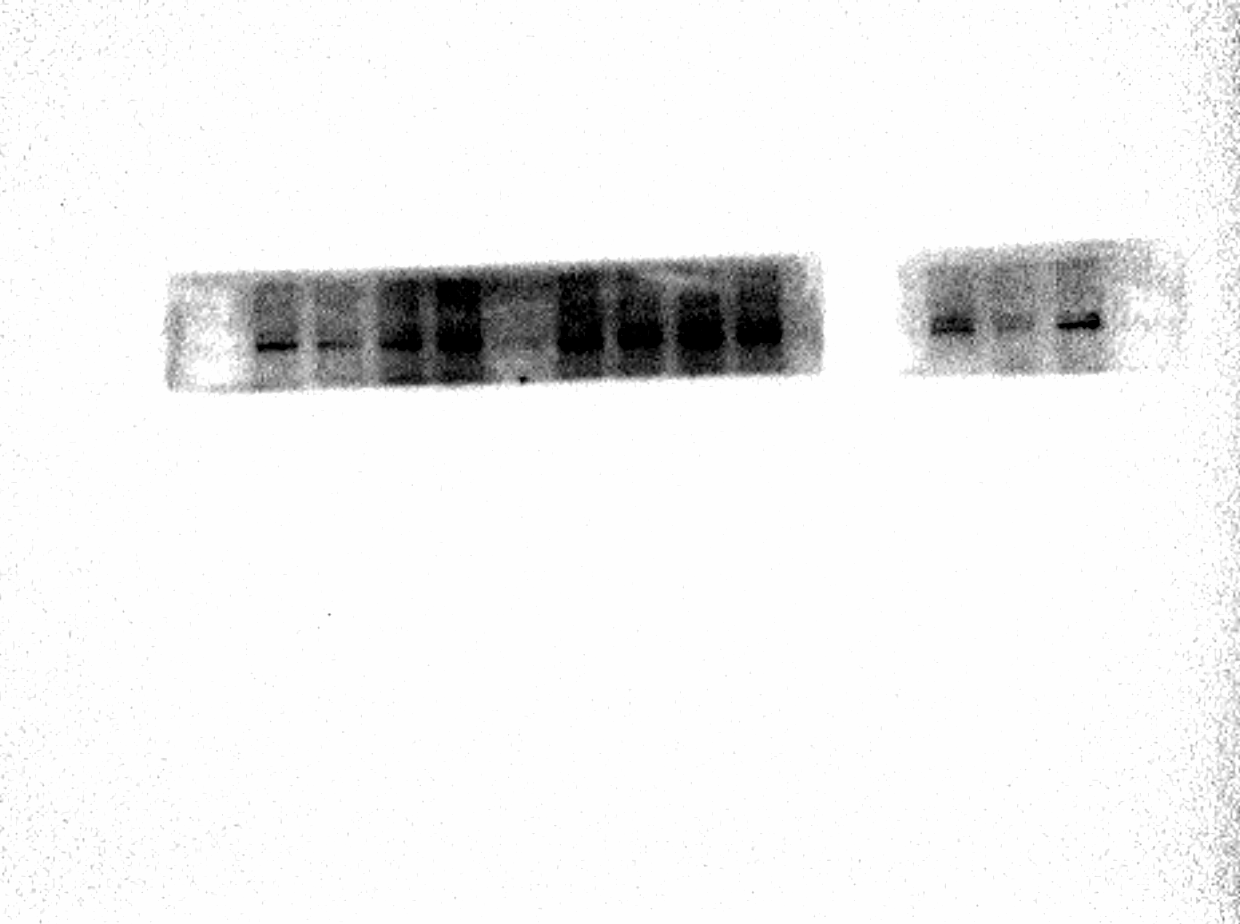

Supplement: Supplementary file 8 [file DataSheet7.ZIP › 4. Podocyte Nucleus Proteins WB scans/cellular nuclear NRF2-3.tif]

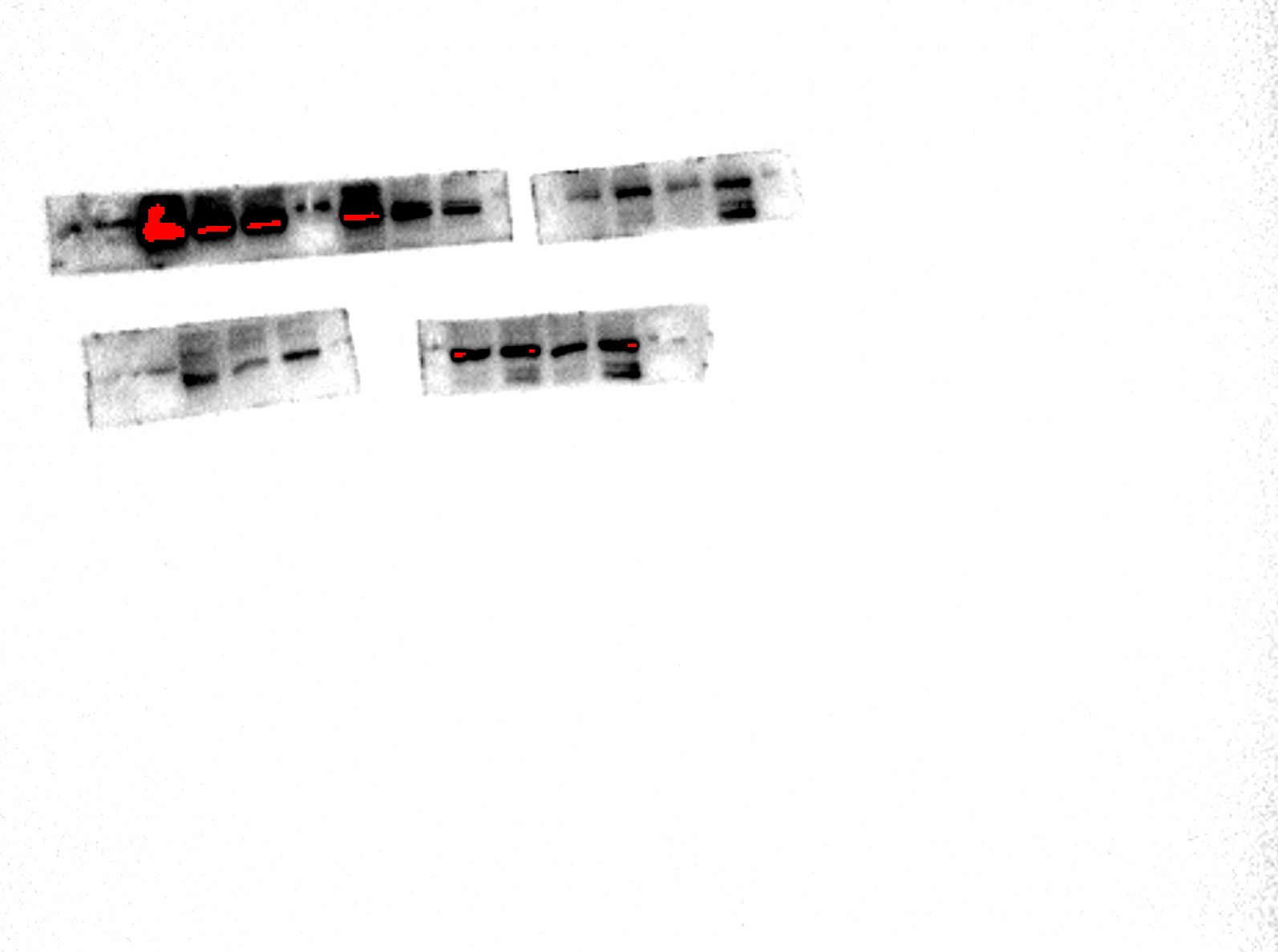

Supplement: Supplementary file 8 [file DataSheet7.ZIP › 4. Podocyte Nucleus Proteins WB scans/cellular nucleus-NRF2-1.tif]

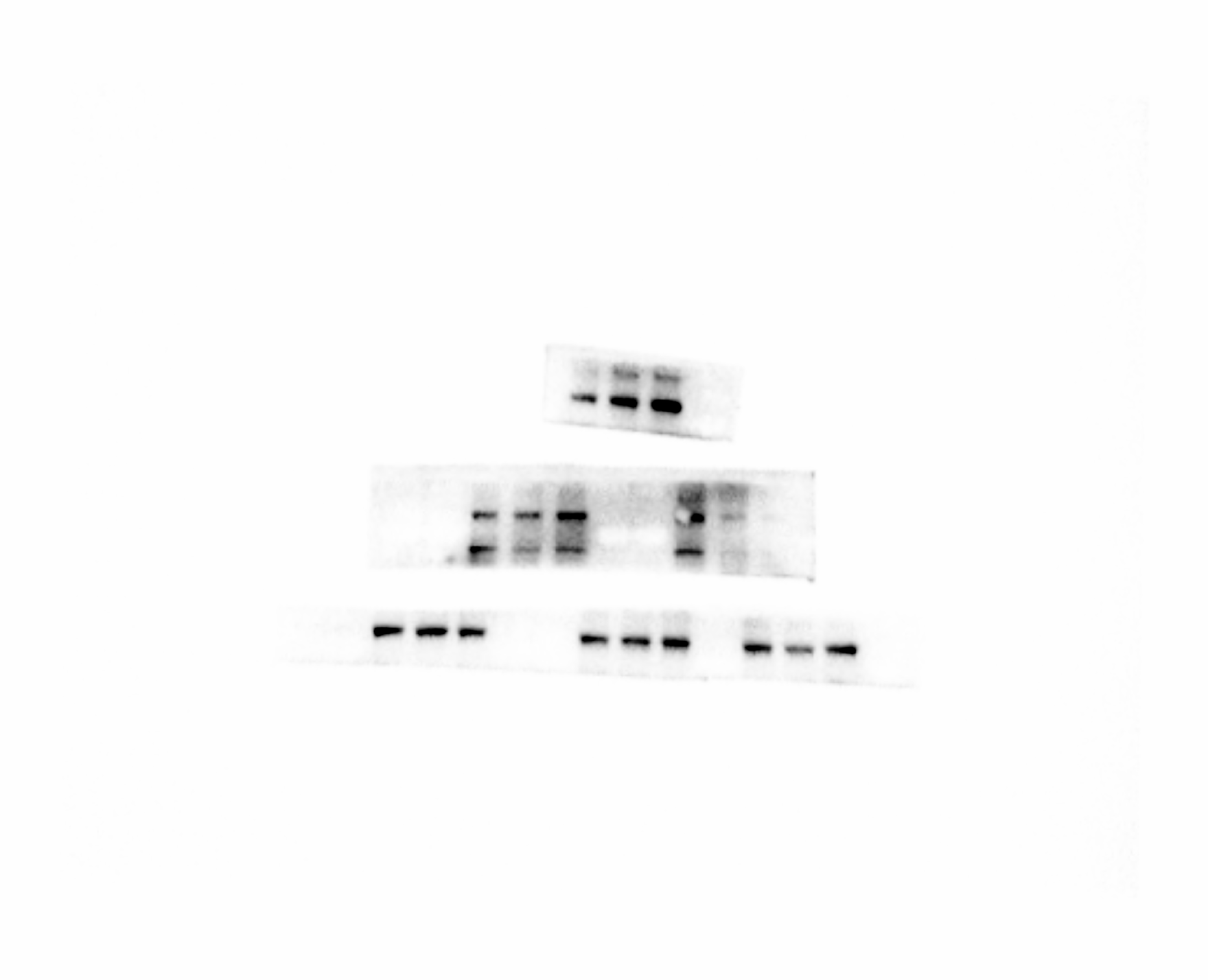

Supplement: Supplementary file 8 [file DataSheet7.ZIP › 4. Podocyte Nucleus Proteins WB scans/cellular nucleus-NRF2-2.tif]
