# Supplementary material for: Sanqi Oral Solution Mitigates Proteinuria in Rat Passive Heymann Nephritis and Blocks Podocyte Apoptosis via Nrf2/HO-1 Pathway
Source: Front Pharmacol. 2021 Nov 19;12:727874. doi: 10.3389/fphar.2021.727874 (PMC8640486; doi:10.3389/fphar.2021.727874)
Supplement: Supplementary file 4 [file Table1.doc]

**Supplementary Table 1.** Component of SQ (三芪口服液).

| **Latin name** | **Botanical name** | **Chinese name** | **Concentration**  (crude drug) |
| --- | --- | --- | --- |
| Radix astragali | *Astragalus mongholicus Bunge [Fabaceae]* | Huang qi (黄芪) | 0.333 g/mL |
| Radix notoginseng | *Panax notoginseng (Burkill) F.H.Chen［Araliaceae］* | San qi (三七) | 0.056 g/mL |
