# Supplementary material for: Sanqi Oral Solution Mitigates Proteinuria in Rat Passive Heymann Nephritis and Blocks Podocyte Apoptosis via Nrf2/HO-1 Pathway
Source: Front Pharmacol. 2021 Nov 19;12:727874. doi: 10.3389/fphar.2021.727874 (PMC8640486; doi:10.3389/fphar.2021.727874)
Supplement: Supplementary file 5 [file DataSheet1.pdf]

Preparation and Chemical Profiles of SQ

MATERIALS AND METHODS

Preparation of SQ

SQ (Batch No. 210102; Cantonese medicine ratification No. Z20071155) was obtained from Guangdong Provincial Hospital of Chinese Medicine and extracted from Radix Astragali and Radix Notoginseng by Water extraction alcohol precipitation method according to the Chinese Pharmacopoeia.

Components Analysis of SQ

The preparation process was implemented refer to our published article(Tian et al., 2020). An Agilent 1200 HPLC system with DAD detector was adopted for quality control analysis of SQ. Briefly, the LC separation was performed over a Kinetex C18 column (4.6 × 100 mm, 2.6 mm, Phenomenex Inc., Torrance, USA) at 30 °C. Samples were eluted by gradients in line with the program of elution as: 0–12 min, 88–80% A; 12–26 min, 80–74% A; 26–40 min, 74 35% A. The UV detection wavelengths were set at 205 and 284 nm.

RESULTS

Component of SQ

As shown in Supplementary Table S1, the concentration of Radix Astragali and Radix Notoginseng in SQ was 0.333 and 0.056 g/mL, respectively.

Chemical Profiles of SQ

As shown in Supplementary Figure S1, 11 chemical profiles of SQ were identified as follows: (1) caylcosin-7-O-β-D-glucopyranoside, (2) ononin, (3) 6aR, 11aR-3-hydroxy-9,10-dimethoxypterocarpan-3-O-β-D- glucopyranoside, (4) calycosin, (5) notoginsenoside R1, (6) isomucronulatol-7-O-β-D-glucopyranoside, (7) ginsenoside Rg1, (8) ginsenoside Re, (9) formononetin, (10) ginsenoside Rb1, (11) ginsenoside Rd.

REFERENCES

Tian, R., Wang, P., Huang, L., Li, C., Lu, Z., Lu, Z., et al. (2020). Sanqi Oral Solution Ameliorates Renal Ischemia/Reperfusion Injury via Reducing Apoptosis and Enhancing Autophagy: Involvement of ERK/mTOR Pathways. *Front Pharmacol* 11**,** 537147. doi: 10.3389/fphar.2020.537147.

TABLE

Supplementary Table 1. Component of SQ (三芪口服液).

Latin name	Botanical name	Chinese name	Concentration (crude drug)
Radix astragali	*Astragalus mongholicus Bunge [Fabaceae]*	Huang qi (黄芪)	0.333 g/mL
Radix notoginseng	*Panax notoginseng (Burkill) F.H.Chen [Araliaceae]*	San qi (三七)	0.056 g/mL

FIGURE

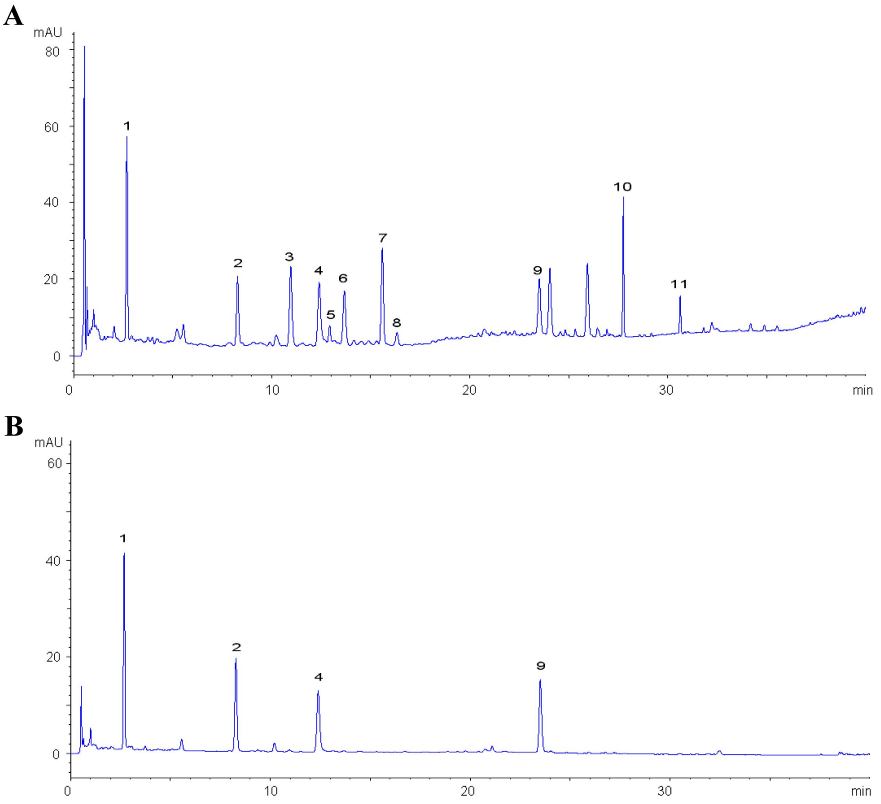

Supplementary Figure 1. Chemical profiles of SQ. UV chromatogram of SQ at 205 nm **(A)** and 284 nm **(B)** was presented. 11 peaks were identified as follows: (1) caylcosin-7-O-β-D-glucopyranoside, (2) ononin, (3) 6aR,

11aR-3-hydroxy-9,10-dimethoxypterocarpan-3-O-β-D- glucopyranoside, (4) calycosin, (5) notoginsenoside R1, (6) isomucronulatol-7-O-β-D-glucopyranoside, (7) ginsenoside Rg1, (8) ginsenoside Re, (9) formononetin, (10) ginsenoside Rb1, (11) ginsenoside Rd.
